# Digital expression explorer 2: a repository of uniformly processed RNA sequencing data

**DOI:** 10.1093/gigascience/giz022

**Published:** 2019-04-03

**Authors:** Mark Ziemann, Antony Kaspi, Assam El-Osta

**Affiliations:** 1 Deakin University, Geelong, Australia, School of Life and Environmental Sciences, 75 Pigdons Road, Waurn Ponds, VIC 3216, Australia; 2 Epigenetics in Human Health and Disease, Central Clinical School, Faculty of Medicine, Monash University, 99 Commercial Road, Melbourne, VIC 3004, Australia; 3 Hong Kong Institute of Diabetes and Obesity, Prince of Wales Hospital, The Chinese University of Hong Kong, 3/F Lui Che Woo Clinical Sciences Building, 30-32 Ngan Shing Street, Sha Tin, Hong Kong SAR

**Keywords:** gene expression, RNA-seq, transcriptome, data reuse

## Abstract

**Background:**

RNA sequencing (RNA-seq) is an indispensable tool in the study of gene regulation. While the technology has brought with it better transcript coverage and quantification, there remain considerable barriers to entry for the computational biologist to analyse large data sets. There is a real need for a repository of uniformly processed RNA-seq data that is easy to use.

**Findings:**

To address these obstacles, we developed Digital Expression Explorer 2 (DEE2), a web-based repository of RNA-seq data in the form of gene-level and transcript-level expression counts. DEE2 contains >5.3 trillion assigned reads from 580,000 RNA-seq data sets including species *Escherichia coli*, yeast, Arabidopsis, worm, fruit fly, zebrafish, rat, mouse, and human. Base-space sequence data downloaded from the National Center for Biotechnology Information Sequence Read Archive underwent quality control prior to transcriptome and genome mapping using open-source tools. Uniform data processing methods ensure consistency across experiments, facilitating fast and reproducible meta-analyses.

**Conclusions:**

The web interface allows users to quickly identify data sets of interest using accession number and keyword searches. The data can also be accessed programmatically using a specifically designed R package. We demonstrate that DEE2 data are compatible with statistical packages such as edgeR or DESeq. Bulk data are also available for download. DEE2 can be found at http://dee2.io.

## Background

Since its first description 10 years ago, RNA sequencing (RNA-seq) has become a powerful method in transcriptomics, allowing highly accurate gene expression quantification [1]. As the cost of sequencing decreases, RNA-seq data are becoming more ubiquitous in the scientific literature. It is standard practice in the field and a compulsory requirement for journals to deposit these data to Gene Expression Omnibus (GEO) and Sequence Read Archive (SRA) [2,3] in the form of raw and processed files, with the aim of fostering greater reuse and transparency. In practice, however, there are several hurdles that impede widespread reuse by biologists. First, processing raw sequence data from SRA requires significant computational resources and command-line expertise. Second, the processed RNA-seq data hosted by GEO are prepared in assorted formats that utilize various software tools and genome annotation sets, which complicates meta-analyses. Despite the value of these data to the scientific community and tremendous cost to generate them, RNA-seq data aggregation efforts have been largely limited to human and mouse [4,5] or are closed source/subscription services [6]. BgeeDB provides array and sequencing-based expression data on many animal species with a particular focus on high-quality measurements of baseline samples at different life stages (excluding disease, treatments, or genetic perturbations) [7]. Expression Atlas is one of the most comprehensive repositories of processed expression microarray data with an informative graphical interface, but only a comparatively small number of RNA-seq data sets are currently included [8]. In an effort to boost reuse of public transcriptome data, we developed Digital Expression Explorer 2 (DEE2), an open-access web-based repository of uniformly processed RNA-seq digital gene-level and transcript-level expression data for several major organisms that is compatible with many types of downstream analyses.

## Data Processing

**Figure 1:**
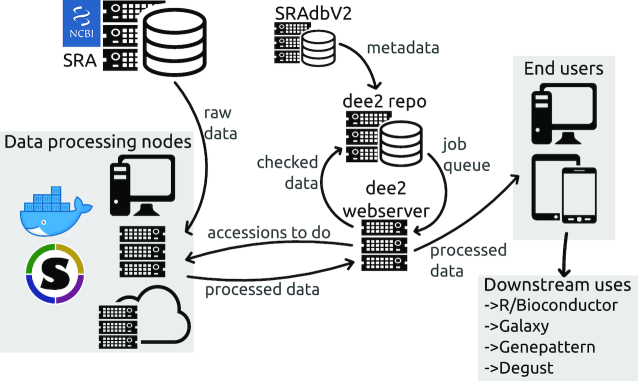
Overview of RNA-seq data processing, storage, and provision.

DEE2 consists of 3 parts: (i) a pipeline that downloads and process raw data sets from SRA; (ii) a data repository where processed files are collected, filtered, and organized/stored and job queues are generated; and (iii) a web server where users can search metadata and obtain data sets of interest. A schematic diagram of the organization of DEE2 is provided in Fig. 1. Data processing nodes request SRA run accession numbers from the web server and obtain raw data from SRA. Processed data are sent to the web server, validated, and relayed to the DEE2 repository server. The repository server performs further validation checks, incorporates new data sets into the repository, collects corresponding metadata from SRAdbV2 [9], and queues outstanding jobs. The repository server then sends updated metadata and job queue information to the web server. End users obtain data from the web browser, command line, or bulk dumps.

## Pipeline Features

The DEE2 pipeline uses containerization to enable rapid application deployment and guarantees analytical reproducibility across different computer systems. End users can run the Docker image [10] on their own hardware to process SRA data sets of interest as specified with a species name and SRA run accession. After completion of the processing, users will have immediate access to the outputs, and after validation by the DEE2 repository server, the data sets will be available publicly. In this way, power users obtain benefit by using an established analysis pipeline and simultaneously contribute to expanding the public resource. One concern with Docker images is that they cannot be run without administrator "root" permissions, e.g., by users of a shared high-performance computing system. To address this limitation, the image can be converted for use by Singularity [11] or UDocker [12] without root permissions.

**Figure 2:**
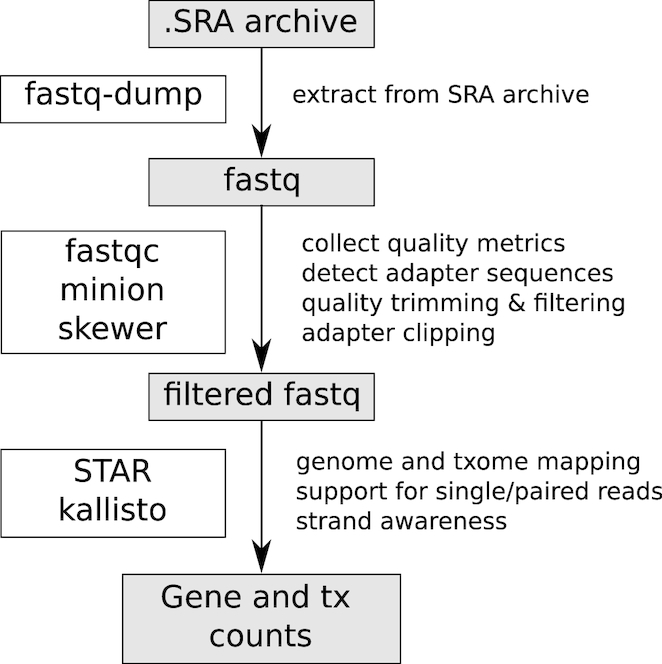
Overview of steps in the RNA-seq data processing pipeline.

The steps involved in data processing are summarized in Fig. 2. The pipeline fetches the appropriate reference genome, annotation, and complementary DNA sequence data from Ensembl (August 2017 version) [13]. Transcriptome sequencing data sets are downloaded from SRA using Aspera. The pipeline handles both single-end (SE) and paired-end (PE) sequencing data with the exclusion of colorspace sequence data. A sample of 4,000 reads is used to perform basic checks including read and quality string format using FastQC [14] prior to extraction of fastq files with a parallel implementation of fastq-dump [15]. Skewer [16] is used to trim bases with phred quality <10 on the 3′ ends and discards reads shorter than 18 nucleotides. Adapter sequences at the 3′ end are detected using Minion, part of the Kraken package [17]. Adapter sequences are clipped using Skewer if the predicted adapter sequence is not present in the genome and exceeds a frequency of 2.5%. To handle nonreference 5′ bases including unique molecular identifiers, a sample of 10,000 reads undergo progressive clipping of 5′ ends (4, 8, 12, 20 nucleotides) followed by genomic mapping with STAR to determine the optimal number of bases to clip from the 5′ end, as determined by the proportion of uniquely mapped reads. STAR [18] is then used to map all reads that pass quality control (QC) to the genome and generate gene-wise expression counts with the "–quantMode GeneCounts" (no alignment files are generated). STAR output is also used to diagnose whether the data set is strand specific. To classify a data set as strand specific, there needs to be a 5:1 strand bias in assigned reads according to STAR. This option is passed to Kallisto, which maps reads to the transcriptome to generate estimated transcript counts [19]. Gene and transcript counts, along with analysis logs and QC metrics, are zipped and transferred to the web server by sftp. The pipeline has the added ability to process users' own fastq files using the same pipeline, although the results remain private. The pipeline code is open source and available online [20]. Software versions and parameters used in the pipeline are provided in Supplementary Table S1.

## Data Provided

Currently DEE2 hosts data from 9 organisms selected because they are important model organisms and have a large number of corresponding transcriptome data sets in SRA. Currently, there are >580,000 RNA-seq data sets available, with each data set corresponding to a specific SRA run number. Together the 9 species included constitute 73.5% of all transcriptome data sets available from SRA.^1^ DEE2 consists of >5.3 trillion assigned sequence reads (Table 1). The data provided include gene-wise expression counts, transcript-wise estimated counts, gene information, transcript information, summary metadata, full metadata, and QC metrics, provided as 7 separate tables in tsv format. The gene information table contains the gene accession number, corresponding gene symbol, and gene length as calculated by GTFtools v0.6.5 [21]. The transcript information file contains transcript-parent gene relationships, gene symbol, and transcript length. Gene and transcript length information will allow straightforward normalization of expression by contig length. The full metadata table contains all corresponding metadata from SRAdbV2, while the summary metadata contains only corresponding SRA accession numbers and experiment title. Moreover, analysis logs for each data set are provided. Classification of data sets by QC metrics is discussed below.

**Table 1:** Hosted gene expression data as of 11 January 2019

Species	Projects	Experiments	Runs	QC classification pass/warn/fail	Assigned reads (STAR)	Assigned reads (Kallisto)
*Arabidopsis thaliana*	986	17,095	26,061	5,602/15,122/5,337	2.87e+11	2.92e+11
*Caenorhabditis elegans*	339	5,759	7,722	1,647/2,446/3,629	8.71e+10	7.88e+10
*Drosophila melanogaster*	678	14,401	18,713	4,410/7,471/6,832	1.75e+11	1.87e+11
*Danio rerio*	457	26,246	28,100	1,084/5,826/21,190	1.11e+11	6.20e+10
*Escherichia coli*	180	1,488	1,638	355/376/907	1.26e+10	9.40e+09
*Homo sapiens*	6,768	197,836	229,634	42,225/77,254/110,155	2.27e+12	2.51e+12
*Mus musculus*	7,078	204,850	252,058	23,840/85,874/142,344	1.84e+12	2.08e+12
*Rattus norvegicus*	349	4,965	5,799	426/2,651/2,900	5.95e+10	6.42e+10
*Saccharomyces cerevisiae*	442	10,239	11,369	3,025/2,783/5,561	7.41e+10	7.32e+10
Total	17,277	482,879	581,094	82,614/199,803/298,855	4.92e+12	5.35e+12

## Quality Control Metrics

**Figure 3:**
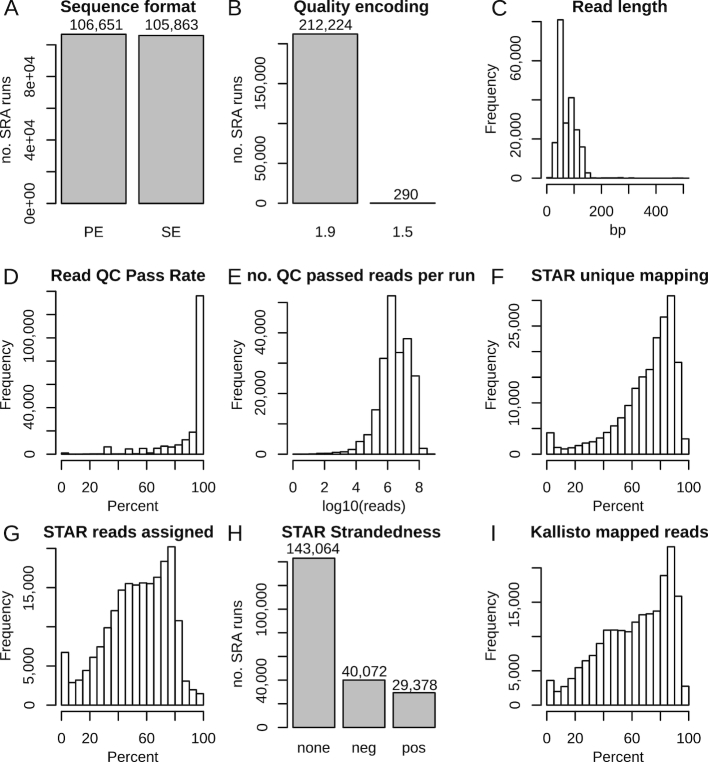
Summary QC metrics for human data sets. (A) sequence format. (B) Base quality encoding, Illumina version 1.9 and 1.5. (C) Read length histogram. (D) Proportion of reads that pass QC filtering. (E) Number of QC passed reads per run. (F) Proportion of STAR uniquely mapped reads. (G) Proportion of reads assigned to genes. (H) Classification of reads by strandedness. (I) Proportion of reads mapped with Kallisto. Data accessed 20 December 2018.

QC is paramount for a resource such as this. A range of quality metrics are accessible and can be viewed on the search results page, which includes mean base quality scores, number of reads, alignment rates, and read assignment statistics. Detailed analysis logs are distributed alongside expression data. Summary statistics for human data sets are provided in Fig. 3. There are roughly equal numbers of runs with SE and PE sequencing (Fig. 3A). Almost all data sets are encoded in Illumina 1.9 format (also known as Sanger), and a small number of data sets with Illumina 1.5 quality encoding (Fig. 3B). The median read length is 75 base pairs ( bp) and mode is 50 bp (Fig. 3C). Most (71.3%) data sets had ≥95% of reads pass QC filtering (Fig. 3D). The median number of reads passing QC filtering was 4.6 million (Fig. 3E). The median proportion of STAR unique mapping was 82% (Fig. 3F). Median assignment proportion recorded a median of 59.4% (Fig. 3G). The majority of data sets were classified as unstranded (67.3%), with smaller numbers of runs biased towards each strand (Fig. 3H). The median proportion of reads mapped with Kallisto was 63.2% (Fig. 3I).

Although there are no definitive thresholds for what constitutes a valid RNA-seq data set, there are 2 main principles: (i) the digital nature of RNA-seq means that data sets with more reads will provide more accurate quantification, and (ii) data sets with a large proportion of reads excluded from downstream analysis will be less representative of the original sample, and suggest issues with sample quality, library preparation, or sequencing instrumentation. Using these principles, we have classified the data sets as “pass,” “warn,” and “fail” according to heuristics outlined in Table 2. Each rule has a numeric code, and this is provided in the search results. Because sequencing depth recommendations are larger for more complex organisms, the metrics describing integer counts are proportional to transcriptome complexity (the number of protein-coding genes as defined by Ensembl).

**Table 2:** Criteria for data set quality classification

Metric	Meaning	Fail threshold	Warn threshold	Code
NumReadsQcPass	No. reads passed QC filtering	<50 reads per gene^a^	<500 reads per gene^a^	1
QcPassRate	Proportion of reads passed QC filtering	<60%	<80%	2
STAR_UniqMapRate	Proportion of reads mapped uniquely to the reference genome using STAR	<50%	<70%	3
STAR_AssignRate	Proportion of reads assigned to genes with STAR	<40%	<60%	4
STAR_AssignedReads	No. reads assigned to genes with STAR	<50 reads per gene^a^	<500 reads per gene^a^	5
Kallisto_MapRate	Proportion of reads assigned to transcripts with Kallisto	<40%	<60%	6
Kallisto_MappedReads	No. reads assigned to transcripts with Kallisto	<50 reads per gene^a^	<500 reads per gene^a^	7
DatasetCorrel	Pearson correlation coefficient to passed data average	–	<0.5	8

a Number of protein-coding genes was obtained from Ensembl and used as an estimator of transcriptome complexity.

Furthermore, if a data set profile is substantially different to the bulk of “pass” data sets, this may be useful information for end users. To quantify this, an average gene expression profile (STAR) of “pass” data sets is calculated and each data set is compared by Pearson correlation (Methods). If the correlation coefficient is <0.5, then the data set is flagged as “warn.” As new data sets are periodically added, these correlation values may vary slightly over time.

**Figure 4:**
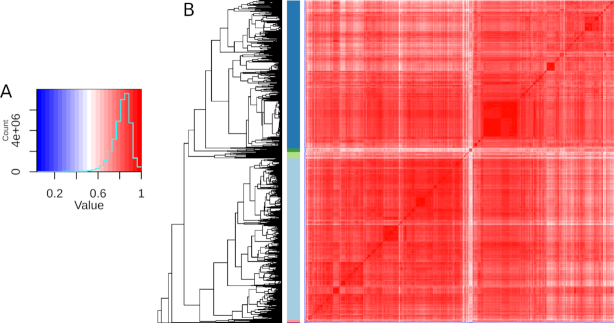
Unsupervised clustering analysis of the correlation of 5,807 *S. cerevisiae* data sets. (A) Colour key and histogram of Spearman correlation coefficients. (B) Heat map of pairwise correlation values with data sets clustered by similarity. Red indicates high correlation and blue indicates low correlation.

To understand why some data sets have low correlation to the bulk of "pass" data sets, we undertook an unsupervised clustering analysis of correlation in 5,808 *S. cerevisiae* data sets. While most Spearman correlation coefficients were >0.7, there was a small fraction <0.5 (Fig. 4A). Most data sets (5,571) were classified into 2 large clusters (Fig. 4B; blue and light blue). The remaining 236 data sets belonged to several smaller clusters. These smaller clusters mostly contained data sets derived from nonstandard RNA-seq library construction protocols such as 3′ end RNA-seq (ERP004367, SRP048715, SRP048715, SRP021938), Ribo-Seq (SRP075766, SRP082147), and RNA-IP-Seq (SRP032276). One of the smaller clusters contained data sets of cells undergoing sporulation and meiosis (e.g., SRP092588, SRP061166, SRP032309). From this analysis, we can conclude that highly correlated data sets are standard RNA-seq/mRNA-seq and data sets with low correlation are mostly due to the use of nonstandard library construction protocols, but also some data sets derived from less characterized biological states (e.g., meiosis/sporulation in the case of *S. cerevisiae*).

## Pipeline Validation

**Figure 5:**
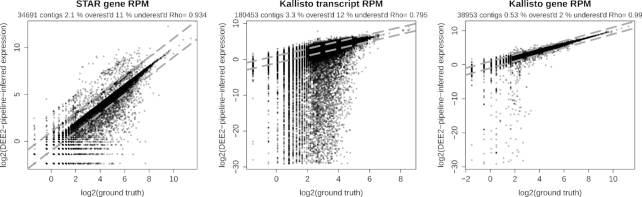
Comparison of ground truth and DEE2 pipeline inferred expression profiles. Human SE 100 bp RNA-seq reads simulated with ART [24] underwent mapping with the DEE2 pipeline, generating gene-level and transcript-level expression counts. Inferred expression count values were normalized for library size and plotted against the corresponding ground truth values. Dashed lines show the 2 and –2 fold expression differences. STAR gene counts were generated with the –quantMode GeneCounts feature. Kallisto-estimated counts were used to quantify transcripts. Kallisto gene counts were calculated by aggregating (sum) estimated transcript counts to their parent gene.

To demonstrate the accuracy of the pipeline, we performed a simulation study. Synthetic Illumina HiSeq RNA-seq data were generated from Ensembl transcripts and processed with the pipeline (see Methods). The reads per million (RPM) values were compared between the ground truth and DEE2-processed data, and Spearman correlation coefficients (ρ) were calculated (Fig. 5; Supplementary Table S2). We observed that analyses of simpler organisms were, in general, more accurate than for more complex transcriptomes of human and mouse. Overall there was only a small improvement in accuracy in PE over SE reads. Transcript quantification results from Kallisto were less accurate than gene level quantification with STAR. On the other hand, Kallisto transcript counts collapsed into their parent gene were substantially more accurate than STAR gene counts (Fig. 5; Supplementary Table S2), consistent with previous a previous report [22].

**Figure 6:**
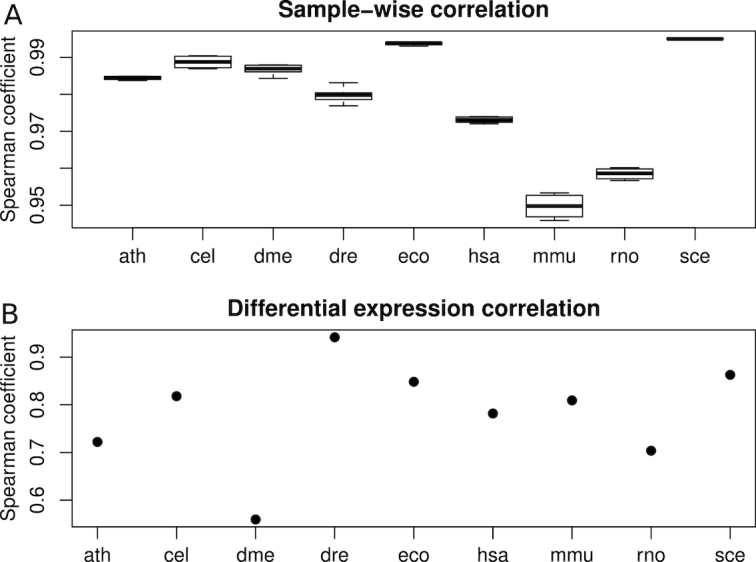
Comparison of DEE2 data with author-uploaded gene-level count data. (A) Sample level RPM correlation between author-provided and DEE2-processed expression profiles. (B) Correlation of differential expression results. Ath: *A. thaliana*; cel: *C. elegans*; dme: *D. melanogaster*; dre: *D. rerio*; eco: *E. coli*, hsa: *H. sapiens*; mmu: *M. musculus*; rno: *R. norvegicus*; sce: *S. cerevisiae*.

In a separate validation exercise, we compared author-supplied expression count data present in GEO with corresponding DEE2-STAR counts, and quantified the similarity at the level of individual runs as well as across contrasts (see Methods for details). At the level of individual runs, there was a tight correlation between DEE2-derived and author-supplied RPM values, with Spearman coefficients in the range of 0.95–0.99 (Fig. 6A). After differential expression analysis with edgeR [23], genes were ranked by significance. Author-derived differential expression results were then compared with DEE2-derived differential expression results, enabling us to generate a single Spearman correlation coefficient for each contrast. Using this approach, the correlation in differential expression results between DEE2-STAR and author-supplied counts ranged between 0.55 and 0.95, with a median of 0.81 (Fig. 6B). Differential expression correlation was higher in comparisons with more replicates (ρ = 0.757, n = 9, *P* = 0.018). Both exercises support the validity of DEE2 data.

## A Brief Meta-analysis of Yeast Gene Expression

**Figure 7:**
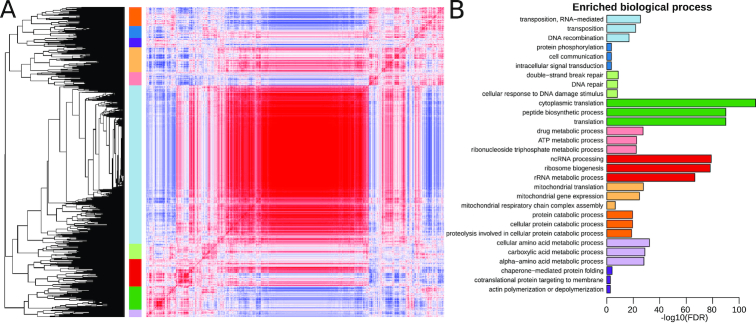
Unsupervised hierarchical clustering of gene expression in *S. cerevisiae*. (A) Clustering and correlation heat map. Red indicates high correlation and blue indicates low correlation. (B) Biological process gene ontology enrichments of each cluster. Only the top 3 biological processes are shown (as ranked by significance).

To demonstrate the utility of DEE2 data we undertook an exploratory analysis of gene expression in *S. cerevisiae*. We correlated the expression of all genes in 5,808 data sets in DEE2 and performed unsupervised hierarchical clustering. This resulted 7,126 genes being classified into 10 clusters (Fig. 7A). The largest cluster consisted of 3,634 genes (light blue), and the remaining clusters contained between 634 and 175 genes. Gene ontology analysis was performed to detect overrepresented biological pathways in each cluster (Fig. 7B). Interestingly, each cluster was involved in different biochemical specializations. For example, the dark green cluster was overrepresented in genes involved in translation, while the nearest neighbour, light purple, was overrepresented in amino acid metabolism. Similarly the light orange cluster was enriched for genes involved in mitochondrial function and the nearest neighbour, pink, was involved in adenosine triphosphate metabolism. These findings illustrate one way in which DEE2 facilitates meta-analysis of gene expression.

## Reuse Potential

The financial cost of generating these raw data sets is substantial. A rough estimate of the cost to generate raw data included in DEE2 is ∼$162 million US.^2^ In contrast, the estimated cost to process these data sets on Amazon EC2 infrastructure is estimated at just $97,000 but could be reduced to ∼$24,000 using off-peak resources.^3^ Therefore, data aggregation efforts like DEE2 can, with a modest budget, add substantial value to these existing data by enabling straightforward reuse. Another benefit of aggregation is that genome annotations are updated over time as compared to author-submitted data that remain static.

**Figure 8:**
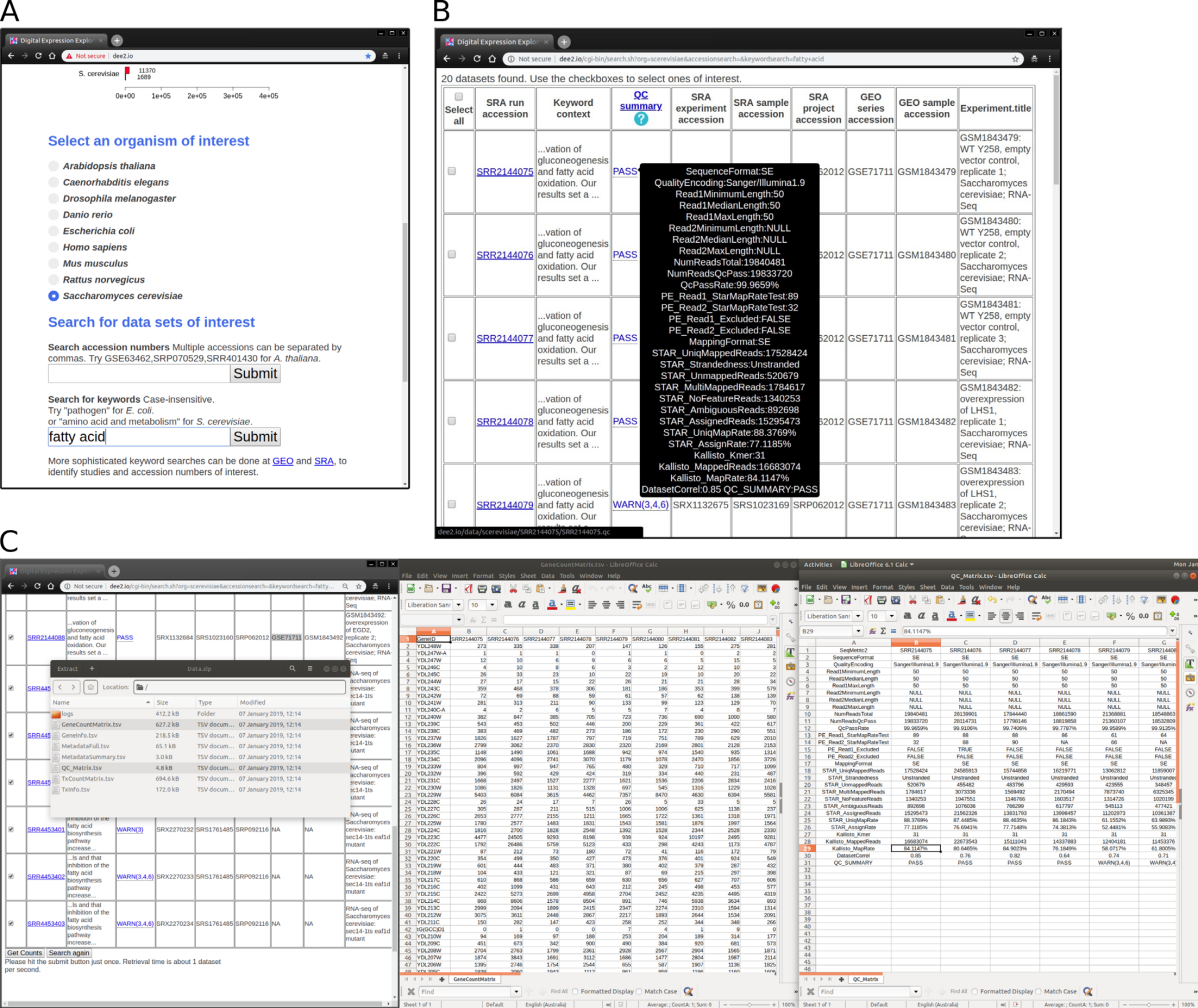
Example of the DEE2 web interface. (A) Users select the species of interest and supply either accession numbers or keywords to search. (B) In this case, a search for "fatty acid" for *S. cerevisiae* yields 20 hits. Mouse pointer hovering over the QC summary field shows the key quality criteria of the processed data sets. (C) Users can select the data sets to download by ticking the boxes and hitting the "Get counts" button at the bottom of the page. The downloaded zip file (C left side) contains detailed processing logs of each run, along with a matrix of gene-wise counts, transcript-wise counts, metadata information, QC information, and information on transcripts and genes. The gene-wise count matrix and QC information are shown in C, middle and right side, respectively.

To enhance the reuse potential, we have designed a simple and easy-to-use website to access the data. Users select 1 of the 9 species featured and provide either keywords or accession numbers to identify data sets of interest (Fig. 8A). The web interface provides data sets in batches of ≤500 runs. When 501–5000 matches are obtained, users can download the corresponding metadata and are given options to access expression data (see below). The search results page contains corresponding SRA accession numbers, experiment title, and keyword context if a keyword was used (Fig. 8B). The results page provides links to QC information so that users can be assured of data set quality. If ≤500 matches are found, the QC information can be seen simply by hovering the mouse over the QC summary field (Fig. 8B). Users then tick the box of every data set they would like to download, and by hitting the "Get Counts" button, the data sets are downloaded. The searching and retrieval steps for the example depicted in Fig. 8 took 13 seconds. Fig. 8C demonstrates how data are delivered to end users: as a zip archive containing tab-separated expression count, contig information, metadata, and QC information. The web server is limited to fetching 500 data sets at a time. To enable easy access to large data sets we provide zip "bundles" for each project with ≥200 runs that have been fully processed by DEE2; there are 425 such bundles as of 11 January 2019 [27].

Because R is the main language for downstream statistical analysis of RNA-seq data, we also provide an R package getDEE2 to obtain DEE2 data. In the example shown in Box 1, data sets belonging to an experiment with GEO series GSE33569 are obtained. Transcript-wise counts can be aggregated to gene-level counts with a single command (Tx2Gene).

**Box 1:**
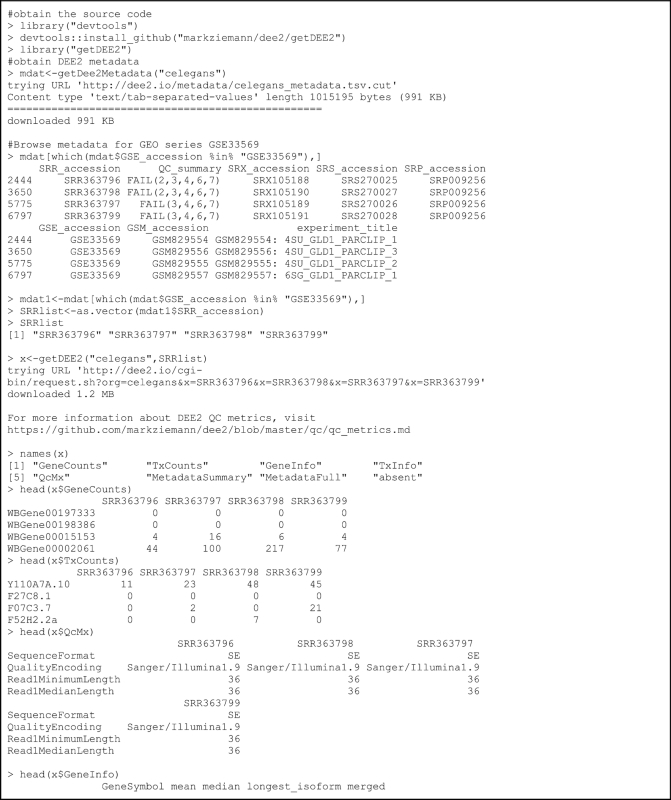

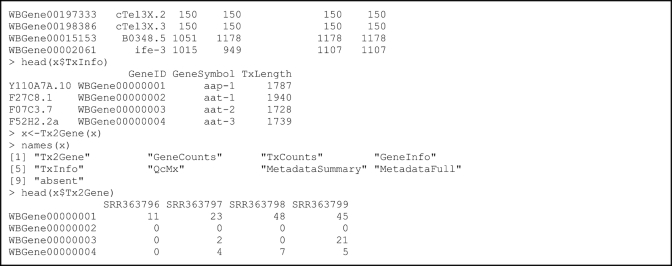
An example of obtaining gene and transcript expression data sets using the R functions (GEO series: GSE33569). The Tx2Gene function is used to aggregate (sum) transcript counts to gene-level counts.

For power users, bulk data dumps are available and will be of use to researchers wishing to do wholesale meta-analyses, as 3 studies already have [28–30]. Irrespective of the method of acquisition, DEE2 data are compatible with many different downstream applications including R/Bioconductor [31,32], Degust [33], and Galaxy [34].

## Conclusion

DEE2 provides a unique framework and user-friendly resource of processed RNA-seq data that alleviates many of the bottlenecks researchers currently face with analysis of public RNA-seq data. Our testing shows that DEE2 and Degust enable analysis of public RNA-seq data on mobile devices such as smartphones. Bulk data provided by DEE2 are a useful starting point for researchers performing meta-analyses of RNA-seq data.

## Methods

### Analysis of correlation

Correlation of a data set to the bulk of other high-quality data sets could be a useful metric to filter by when performing meta-analyses. To make this a tractable calculation, first a mean gene expression profile is generated using STAR gene expression data from ≤10,000 randomly selected data sets that pass all other QC checks. Next, each data set is compared to the average pass profile and a Pearson correlation coefficient is calculated. As new data sets will be added to DEE2 regularly, the average pass profile will vary slightly over time and as such, the correlation coefficient is calculated to 2 significant figures only.

In the analysis of data set correlation (Fig. 4), the Spearman correlation of all 5,808 *S. cerevisiae* data sets classified as "pass" and "warn" was calculated in a pairwise fashion (data as of 3 January 2019). Clustering was performed using the hclust function in R with the complete linkage method, followed by tree cutting at 0.375 of the maximum branch length. The heatmap.2 package was used to generate the heat map. In the analysis of gene expression correlation (Fig. 7A), the *S. cerevisiae* data were transposed prior to correlation analysis, then tree cutting was performed at 0.17 of the maximum branch length. The clusters obtained were subjected to gene ontology analysis at the level of biological pathways using the enrichGO tool in the clusterProfiler package version 3.8.1 (clusterProfiler, RRID:SCR_016884) [35]. Only the top 3 pathways for each cluster were plotted.

### Pipeline validation using simulated data

To validate the accuracy of the DEE2 pipeline, we generated Illumina HiSeq2500-like sequence reads from Ensembl complementary DNA sequences using ART (v2016-06-05) [24] with a defined seed (1,540,165,885) and a uniform fold coverage of 2. Read lengths were 50 and 100 bp in SE and PE format, respectively. The read sets were processed with the DEE2 pipeline and the observed expression data were compared with the ground truth, using Spearman correlation of library size normalized profiles in RPM as an indicator of accuracy. For Kallisto-based analysis, estimated transcript counts "est_counts" were used. Transcript-estimated counts were totalled for each parent gene to generate gene-wise expression counts. These analyses were performed for all 9 organisms currently included in DEE2.

### Pipeline validation using public data

Another way to validate the accuracy of DEE2 data is to compare with author-submitted results available on GEO. We searched for studies that reported expression data as raw counts with official gene names or Ensembl accession numbers, ≥2 replicates, and acceptable read depth and genome mapping rate. Author-supplied counts were obtained from GEO for the data sets listed in Table 3 [36–44]. Spearman correlation of RPM values was used to quantify the similarity of DEE2 and author-supplied data at the level of individual runs. To determine the similarity in differential expression results, the same edgeR v3.22.3 [23] analysis was performed in parallel on author-supplied counts and DEE2 counts. The runs defined as control and case for each experiment are listed in Table 3. To rank genes by significance in differential expression, the sign of the fold change was multiplied by the negative log2 *P*-value. Spearman correlation analysis was used to quantify the similarity in differential expression results using these 2 data sources.

**Table 3:** Details of author-supplied processed data used to compare to DEE2 gene expression counts

Species, GEO series	Contrast (control/case)	Spots	Author pipeline
*A. thaliana*, GSE53078 [36]	GSM1281703	15,143,653	Genome: TAIR10
	GSM1281704	12,498,123	Annotation version: Unknown
	GSM1281705	22,721,359	Mapper: TopHat
	GSM1281706	17,255,612	Counter: HTSeq
*C. elegans*, GSE46344 [37]	GSM1128862	30,650,959	Genome: WS220/ce10
	GSM1128863	47,245,721	Annotation: Ensembl v66
	GSM1128864	54,573,311	Mapper: TopHat
	GSM1128868	49,315,179	Counter: HTSeq
	GSM1128869	56,295,663	
	GSM1128870	68,641,842	
*D. melanogaster*, GSE43180 [38]	GSM1057982	24,902,977	Genome: dm3
	GSM1057983	36,434,276	Annotation: Ensembl v64
	GSM1057984	32,591,508	Mapper: Tophat
	GSM1057985	35,375,654	Counter: HTSeq
*D. rerio*, GSE80768 [39]	GSM2136810	19,404,674	Genome: Zv10
	GSM2136811	22,820,115	Annotation: Ensembl (version unknown)
	GSM2136812	25,181,184	Mapper: USeq and Novoalign
	GSM2136813	21,487,514	Counter: USeq
	GSM2136814	24,831,643	
	GSM2136815	22,664,352	
	GSM2136816	22,629,782	
	GSM2136817	21,842,104	
	GSM2136818	20,601,291	
	GSM2136819	18,183,746	
	GSM2136820	21,007,467	
	GSM2136821	20,992,396	
	GSM2136822	24,708,106	
	GSM2136823	21,105,462	
	GSM2136824	28,069,482	
*E. coli*, GSE80251 [40]	GSM2122743	5,221,858	Genome: *E. coli*K12 MG1655
	GSM2122744	6,503,454	Annotation: GenBank NC_000913.3
	GSM2122745	6,209,263	Mapper: TMAP (map4)
	GSM2122746	6,391,549	Counter: Bedtools
	GSM2122747	6,197,872	
	GSM2122748	5,090,669	
*H. sapiens*, GSE63776 [41]	GSM1556982	30,007,994	Genome: hg19
	GSM1556983	27,252,897	Annotation: UCSC (version unknown)
	GSM1556984	42,212,497	Mapper: Bowtie2 (after adapter clipping)
	GSM1556985	31,456,271	Counter: HTSeq
	GSM1556986	31,569,339	
	GSM1556987	37,477,777	
*M. musculus*, GSE59970 [42]	GSM1462883	32,015,112	Genome: GRCm38.70/mm10
	GSM1462884	30,997,187	Annotation: Ensembl v70
	GSM1462885	32,612,584	Mapper: Olego
	GSM1462886	31,485,760	Counter: BedTools
	GSM1462887	30,207,461	
	GSM1462888	31,028,501	
*R. norvegicus*, GSE65715 [43]	GSM1604049	42,296,446	Genome: rn4
	GSM1604050	34,887,323	Annotation: Ensembl (version unknown)
	GSM1604051	42,725,865	Mapper: Tophat2
	GSM1604052	28,210,194	Counter: HTSeq
	GSM1604053	30,748,641	
	GSM1604054	28,450,626	
*S. cerevisiae*, GSE76444 [44]	GSM2809655	35,869,614	Genome: EF 4
	GSM2809656	37,425,737	Annotation: Ensembl v72
	GSM2809657	39,227,797	Mapper: Bowtie
	GSM2809658	33,974,055	Counter: HTSeq
	GSM2809659	33,339,067	
	GSM2809660	37,546,069	

## Availability of source code and requirements

- Project name: Digital Expression Explorer 2
- Project home page: http://dee2.io
- Operating systems (data set): Platform independent
- Operating systems (pipeline): Unix and MacOS
- License: GNU GPL v3
- Any restrictions on use by nonacademics: none

## Availability of supporting data and materials

- Data set access: http://dee2.io (RRID:SCR_016929)
- https://dee2.io/bulk.htmlBulk data access:
- Source code: https://github.com/markziemann/dee2 (RRID:SCR_016930)
- Pipeline Docker image: https://hub.docker.com/r/mziemann/tallyup/(RRID:SCR_016931)

A snapshot of the latest update of the bulk data presented in this article is available in the *GigaScience* GigaDB repository [45].

## Additional files

**Supplementary Table S1**. Software versions and parameters used in the pipeline.

**Supplementary Table S2**. Spearman correlation coefficients (ρ) between ground truth and DEE2 processed expression profiles (RPM) from simulated data.

## Abbreviations

bp: base pairs; DEE2: Digital Expression Explorer 2; GEO: Gene Expression Omnibus; PE: paired end; QC: quality control; RNA-seq: RNA sequencing; RPM: reads per million; SE: single end; SRA: Sequence Read Archive; SRAdbV2: An R Package to Query the Sequence Read Archive.

## Competing interests

The authors declare that they have no competing interests.

## Funding

A.E.-.O is a Senior Research Fellow supported by National Health and Medical Research Council (APP1154650). A.E.-O. receives funding from the National Health and Medical Research Council—Natural Science Foundation of China (NHMRC-NSFC International Joint Call APP1113188). A.E.-O receives funding from the National Health and Medical Research Council—European Union Collaborative Research Grants Scheme (APP1075563).

## Author contributions

M.Z. and A.E.-O. conceived and designed the study. M.Z. and A.K. wrote the computer code. M.Z. coordinated data processing and drafted the manuscript. All authors read, revised, and approved the final manuscript.

## Supplementary Material

GIGA-D-18-00444_Original-Submission.pdf

GIGA-D-18-00444_Revision-1.pdf

Response-to-Reviewer-Comments_Original-Submission.pdf

Reviewer-1-Report-Original-Submission -- Lucas Carey 12/4/2018 Reviewed

Reviewer-1-Report-Revision-1 -- Lucas Carey 2/5/2019 Reviewed

Reviewer-2-Report-Original-Submission -- Andrea Rau 12/10/2018 Reviewed

Reviewer-2-Report-Revision-1 -- Andrea Rau 2/4/2019 Reviewed

Supplement_Tables.pdf

## Footnotes

1 These 9 species represent 822,819 of the 1,119,784 RNA experiments present in SRA as of 29 October 2018.

2 Estimated cost (in US dollars) of generating 5.3 trillion 100 bp SE reads from 399,377 experiments. Cornell University Institute of Biotechnology advertises HiSeq2500 at $18 ,407 for 8 lanes 100 bp SE [25]. Illumina HiSeq2500 v4 specifications sheet estimates 1.5 billion reads per 8-lane flow cell [26]. That gives $12.27 per million reads. The cost of sequencing is $65.0 million. Library preparation costs ∼$200 per sample, which is in the range advertised at Cornell multiplied by 482,879 experiments equates to $96.6 million for library construction. Grand total of $162 million.

3 Estimated cost of Amazon EC2 pricing is based on the mean elapsed time for processing a data set being 894 seconds on a 16-thread Intel Xeon E3-12xx v2 (Ivy Bridge, indirect branch restricted speculation) 2.6 GHz, 64 GB RAM with ∼400 MB/s download speed from the National Center for Biotechnology Information. The equivalent Amazon instance is r5d.2xlarge and would require a 500 GB volume. The elapsed time is based on observations from a set of 705 human data sets. The breakdown for the total on demand cost is $83,697 for compute, $131 for data transfer, and $12 ,750 for persistent data volume. The off-peak cost breakdown is $11, 544 for compute, $131 for data transfer, and $12 ,750 for persistent data volume. The persistent data volume cost is valid if using 17 instances continuously for a full year. Compute price is based on eastern US ocation (Ohio) and is more expensive at other locations.
