# Supplementary material for: Digital expression explorer 2: a repository of uniformly processed RNA sequencing data
Source: Gigascience. 2019 Apr 3;8(4):giz022. doi: 10.1093/gigascience/giz022 (PMC6446219; doi:10.1093/gigascience/giz022)
Supplement: GIGA-D-18-00444_Original-Submission.pdf [file giz022_giga-d-18-00444_original-submission.pdf]

GigaScience
Digital Expression Explorer 2: a repository of uniformly processed RNA sequencing data
--Manuscript Draft--

**Manuscript Number:**	GIGA-D-18-00444
**Full Title:**	Digital Expression Explorer 2: a repository of uniformly processed RNA sequencing data
**Article Type:**	Data Note
**Funding Information:**	National Health and Medical Research Council (APP1113188) — Prof Assam El-Osta
**Abstract:**	Background: Since the advent of RNA-seq 10 years ago, it has become an indispensable tool in the study of gene regulation. Although RNA-seq has brought with it better transcript coverage and quantification, there remain considerable barriers-to-entry for the novice computational biologist to analyze these large data sets. There is a real need for a repository of uniformly processed RNA-seq data that is easy to use.<br>Findings: To address these obstacles, we developed Digital Expression Explorer 2 (DEE2), a web-based repository of RNA-seq data in the form of gene-level and transcript-level expression counts. DEE2 contains over 470,000 RNA-seq data sets from nine species including E. coli, yeast, Arabidopsis, worm, fruit fly, zebrafish, rat, mouse and human. Base-space sequence data downloaded from NCBI Sequence Read Archive underwent quality control prior to transcriptome and genome mapping using open-source tools. Uniform data processing methods ensure consistency across experiments, facilitating fast and reproducible meta-analyses.<br>Conclusions: The web interface enables users to quickly identify data sets of interest through accession number and keyword searches. These data can also be accessed programmatically using a specifically designed R script. We demonstrate that DEE2 data is compatible with statistical packages such as edgeR or DESeq. Bulk data are also available for download. DEE2 can be found at http://dee2.io
**Corresponding Author:**	Mark Ziemann, PhD<br>Deakin University<br>Geelong, VIC AUSTRALIA
**Corresponding Author Secondary Information:**	
**Corresponding Author's Institution:**	Deakin University
**Corresponding Author's Secondary Institution:**	
**First Author:**	Mark Ziemann, PhD
**First Author Secondary Information:**	
**Order of Authors:**	Mark Ziemann, PhD
	Antony Kaspi, PhD
	Assam El-Osta, PhD
**Order of Authors Secondary Information:**	
**Additional Information:**	
**Question**	**Response**
Are you submitting this manuscript to a special series or article collection?	No
**Experimental design and statistics**	Yes

Powered by Editorial Manager® and ProduXion Manager® from Aries Systems Corporation

Full details of the experimental design and statistical methods used should be given in the Methods section, as detailed in our Minimum Standards Reporting Checklist. Information essential to interpreting the data presented should be made available in the figure legends.<br><br>Have you included all the information requested in your manuscript?	
**Resources**<br><br>A description of all resources used, including antibodies, cell lines, animals and software tools, with enough information to allow them to be uniquely identified, should be included in the Methods section. Authors are strongly encouraged to cite Research Resource Identifiers (RRIDs) for antibodies, model organisms and tools, where possible.<br><br>Have you included the information requested as detailed in our Minimum Standards Reporting Checklist?	Yes
**Availability of data and materials**<br><br>All datasets and code on which the conclusions of the paper rely must be either included in your submission or deposited in publicly available repositories (where available and ethically appropriate), referencing such data using a unique identifier in the references and in the "Availability of Data and Materials" section of your manuscript.<br><br>Have you have met the above requirement as detailed in our Minimum Standards Reporting Checklist?	Yes

Manuscript

Click here to download Manuscript Ziemann-DEE2-GigaSci_2018-11-10.pdf

Digital Expression Explorer 2: a repository of uniformly processed RNA sequencing data

Mark Ziemann[ab]*, Antony Kaspi[b], Assam El-Osta[bc]

[a] *Deakin University, Geelong, Australia, School of Life and Environmental Sciences*

[b] *Department of Diabetes, Monash University Central Clinical School, The Alfred Medical Research and Education Precinct, Melbourne, Vic, Australia*

[c] *Hong Kong Institute of Diabetes and Obesity, Prince of Wales Hospital, The Chinese University of Hong Kong, Hong Kong SAR*

*Corresponding author: Mark Ziemann

Deakin University, Geelong, Australia, School of Life and Environmental Sciences. 75 Pigdons Road, Waurn Ponds VIC 3216 Australia

Tel: +61 3 522 78965

Email: m.ziemann@deakin.edu.au

Author name	Email address	ORCID ID	Postal address
Mark Ziemann	m.ziemann@deakin.edu.au	0000-0002-7688-6974	a,b,c
Antony Kaspi	antony.kaspi@monash.edu	-	b,c
Assam El-Osta	sam.el-osta@monash.edu	0000-0001-7968-7375	b,c,d

ABSTRACT

Background: Since the advent of RNA-seq 10 years ago, it has become an indispensable tool in the study of gene regulation. Although RNA-seq has brought with it better transcript coverage and quantification, there remain considerable barriers-to-entry for the novice computational biologist to analyze these large data sets. There is a real need for a repository of uniformly processed RNA-seq data that is easy to use.

Findings: To address these obstacles, we developed Digital Expression Explorer 2 (DEE2), a web-based repository of RNA-seq data in the form of gene-level and transcript-level expression counts. DEE2 contains over 470,000 RNA-seq data sets from nine species including *E. coli*, yeast, Arabidopsis, worm, fruit fly, zebrafish, rat, mouse and human. Base-space sequence data downloaded from NCBI Sequence Read Archive underwent quality control prior to transcriptome and genome mapping using open-source tools. Uniform data processing methods ensure consistency across experiments, facilitating fast and reproducible meta-analyses.

Conclusions: The web interface enables users to quickly identify data sets of interest through accession number and keyword searches. These data can also be accessed programmatically using a specifically designed R script. We demonstrate that DEE2 data is compatible with statistical packages such as edgeR or DESeq. Bulk data are also available for download. DEE2 can be found at http://dee2.io

KEY WORDS

Gene expression, RNA-seq, transcriptome, data re-use

BACKGROUND

RNA-seq has become as a powerful method in transcriptomics, allowing highly accurate gene expression quantification [1]. As the cost of sequencing falls, RNA-seq data is becoming more ubiquitous in the scientific literature. It is standard practice in the field and compulsory requirement for journals to deposit these data to Gene Expression Omnibus (GEO) and Sequence Read Archive (SRA) [2,3] in the form of raw and processed files, with the aim of fostering greater reuse and transparency. In practice however, there are several hurdles which impede widespread reuse by biologists. Firstly, processing raw sequence data from SRA requires significant computational resources and command-line expertise. Secondly, the processed RNA-seq data hosted by GEO are prepared in assorted formats, that utilize various software tools and genome annotation sets, which complicates meta-analyses. Despite the value of these data to the scientific community and tremendous cost to generate them, RNA-seq data aggregation efforts have been largely limited to human and mouse [4,5] or are closed source / subscription services [6]. Expression Atlas is one of the most comprehensive repositories of processed expression microarray data with an informative graphical interface, but only a comparatively small number of RNA-seq datasets are currently included [7]. We sought to address this by developing Digital Expression Explorer 2 (DEE2), an open-access web-based repository of uniformly processed RNA-seq digital gene-level and transcript-level expression data for several major organisms that is compatible with many types of downstream analyses.

DATA PROCESSING

DEE2 consists of three parts: (i) a pipeline that downloads and process raw datasets from SRA. (ii) a data repository where processed files are collected, filtered, organized/stored and job queues are generated; and (iii) a web-server where users can search metadata and obtain datasets of interest. A schematic diagram of the organization of DEE2 is provided in Figure 1. Data processing nodes request SRA run accession numbers from the webserver and obtain raw data from SRA. Processed data is sent to

the webserver, validated and relayed to the DEE2 repository server. Repository server performs further validation checks, incorporates new datasets into the repository, collects corresponding metadata from SRAdbV2 [8] and queues outstanding jobs. The repository server then sends updated metadata and job queue on the webserver. End-users obtain data from the web-browser, command line or bulk dumps.

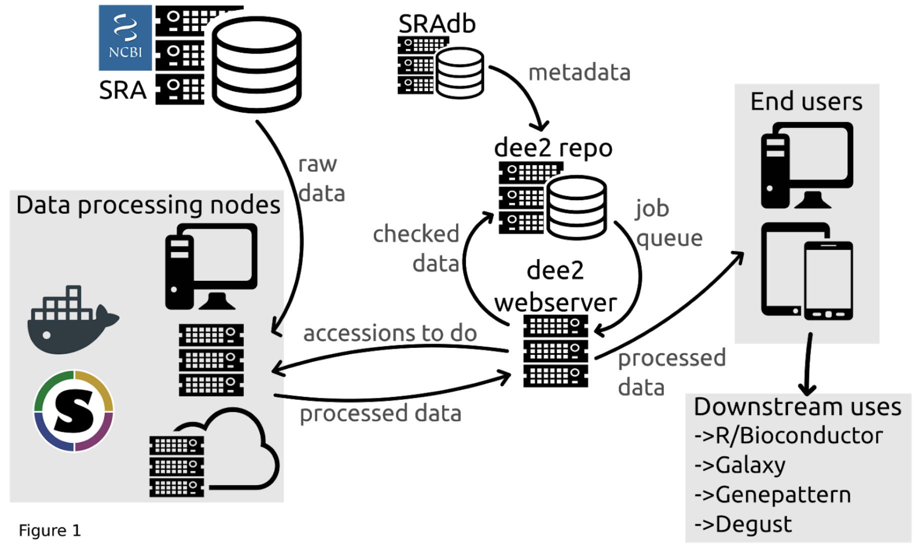

Figure 1. Overview of RNA-seq data processing, storage and provision.

PIPELINE FEATURES

The DEE2 pipeline utilizes containerization to enable rapid application deployment and guarantees analytical reproducibility across different computer systems. End users can run the Docker image [9] on their own hardware to process SRA datasets of interest as specified with a species name and SRA run accession. After completion of the processing, users will have immediate access to the outputs, and after validation by the DEE2 repository server, the datasets will be available publicly. In this way, power users obtain benefit by using an established analysis pipeline and simultaneously contribute to expanding the public resource. One concern with Docker images is that they cannot be run without administrator “root” permissions, for instance, by users on a high-performance computing system. To address this limitation, the image can be converted for use by Singularity [10] or UDocker [11] without root permissions. The steps involved in data processing are summarised in Figure 2.

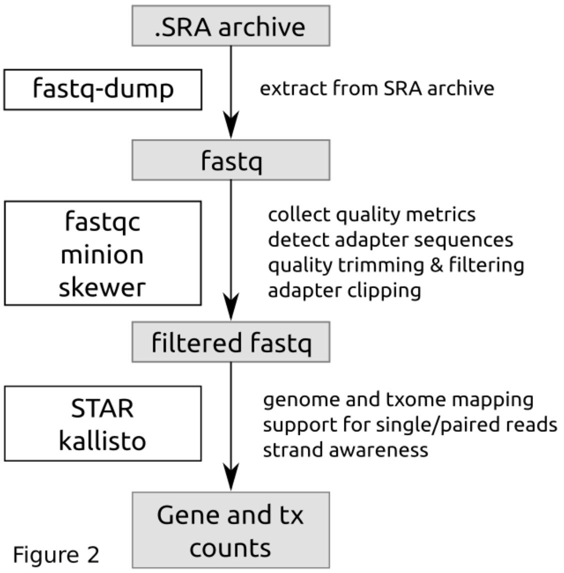

Figure 2

Figure 2. Overview of steps in the RNA-seq data processing pipeline.

The pipeline fetches the appropriate reference genome, annotation and cDNA sequence data from Ensembl [12]. Transcriptome sequencing datasets are downloaded from SRA using Aspera. The pipeline handles both single and paired end sequencing data with the exclusion of colorspace sequence data. A sample of 4000 reads is used to perform basic checks including read and quality string format using FastQC [13] prior to extraction of fastq files with a parallel implementation of fastq-dump [14]. Skewer is used to trim bases with phred quality less than 10 on the 3' ends [15]. Adapter sequences at the 3' end are detected using Minion, part of the Kraken package [16]. Adapter sequences are clipped using Skewer if the predicted adapter sequence is not present in the genome and exceeds a frequency of 2.5%. To handle non-reference 5' bases including UMIs, a sample of 10,000 reads undergo progressive clipping of 5' ends (5, 9, 13, 21 nt) followed by genomic mapping with STAR to determine the optimal number of bases to clip from the 5' end. STAR is then used to map all QC-passed reads to to the genome and generate gene-wise expression counts [17]. These counts are also used to diagnose whether the dataset is strand specific. This option is passed to Kallisto which maps reads to the transcriptome to generate transcript-wise expression values [18]. Gene and transcript counts along with analysis logs and QC metrics are zipped and transferred to the web server by sftp. The pipeline has the

added ability to process users own fastq files using the same pipeline, although the results remain private. The pipeline code is open source and available online [19].

DATA PROVIDED

Currently DEE2 hosts data from nine organisms selected as they are important model organisms and have large number of corresponding transcriptome datasets in SRA. Currently, there are over 470,000 RNA-seq data sets available, with each dataset corresponding to a specific SRA run number. Together the nine species included constitute 73.5% of all transcriptome datasets available from SRA[a]. DEE2 consists of over 4.5 trillion assigned sequence reads (Table 1). The data provided include gene-wise expression counts, transcript-wise counts, and QC metrics, provided as three separate matrices in tsv format. In addition, analysis logs for each dataset are available.

Table 1. Hosted gene expression data as of 9th November 2018.

Species	Projects	Experiments	Runs	Assigned reads (STAR)	Assigned reads (Kallisto)
A. thaliana	924	14514	23183	2.38E+11	2.43E+11
C. elegans	285	5491	7423	8.09E+10	7.32E+10
D. melanogaster	620	13850	18118	1.67E+11	1.79E+11
D. rerio	423	23979	25692	9.26E+10	9.15E+10
E. coli	176	1467	1617	1.25E+10	9.35E+09
H. sapiens	5787	151120	175049	1.79E+12	1.97E+12
M. musculus	5737	174576	208579	1.64E+12	1.85E+12
R. norvegicus	337	4144	5046	5.01E+10	5.37E+10
S. cerevisiae	440	10236	11366	7.40E+10	7.32E+10
Total	14729	399377	476073	4.14E+12	4.55E+12

QUALITY CONTROL AND DATA VALIDATION

Quality control is paramount for a resource such as this. A range of quality metrics are accessible and can be viewed on the search results page that includes mean base quality scores, number of reads, alignment rates and read assignment statistics. Detailed analysis logs are distributed alongside expression count matrices. To demonstrate the accuracy of the pipeline, we performed a simulation

study. Synthetic Illumina HiSeq RNA-seq data were generated from Ensembl transcripts and processed with the pipeline. The reads per million (RPM) values were compared between the simulated [20] and processed data and Spearman correlation coefficients (ρ) were calculated (Figure 3; Supplementary Table 1). We observed that analyses of simpler organisms were, in general, more accurate than for more complex transcriptomes of human and mouse. Overall there was only a modest improvement in accuracy in paired end over single end reads. Transcript quantification results were less accurate than gene level quantification. On the other hand, Kallisto transcript counts collapsed into their parent gene were more accurate than STAR gene counts (Figure 3; Supplementary Table 1), consistent with previous a previous report [21].

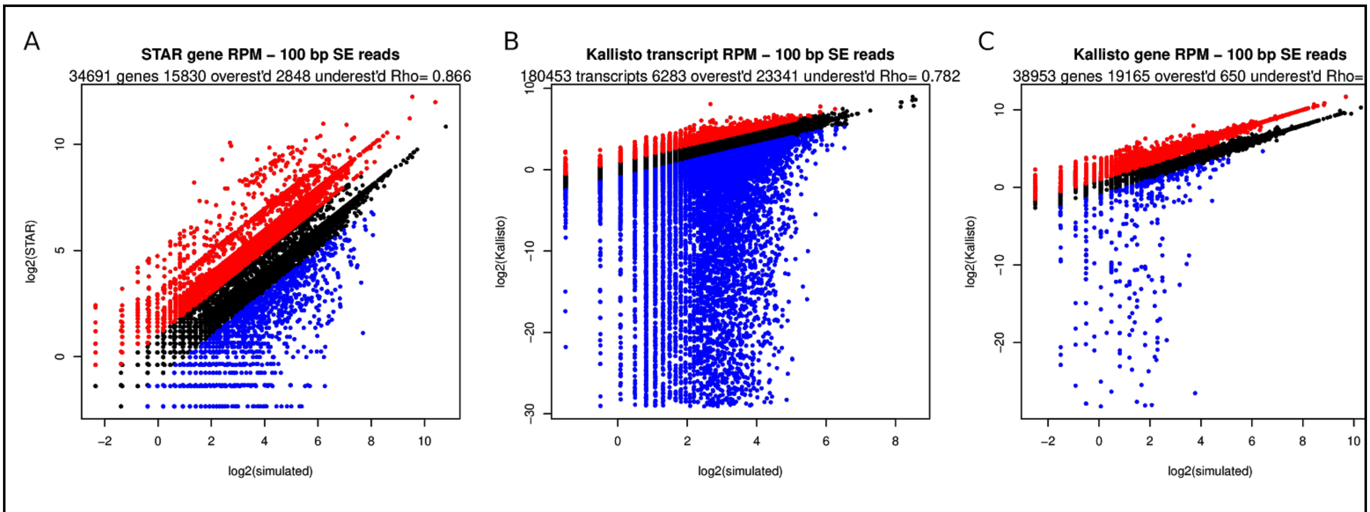

Figure 3. Comparison of simulated with DEE2 processed expression profiles. RNA-seq reads simulated with ART underwent mapping with the DEE2 pipeline generating gene level and transcript level expression counts. Count values were normalized for library size and plotted against the expected values. Points in red are those genes or transcripts observed with expression >2 fold higher than expected. Points in blue are those genes or transcripts observed with expression <2 fold lower than expected. (A) Gene level quantification of uniquely mapped reads using STAR. (B) Transcript level quantification using Kallisto. (C) Gene level quantification of Kallisto transcripts collapsed to parent gene.

In a separate validation exercise, we selected one experiment from each species and correlated DEE2-STAR counts with author-supplied gene expression counts. Despite differences in annotation sets and analytical pipelines, DEE2-derived count data correlated strongly with author supplied counts. Spearman ρ values were in the range of 0.95-0.99 (Figure 4A). After differential expression analysis with edgeR [22], we determined the correlation in differential expression between DEE2-STAR and

author supplied counts. Spearman ρ ranged between 0.55 and 0.95 (Figure 4B). Correlation was higher in contrasts that had greater sequencing depth and more replicates. Both validation exercises demonstrate the accuracy of DEE2 data.

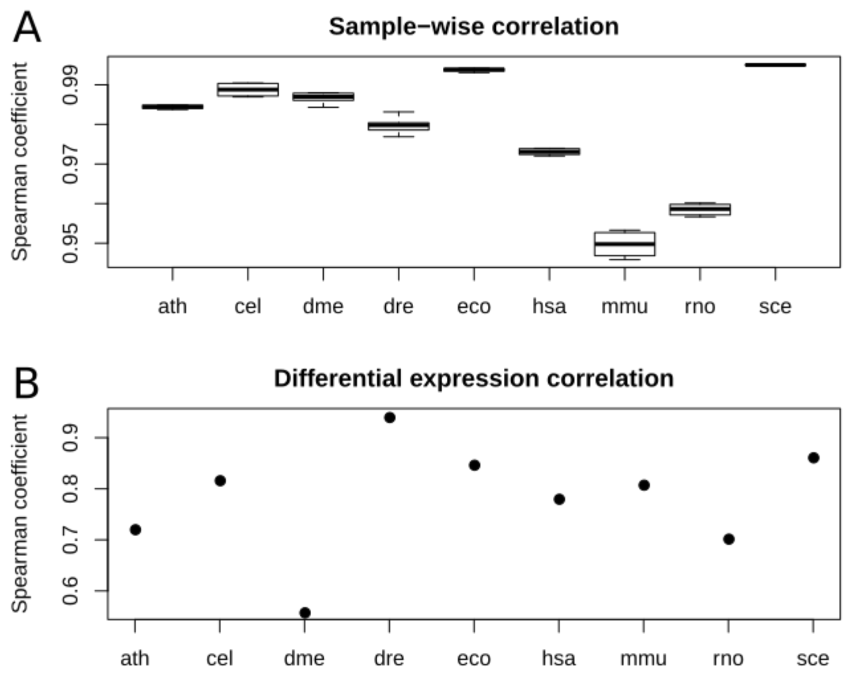

Figure 4. Comparison of DEE2 data with author uploaded gene-level count data. (A) Comparison of sample level counts. (B) Comparison of differential expression. Ath; *A. thaliana*, cel; *C. elegans*, dme; *D. melanogaster*, dre; *D. rerio*, eco; *E. ecoli*, hsa; *H. sapiens*, mmu; *M. musculus*, rno; *R. norvegicus*, sce; *S. cerevisiae*. GEO series accessions used in this study: GSE53078, GSE46344, GSE43180, GSE80768, GSE80251, GSE63776, GSE59970, GSE65715 and GSE76444.

RE-USE POTENTIAL

The financial cost of generating these raw datasets is substantial. A rough estimate of the cost to generate raw data included in DEE2 is ~$136 million USD[b]. In contrast, aggregation efforts like DEE2, with a modest budget, can add substantial value to these existing data by enabling straight-forward re-use. Another benefit of aggregation is that genome annotations are updated over time as compared to author-submitted data that remain static.

To enhance the re-use potential, we have designed a simple and easy to use website to access the data. Users select one of the nine species featured and provide either a keyword or accession number search to identify datasets of interest (Figure 5A). The results page shows a table of hits, together with their corresponding SRA accession numbers and keyword context (Figure 5B). The results

page also provides QC information which can be browsed by hovering the mouse over the QC summary field, so that users can be assured of dataset quality. Users then tick the box of every dataset they would like to download and by hitting the “Get Counts” button, the datasets are downloaded. The searching and retrieval steps for the example depicted in Figure 4, consisting of 107 human datasets took only 32 seconds. Figure 5C demonstrates how data is delivered to end users; as a zip archive containing tab-separated expression count and quality control information. The webserver is limited to fetching 500 datasets at a time. To enable easy access to large datasets we provide zip-packages of data for each project with ≥200 runs that have been fully processed by DEE2; there are 138 such packages currently (as of 9th November 2018).

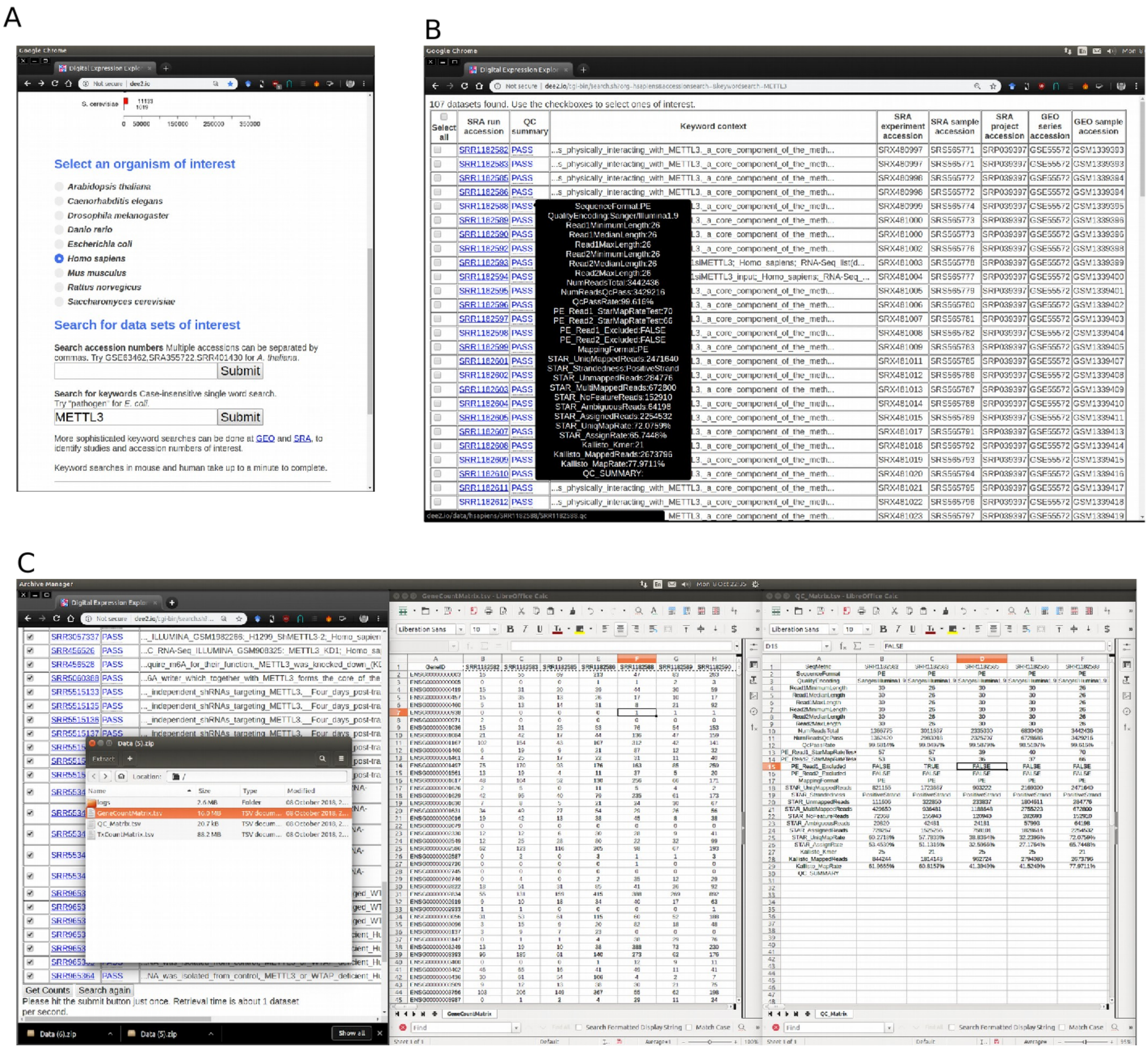

Figure 5. Example of the DEE2 web interface. (A) Users select the species of interest and supply either a keyword or accession number. (B) In this case, a search for METTL3, a gene name, yields 107 hits. Mouse pointer hovering over the QC summary field shows the key quality criteria of the processed datasets. (C) Users can select the datasets to download by ticking the boxes and hitting the "Get counts" button at the bottom of the page. The downloaded zip file (C left side) contains detailed processing logs of each run, along with a matrix of gene-wise counts (C middle), transcript- wise counts and QC information (C right side).

As R is the main language for downstream statistical analysis of RNA-seq data, we also provide a suite of R functions to obtain DEE2 data. In the example shown in Box 1, datasets belonging to an experiment with GEO series GSE33569 are obtained.

```
#obtain the source code
> source("https://raw.githubusercontent.com/markziemann/dee2/master/getDEE2.R")
#obtain DEE2 metadata
> mdat<-getDee2Metadata("celegans")
trying URL 'http://dee2.io/metadata/celegans_metadata.tsv.cut'
Content type 'text/tab-separated-values' length 549206 bytes (536 KB)
==================================================
downloaded 536 KB

#Browse metadata for GEO series GSE33569
> mdat[which(mdat$GSE_accession %in% "GSE33569"),]
     SRR_accession QC_summary SRX_accession SRS_accession SRP_accession
2127     SRR363796       PASS     SRX105188     SRS270025     SRP009256
4608     SRR363797       PASS     SRX105189     SRS270026     SRP009256
5976     SRR363798       PASS     SRX105190     SRS270027     SRP009256
8072     SRR363799       PASS     SRX105191     SRS270028     SRP009256
     GSE_accession GSM_accession
2127      GSE33569     GSM829554
4608      GSE33569     GSM829555
5976      GSE33569     GSM829556
8072      GSE33569     GSM829557

> mdat1<-mdat[which(mdat$GSE_accession %in% "GSE33569"),]
> SRRlist<-as.vector(mdat1$SRR_accession)
> SRRlist
[1] "SRR363796" "SRR363797" "SRR363798" "SRR363799"

> x<-getDEE2("celegans",SRRlist)

trying URL
'http://dee2.io/cgi-bin/request.sh?org=celegans&x=SRR363796&x=SRR363797&x=SRR363798&x=SRR363799'
downloaded 479 KB

> names(x)
[1] "GeneCounts" "TxCounts"   "QcMx"       "absent"
> head(x$GeneCounts)
               SRR363796 SRR363797 SRR363798 SRR363799
WBGene00197333         0         0         0         0
WBGene00198386         0         0         0         0
WBGene00015153         4        16         6         4
WBGene00002061        44       100       217        77

> head(x$TxCounts)
           SRR363796 SRR363797 SRR363798 SRR363799
Y110A7A.10        11        23        48        45
F27C8.1            0         0         0         0
F07C3.7            0         2         0        21
F52H2.2a           0         0         7         0

> head(x$QcMx)
                                   SE                SE.1                SE.2
SequenceFormat     Sanger/Illumina1.9 Sanger/Illumina1.9 Sanger/Illumina1.9
QualityEncoding                    36                 36                 36
Read1MinimumLength                 36                 36                 36
Read1MedianLength                  36                 36                 36

                                 SE.3
SequenceFormat     Sanger/Illumina1.9
QualityEncoding                    36
Read1MinimumLength                 36
Read1MedianLength                  36
```

Box 1. An example of obtaining gene and transcript expression datasets using the R functions (GEO series: GSE33569).

For power users, bulk data dumps are available and will be of use to researchers wishing to do wholesale meta-analyses, as three studies already have [23-25]. Irrespective of the method of acquisition, DEE2 data are compatible with many different downstream applications including R/Bioconductor [26,27], Degust [28] and Galaxy [29].

CONCLUSION

DEE2 provides a unique framework and user-friendly resource for processed RNA-seq data that alleviates many of the bottlenecks researchers currently face with analysis of public RNA-seq data. Our testing shows DEE2 and Degust enable analysis of public RNA-seq data on mobile devices such as smartphones. Bulk data provided by DEE2 is a useful starting-point for researchers performing meta-analyses of RNA-seq data.

AVAILABILITY OF SOURCE CODE AND REQUIREMENTS

- *Project name: Digital Expression Explorer 2*
- *Project home page: http://dee2.io*
- *Operating systems (dataset): Platform independent*
- *Operating systems (pipeline): Unix and MacOS*
- *License: GNU GPL v3*
- *Any restrictions to use by non-academics: none*

AVAILABILITY OF SUPPORTING DATA

- *Dataset access: http://dee2.io*
- *Bulk data access: https://datbase.org/dee2/bulk*
- *Source code: https://github.com/markziemann/dee2*
- *Pipeline container: https://hub.docker.com/r/mziemann/tallyup/*

DECLARATIONS

Abbreviations

DEE2; Digital Expression Explorer 2, GEO; Gene Expression Omnibus, SRA; Sequence Read Archive, SRAdb; An R Package to Query the Sequence Read Archive, QC; quality control.

Competing interests

The authors declare that they have no competing interests.

Funding

AE-O is a Senior Research Fellow supported by NHMRC. AE-O receives funding from the National Health and Medical Research Council – Natural Science Foundation of China (NHMRC-NSFC International Joint Call APP1113188).

Authors' contributions

MZ and AE-O conceived and designed the study. MZ and AK wrote the computer code. MZ coordinated data processing and drafted the manuscript. All authors read, revised and approved the final manuscript.

Acknowledgments

This research was made possible by use of the Multi-modal Australian ScienceS Imaging and Visualisation Environment (MASSIVE) and Nectar Research Cloud, both supported by the Australian National Collaborative Research Infrastructure Strategy (NCRIS). This work was supported by Deakin eResearch and Monash eResearch Centres. We thank Dr Ross Lazarus and Dr Haloom Rafehi for bioinformatics expertise, advice and helpful discussions. We thank Julian Vreugdenburg for technical support. We thank the many users that have provided feedback on earlier versions of DEE2.

ENDNOTES

[a] These 9 species represent 822,819 of the 1,119,784 RNA experiments present in SRA as at 29th Oct 2018.

[b] Estimated cost of generating 4.55 trillion 100 bp SE reads from 399,377 experiments. Cornell University Institute of Biotechnology advertises HiSeq2500 at USD $18,407 for 8 lanes 100bp SE [30]. Illumina HiSeq2500 v4 spec sheet estimates 1.5 billion reads per 8 lane flow cell [31]. That gives USD $12.27 per million reads. The cost of sequencing is USD $55.8 million. Library prep costs approximately USD $200 per sample which is in the range advertised at Cornell multiplied by 399,377 experiments equates to USD $79.9 million for library construction. Grand total of USD $135.7 million.

REFERENCES

1. Nagalakshmi U, Wang Z, Waern K, Shou C, Raha D, Gerstein M, Snyder M. The transcriptional landscape of the yeast genome defined by RNA sequencing. Science. 2008;320:1344-9.
2. Barrett T, Wilhite SE, Ledoux P, Evangelista C, Kim IF, Tomashevsky M, Marshall KA, Phillippy KH, Sherman PM, Holko M, Yefanov A, Lee H, Zhang N, Robertson CL, Serova N, Davis S, Soboleva A. NCBI GEO: archive for functional genomics data sets--update. Nucleic Acids Res. 2013;41:D991-5.
3. Kodama Y, Shumway M, Leinonen R; International Nucleotide Sequence Database Collaboration. The Sequence Read Archive: explosive growth of sequencing data. Nucleic Acids Res. 2012;40:D54-6.
4. Collado-Torres L, Nellore A, Kammers K, Ellis SE, Taub MA, Hansen KD, Jaffe AE, Langmead B, Leek JT. Reproducible RNA-seq analysis using recount2. Nat Biotechnol. 2017;35:319-21.
5. Lachmann A, Torre D, Keenan AB, Jagodnik KM, Lee HJ, Wang L, Silverstein MC, Ma'ayan A. Massive mining of publicly available RNA-seq data from human and mouse. Nat Commun. 2018;9:1366.

6. Hruz T, Laule O, Szabo G, Wessendorp F, Bleuler S, Oertle L, Widmayer P, Gruissem W, Zimmermann P. Genevestigator v3: a reference expression database for the meta-analysis of transcriptomes. Adv Bioinformatics. 2008;2008:420747.
7. Papatheodorou I, Fonseca NA, Keays M, Tang YA, Barrera E, Bazant W, Burke M, Füllgrabe A, Fuentes AM, George N, Huerta L, Koskinen S, Mohammed S, Geniza M, Preece J, Jaiswal P, Jarnuczak AF, Huber W, Stegle O, Vizcaino JA, Brazma A, Petryszak R. Expression Atlas: gene and protein expression across multiple studies and organisms. Nucleic Acids Res. 2018;46:D246-D251.
8. Davis S. The SRAdbV2 Package. 2018. https://github.com/seandavi/SRAdbV2. Accessed 16 Oct 2018
9. Ziemann M. Tally-up: Bulk reprocessing of RNA-seq data. 2018. https://hub.docker.com/r/mziemann/tallyup. Accessed 16 Oct 2018.
10. Kurtzer GM, Sochat V, Bauer MW. Singularity: Scientific containers for mobility of compute. PLoS One. 2017;12:e0177459.
11. Gomes J, Bagnaschi E, Campos I, David M, Alves L, Martins J, Pina J, López-García A, Orviz P. Enabling rootless Linux Containers in multi-user environments: The udocker tool. Comput Phys Commun. 2018;232:84-97.
12. Zerbino DR, Achuthan P, Akanni W, Amode MR, Barrell D, Bhai J, Billis K, Cummins C, Gall A, Girón CG, Gil L, Gordon L, Haggerty L, Haskell E, Hourlier T, Izuogu OG, Janacek SH, Juettemann T, To JK, Laird MR, Lavidas I, Liu Z, Loveland JE, Maurel T, McLaren W, Moore B, Mudge J, Murphy DN, Newman V, Nuhn M, Ogeh D, Ong CK, Parker A, Patricio M, Riat HS, Schuilenburg H, Sheppard D, Sparrow H, Taylor K, Thormann A, Vullo A, Walts B, Zadissa A, Frankish A, Hunt SE, Kostadima M, Langridge N, Martin FJ, Muffato M, Perry E, Ruffier M, Staines DM, Trevanion SJ, Aken BL, Cunningham F, Yates A, Flicek P. Ensembl 2018. Nucleic Acids Res. 2018;46:D754-D761.
13. Andrews, S. FastQC: a quality control tool for high throughput sequence data. 2010. http://www.bioinformatics.babraham.ac.uk/projects/fastqc. Accessed 5th March 2018.

14. Valeris, R. Parallel-fastq-dump. 2016. https://github.com/rvalieris/parallel-fastq-dump. Accessed 5th March 2018.

15. Jiang H, Lei R, Ding SW, Zhu S. Skewer: a fast and accurate adapter trimmer for next-generation sequencing paired-end reads. BMC Bioinformatics. 2014;15:182.

16. Davis MP, van Dongen S, Abreu-Goodger C, Bartonicek N, Enright AJ. Kraken: a set of tools for quality control and analysis of high-throughput sequence data. Methods. 2013;63:41-9.

17. Dobin A, Davis CA, Schlesinger F, Drenkow J, Zaleski C, Jha S, Batut P, Chaisson M, Gingeras TR. STAR: ultrafast universal RNA-seq aligner. Bioinformatics. 2013;29:15-21.

18. Bray NL, Pimentel H, Melsted P, Pachter L. Near-optimal probabilistic RNA-seq quantification. Nat Biotechnol. 2016;34:525-7.

19. Ziemann, M. Digital Expression Explorer 2 (DEE2): a repository of uniformly processed RNA-seq data. 2018. https://github.com/markziemann/dee2. Accessed 16 Oct 2018.

20. Huang W, Li L, Myers JR, Marth GT. ART: a next-generation sequencing read simulator. Bioinformatics. 2012;28:593-4.

21. Soneson C, Love MI, Robinson MD. Differential analyses for RNA-seq: transcript-level estimates improve gene-level inferences. Version 2. F1000Res. 2015;4:1521.

22. Robinson MD, McCarthy DJ, Smyth GK. edgeR: a Bioconductor package for differential expression analysis of digital gene expression data. Bioinformatics. 2010;26:139-40.

23. Rau A, Maugis-Rabusseau C. Transformation and model choice for RNA-seq co-expression analysis. Brief Bioinform. 2017;pii:bbw128.

24. Espinar L, Schikora Tamarit MÀ, Domingo J, Carey LB. Promoter architecture determines cotranslational regulation of mRNA. Genome Res. 2018;28:509-18.

25. Godichon-Baggioni A, Maugis-Rabusseau C, Rau A. Clustering transformed compositional data using K-means, with applications in gene expression and bicycle sharing system data. J Appl Stat. 2018;3:1-9.

26. Ihaka R, Gentleman R. R: A Language for Data Analysis and Graphics. J Comput Graph Stat. 1996;3:299-314.

27. Huber W, Carey VJ, Gentleman R, Anders S, Carlson M, Carvalho BS, Bravo HC, Davis S, Gatto L, Girke T, Gottardo R, Hahne F, Hansen KD, Irizarry RA, Lawrence M, Love MI, MacDonald J, Obenchain V, Oleś AK, Pagès H, Reyes A, Shannon P, Smyth GK, Tenenbaum D, Waldron L, Morgan M. Orchestrating high-throughput genomic analysis with Bioconductor. Nat Methods. 2015;12:115-21.

28. Powell D. Degust: RNA-seq exploration, analysis and visualisation. 2013. http://degust.erc.monash.edu. Accessed 6 Apr 2018.

29. Afgan E, Baker D, van den Beek M, Blankenberg D, Bouvier D, Čech M, Chilton J, Clements D, Coraor N, Eberhard C, Grüning B, Guerler A, Hillman-Jackson J, Von Kuster G, Rasche E, Soranzo N, Turaga N, Taylor J, Nekrutenko A, Goecks J. The Galaxy platform for accessible, reproducible and collaborative biomedical analyses: 2016 update. Nucleic Acids Res. 2016;44:W3-W10.

30. Cornell University Institute of Biotechnology Illumina Sequencing Price List. http://www.biotech.cornell.edu/brc/genomics/services/price-list Accessed 24th Oct 2018.

31. Illumina Inc. System Specification Sheet for the HiSeq 2500 System. 2015. https://www.illumina.com/documents/products/datasheets/datasheet_hiseq2500.pdf Accessed 24th Oct 2018.

Supplementary Table 1. Spearman correlation coefficients (ρ) for simulated RNA-seq read sets after processing with the DEE2 pipeline.

Species	Seq format	STAR (gene)	Kallisto (transcript)	Kallisto (gene)
A. thaliana	50 bp SE	0.958	0.746	0.994
	100 bp SE	0.957	0.733	0.997
	50 bp PE	0.946	0.604	0.992
	100 bp PE	0.948	0.682	0.997
C. elegans	50 bp SE	0.939	0.988	0.979
	100 bp SE	0.946	0.759	0.985
	50 bp PE	0.937	0.609	0.965
	100 bp PE	0.940	0.691	0.981
D. melanogaster	50 bp SE	0.913	0.783	0.994
	100 bp SE	0.912	0.790	0.997
	50 bp PE	0.904	0.652	0.992
	100 bp PE	0.905	0.706	0.997
D. rerio	50 bp SE	0.924	0.838	0.993
	100 bp SE	0.947	0.901	0.996
	50 bp PE	0.932	0.786	0.989
	100 bp PE	0.952	0.877	0.994
E. coli	50 bp SE	0.980	0.985	0.985
	100 bp SE	0.981	0.998	0.998
	50 bp PE	0.976	0.980	0.980
	100 bp PE	0.982	0.999	0.999
H. sapiens	50 bp SE	0.863	0.785	0.957
	100 bp SE	0.866	0.782	0.907
	50 bp PE	0.895	0.541	0.967
	100 bp PE	0.926	0.752	0.988
M. musculus	50 bp SE	0.923	0.863	0.967
	100 bp SE	0.937	0.863	0.991
	50 bp PE	0.728	0.708	0.969
	100 bp PE	0.935	0.827	0.990
R. norvegicus	50 bp SE	0.881	0.877	0.989
	100 bp SE	0.895	0.943	0.920
	50 bp PE	0.874	0.895	0.983
	100 bp PE	0.900	0.916	0.991
S. cerevisiae	50 bp SE	0.920	0.876	0.876
	100 bp SE	0.929	0.983	0.983
	50 bp PE	0.460	0.918	0.918
	100 bp PE	0.929	0.983	0.983
