# Supplementary material for: Digital expression explorer 2: a repository of uniformly processed RNA sequencing data
Source: Gigascience. 2019 Apr 3;8(4):giz022. doi: 10.1093/gigascience/giz022 (PMC6446219; doi:10.1093/gigascience/giz022)
Supplement: GIGA-D-18-00444_Revision-1.pdf [file giz022_giga-d-18-00444_revision-1.pdf]

GigaScience

Digital Expression Explorer 2: a repository of uniformly processed RNA sequencing data

--Manuscript Draft--

**Manuscript Number:**	GIGA-D-18-00444R1
**Full Title:**	Digital Expression Explorer 2: a repository of uniformly processed RNA sequencing data
**Article Type:**	Data Note
**Funding Information:**	National Health and Medical Research Council (APP1113188) — Prof Assam El-Osta
**Abstract:**	Background: RNA-seq is an indispensable tool in the study of gene regulation. While the technology has brought with it better transcript coverage and quantification, there remain considerable barriers-to-entry for the computational biologist to analyze large data sets. There is a real need for a repository of uniformly processed RNA-seq data that is easy to use.<br>Findings: To address these obstacles, we developed Digital Expression Explorer 2 (DEE2), a web-based repository of RNA-seq data in the form of gene-level and transcript-level expression counts. DEE2 contains over 5.3 trillion assigned reads from 580,000 RNA-seq data sets including species E. coli, yeast, Arabidopsis, worm, fruit fly, zebrafish, rat, mouse and human. Base-space sequence data downloaded from NCBI Sequence Read Archive underwent quality control prior to transcriptome and genome mapping using open-source tools. Uniform data processing methods ensure consistency across experiments, facilitating fast and reproducible meta-analyses.<br>Conclusions: The web interface allows users to quickly identify data sets of interest using accession number and keyword searches. The data can also be accessed programmatically using a specifically designed R package. We demonstrate that DEE2 data is compatible with statistical packages such as edgeR or DESeq. Bulk data are also available for download. DEE2 can be found at http://dee2.io
**Corresponding Author:**	Mark Ziemann, PhD<br>Deakin University<br>Geelong, VIC AUSTRALIA
**Corresponding Author Secondary Information:**	
**Corresponding Author's Institution:**	Deakin University
**Corresponding Author's Secondary Institution:**	
**First Author:**	Mark Ziemann, PhD
**First Author Secondary Information:**	
**Order of Authors:**	Mark Ziemann, PhD
	Antony Kaspi, PhD
	Assam El-Osta, PhD
**Order of Authors Secondary Information:**	
**Response to Reviewers:**	Dear Dr Edmunds,<br><br>We thank both reviewers for their thorough review and highly constructive suggestions which have improved this body of work markedly.<br><br>In the Personal Cover docx document we uploaded, we have addressed each of the reviewers' points (#1 to #37). To make it clear what changes have been made to the manuscript we have provided a copy of the manuscript with changes annotated with numbered comments (#1 to #37) in addition to a "clean" version.

Powered by Editorial Manager® and ProduXion Manager® from Aries Systems Corporation

	(the response to reviewers' comments document is 15 pages and contains several tables and graphs, so we uploaded it as a separate docx document)<br><br>Also in accordance with editorial guidelines, we have added SciCrunch RRIDs for essential DEE2 resources:<br>1) SCR_016931 Docker Image<br>2) SCR_016930 Source Code<br>3) SCR_016929 Project Homepage<br><br>We certainly hope that the revised manuscript is suitable for publication in GigaScience.<br><br>Sincerely,<br><br>Mark Ziemann, PhD<br>Deakin University
**Additional Information:**	
**Question**	**Response**
Are you submitting this manuscript to a special series or article collection?	No
**Experimental design and statistics**<br><br>Full details of the experimental design and statistical methods used should be given in the Methods section, as detailed in our Minimum Standards Reporting Checklist. Information essential to interpreting the data presented should be made available in the figure legends.<br><br>Have you included all the information requested in your manuscript?	Yes
**Resources**<br><br>A description of all resources used, including antibodies, cell lines, animals and software tools, with enough information to allow them to be uniquely identified, should be included in the Methods section. Authors are strongly encouraged to cite Research Resource Identifiers (RRIDs) for antibodies, model organisms and tools, where possible.<br><br>Have you included the information requested as detailed in our Minimum Standards Reporting Checklist?	Yes

Powered by Editorial Manager® and ProduXion Manager® from Aries Systems Corporation

**Availability of data and materials**<br><br>All datasets and code on which the conclusions of the paper rely must be either included in your submission or deposited in publicly available repositories (where available and ethically appropriate), referencing such data using a unique identifier in the references and in the “Availability of Data and Materials” section of your manuscript.<br><br>Have you have met the above requirement as detailed in our Minimum Standards Reporting Checklist?	Yes

Powered by Editorial Manager® and ProduXion Manager® from Aries Systems Corporation

Manuscript

Click here to view linked References

Click here to access/download;Manuscript;DEE2 manuscript GigaScience Revised - clean.docx

Digital Expression Explorer 2: a repository of uniformly processed RNA sequencing data

Mark Ziemann[ab]*, Antony Kaspi[b], Assam El-Osta[bc]

[a] *Deakin University, Geelong, Australia, School of Life and Environmental Sciences*

[b] *Department of Diabetes, Monash University Central Clinical School, The Alfred Medical Research and Education Precinct, Melbourne, Vic, Australia*

[c] *Hong Kong Institute of Diabetes and Obesity, Prince of Wales Hospital, The Chinese University of Hong Kong, Hong Kong SAR*

*Corresponding author: Mark Ziemann

Deakin University, Geelong, Australia, School of Life and Environmental Sciences. 75 Pigdons Road, Waurn Ponds VIC 3216 Australia

Tel: +61 3 522 78965

Email: m.ziemann@deakin.edu.au

Author name	Email address	ORCID ID	Postal address
Mark Ziemann	m.ziemann@deakin.edu.au	0000-0002-7688-6974	a,b
Antony Kaspi	akaspi@gmail.com	-	b
Assam El-Osta	sam.el-osta@monash.edu	0000-0001-7968-7375	b,c

ABSTRACT

Background: RNA-seq is an indispensable tool in the study of gene regulation. While the technology has brought with it better transcript coverage and quantification, there remain considerable barriers-to-entry for the computational biologist to analyze large data sets. There is a real need for a repository of uniformly processed RNA-seq data that is easy to use.

Findings: To address these obstacles, we developed Digital Expression Explorer 2 (DEE2), a web-based repository of RNA-seq data in the form of gene-level and transcript-level expression counts. DEE2 contains over 5.3 trillion assigned reads from 580,000 RNA-seq data sets including species *E. coli*, yeast, Arabidopsis, worm, fruit fly, zebrafish, rat, mouse and human. Base-space sequence data downloaded from NCBI Sequence Read Archive underwent quality control prior to transcriptome and genome mapping using open-source tools. Uniform data processing methods ensure consistency across experiments, facilitating fast and reproducible meta-analyses.

Conclusions: The web interface allows users to quickly identify data sets of interest using accession number and keyword searches. The data can also be accessed programmatically using a specifically designed R package. We demonstrate that DEE2 data is compatible with statistical packages such as edgeR or DESeq. Bulk data are also available for download. DEE2 can be found at http://dee2.io

KEY WORDS

Gene expression, RNA-seq, transcriptome, data re-use

BACKGROUND

Since it’s first description ten years ago, RNA-seq has become a powerful method in transcriptomics, allowing highly accurate gene expression quantification [1]. As the cost of sequencing falls, RNA-seq data is becoming more ubiquitous in the scientific literature. It is standard practice in the field and compulsory requirement for journals to deposit these data to Gene Expression Omnibus (GEO) and Sequence Read Archive (SRA) [2,3] in the form of raw and processed files, with the aim of fostering greater reuse and transparency. In practice however, there are several hurdles which impede widespread reuse by biologists. Firstly, processing raw sequence data from SRA requires significant computational resources and command-line expertise. Secondly, the processed RNA-seq data hosted by GEO are prepared in assorted formats, that utilize various software tools and genome annotation sets, which complicates meta-analyses. Despite the value of these data to the scientific community and tremendous cost to generate them, RNA-seq data aggregation efforts have been largely limited to human and mouse [4,5] or are closed source / subscription services [6]. BgeeDB provides array and sequencing based expression data on many animal species with a particular focus on high quality measurements of baseline samples at different life stages (excluding disease, treatments or genetic perturbations) [7]. Expression Atlas is one of the most comprehensive repositories of processed expression microarray data with an informative graphical interface, but only a comparatively small number of RNA-seq datasets are currently included [8]. In an effort to boost reuse of public transcriptome data, we developed Digital Expression Explorer 2 (DEE2), an open-access web-based repository of uniformly processed RNA-seq digital gene-level and transcript-level expression data for several major organisms that is compatible with many types of downstream analyses.

DATA PROCESSING

DEE2 consists of three parts: (i) a pipeline that downloads and process raw datasets from SRA. (ii) a data repository where processed files are collected, filtered, organized/stored and job queues are generated; and (iii) a web-server where users can search metadata and obtain datasets of interest. A schematic diagram of the organization of DEE2 is provided in Figure 1. Data processing nodes request SRA run accession numbers from the webserver and obtain raw data from SRA. Processed data is sent to the webserver, validated and relayed to the DEE2 repository server. Repository server performs further validation checks, incorporates new datasets into the repository, collects corresponding metadata from SRAdbV2 [9] and queues outstanding jobs. The repository server then sends updated metadata and job queue on the webserver. End-users obtain data from the web-browser, command line or bulk dumps.

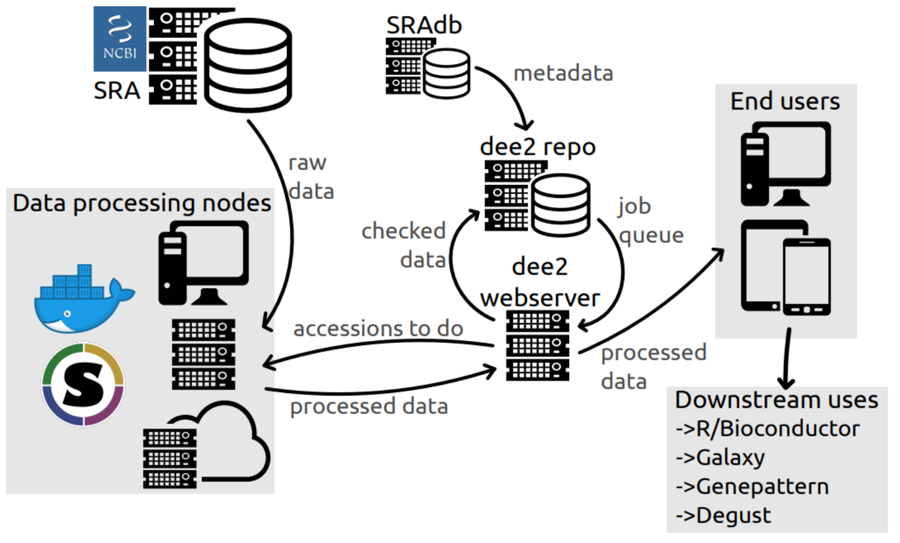

Figure 1. Overview of RNA-seq data processing, storage and provision.

PIPELINE FEATURES

The DEE2 pipeline utilizes containerization to enable rapid application deployment and guarantees analytical reproducibility across different computer systems. End users can run the Docker image [10] on their own hardware to process SRA datasets of interest as specified with a species name and SRA run accession. After completion of the processing, users will have immediate access to the outputs, and after validation by the DEE2 repository server, the

datasets will be available publicly. In this way, power users obtain benefit by using an established analysis pipeline and simultaneously contribute to expanding the public resource. One concern with Docker images is that they cannot be run without administrator “root” permissions, for instance, by users of a shared high-performance computing system. To address this limitation, the image can be converted for use by Singularity [11] or UDocker [12] without root permissions.

The steps involved in data processing are summarised in Figure 2. The pipeline fetches the appropriate reference genome, annotation and cDNA sequence data from Ensembl (Aug 2017 version) [13]. Transcriptome sequencing datasets are downloaded from SRA using Aspera. The pipeline handles both single and paired end sequencing data with the exclusion of colorspace sequence data. A sample of 4000 reads is used to perform basic checks including read and quality string format using FastQC [14] prior to extraction of fastq files with a parallel implementation of fastq-dump [15]. Skewer [16] is used to trim bases with phred quality less than 10 on the 3’ ends and discards reads shorter than 18 nt. Adapter sequences at the 3’ end are detected using Minion, part of the Kraken package [17]. Adapter sequences are clipped using Skewer if the predicted adapter sequence is not present in the genome and exceeds a frequency of 2.5%. To handle non-reference 5’ bases including unique molecular identifiers, a sample of 10,000 reads undergo progressive clipping of 5’ ends (4, 8, 12, 20 nt) followed by genomic mapping with STAR to determine the optimal number of bases to clip from the 5’ end, as determined by the proportion of uniquely mapped reads. STAR [18] is then used to map all reads that pass quality control (QC) to the genome and generate gene-wise expression counts with the “--quantMode GeneCounts” (no alignment files are generated). STAR output is also used to diagnose whether the dataset is strand specific. In order to be classified as strand specific, there needs to be a 5:1 strand bias in assigned reads according to STAR. This option is passed to Kallisto which maps reads to the transcriptome to generate estimated transcript counts [19]. Gene and transcript counts along with analysis logs and QC metrics are zipped and

transferred to the web server by sftp. The pipeline has the added ability to process users own fastq files using the same pipeline, although the results remain private. The pipeline code is open source and available online [20]. Software versions and parameters used in the pipeline are provided in Supplementary Table 1.

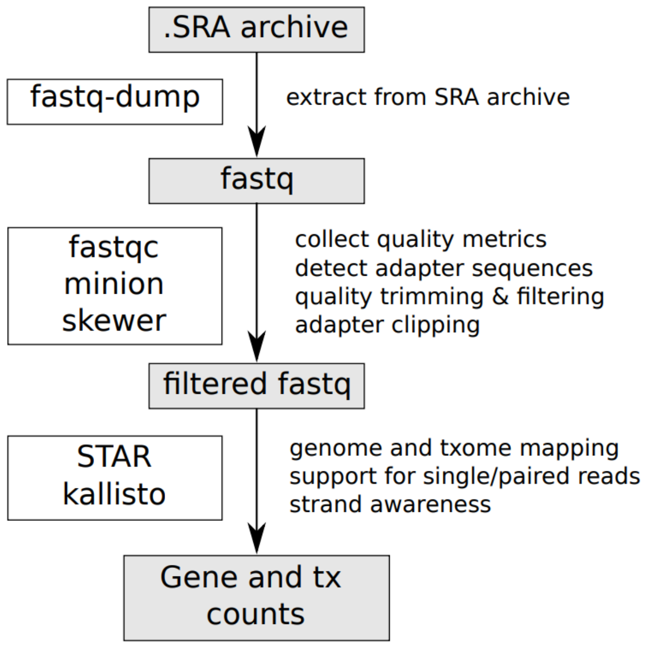

Figure 2. Overview of steps in the RNA-seq data processing pipeline.

DATA PROVIDED

Currently DEE2 hosts data from nine organisms selected as they are important model organisms and have large number of corresponding transcriptome datasets in SRA. Currently, there are over 580,000 RNA-seq data sets available, with each dataset corresponding to a specific SRA run number. Together the nine species included constitute 73.5% of all transcriptome datasets available from SRA[a]. DEE2 consists of over 5.3 trillion assigned sequence reads (Table 1). The data provided include gene-wise expression counts, transcript-wise estimated counts, gene information, transcript information, summary metadata, full metadata and QC metrics, provided as seven separate tables in tsv format. The gene information table contains the gene accession number, corresponding gene symbol and gene length as calculated by GTFtools v0.6.5 [21] The transcript information file contains transcript-

parent gene relationships, gene symbol and transcript length. Gene and transcript length information will allow straightforward normalisation of expression by contig length. The full metadata table contains all corresponding metadata from SRAdbV2, while the summary metadata contains only corresponding SRA accession numbers and experiment title. Moreover, analysis logs for each dataset are provided. Classification of datasets by QC metrics is discussed below.

Table 1. Hosted gene expression data as of 11th January 2019.

Species	Projects	Experiments	Runs	QC classification pass/warn/fail	Assigned reads (STAR)	Assigned reads (Kallisto)
A. thaliana	986	17095	26061	5602/15122/5337	2.87E+11	2.92E+11
C. elegans	339	5759	7722	1647/2446/3629	8.71E+10	7.88E+10
D. melanogaster	678	14401	18713	4410/7471/6832	1.75E+11	1.87E+11
D. rerio	457	26246	28100	1084/5826/21190	1.11E+11	6.20E+10
E. coli	180	1488	1638	355/376/907	1.26E+10	9.40E+09
H. sapiens	6768	197836	229634	42225/77254/110155	2.27E+12	2.51E+12
M. musculus	7078	204850	252058	23840/85874/142344	1.84E+12	2.08E+12
R. norvegicus	349	4965	5799	426/2651/2900	5.95E+10	6.42E+10
S. cerevisiae	442	10239	11369	3025/2783/5561	7.41E+10	7.32E+10
Total	17277	482879	581094	82614/199803/298855	4.92E+12	5.35E+12

QUALITY CONTROL METRICS

QC is paramount for a resource such as this. A range of quality metrics are accessible and can be viewed on the search results page that includes mean base quality scores, number of reads, alignment rates and read assignment statistics. Detailed analysis logs are distributed alongside expression data. Summary statistics for human datasets are provided in Figure 3. There are roughly equal numbers of runs with single and paired sequencing (Figure 3A). The overwhelming majority of datasets are encoded in Illumina 1.9 format (also known as Sanger), and a small number of datasets with Illumina 1.5 quality encoding (Figure 3B). Median read

length is 75 bp and mode is 50 bp (Figure 3C). Most (71.3%) of datasets had ≥ 95% of reads pass QC filtering (Figure 3D). Median number of reads passing QC filtering was 4.6 million (Figure 3E). Median proportion of STAR unique mapping was 82% (Figure 3F). Median assignment proportion recorded a median of 59.4% (Figure 3G). The majority of datasets were classified as unstranded (67.3%) with smaller numbers of runs biased towards each strand (Figure 3H). Median proportion of reads mapped with Kallisto was 63.2% (Figure 3I).

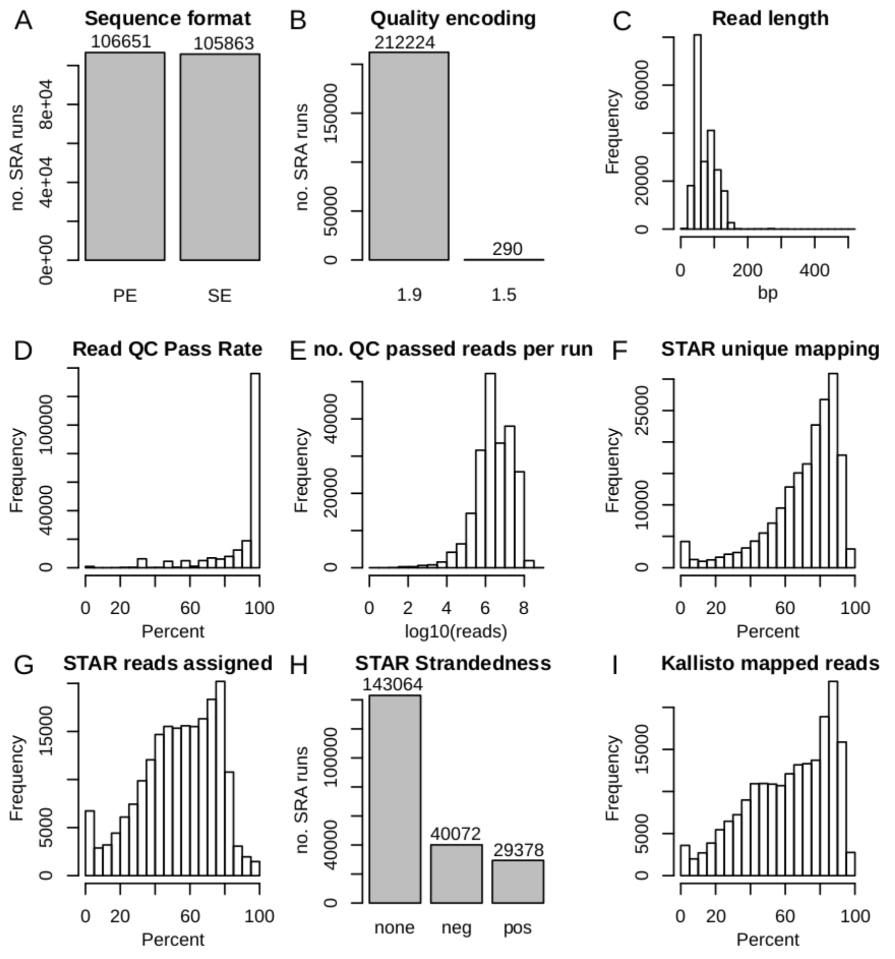

Figure 3. Summary QC metrics for human datasets. (A) sequence format. (B) Base quality encoding, Illumina version 1.9 and 1.5. (C) Read length histogram. (D) Proportion of reads that pass QC filtering. (E) Number of QC passed reads per run. (F) Proportion of STAR uniquely mapped reads. (G) Proportion of reads assigned to genes. (H) Classification of reads by strandedness. (I) Proportion of reads mapped with Kallisto. Data accessed 2018-12-20.

Although there are no definitive thresholds for what constitutes a “valid” RNA-seq dataset, there are two main principles; (i) the digital nature of RNA-seq means that datasets with more reads will provide more accurate quantification, and (ii) datasets with large proportion of reads excluded from downstream analysis will be less representative of the original sample, and suggest issues with sample quality, library preparation or sequencing instrumentation. Using these principles, we have classified the datasets as “pass”, “warn” and “fail” according to heuristics outlined in Table 2. Each rule has a numeric code, and this is provided in the search results. As sequencing depth recommendations are larger for more complex organisms, the metrics describing integer counts are proportional to transcriptome complexity; the number of protein coding genes as defined by Ensembl.

Furthermore, if a dataset profile is substantially different to the bulk of “pass” datasets, this may be useful information for end users. To quantify this, an average gene expression profile (STAR) of “pass” datasets is calculated and each dataset is compared by Pearson correlation (Methods). If the correlation coefficient is less than 0.5 then the dataset is flagged as “warn”. As new datasets are periodically added, these correlation values may vary slightly over time.

Table 2. Criteria for dataset quality classification. *Number of protein coding genes was obtained from Ensembl and used as an estimator of transcriptome complexity.

Metric	Meaning	Fail threshold	Warn threshold	Code
NumReadsQcPass	No. reads passed QC filtering	< 50 reads per gene*	< 500 reads per gene*	1
QcPassRate	Proportion of reads passed QC filtering	< 60%	< 80%	2
STAR_UniqMapRate	Proportion of reads mapped uniquely to the reference genome using STAR	<50%	<70%	3
STAR_AssignRate	Proportion of reads assigned to genes with STAR	<40%	<60%	4
STAR_AssignedReads	No. reads assigned to genes with STAR	< 50 reads per gene*	< 500 reads per gene*	5
Kallisto_MapRate	Proportion of reads assigned to transcripts with Kallisto	<40%	<60%	6
Kallisto_MappedReads	No. reads assigned to transcripts with Kallisto	< 50 reads per gene*	< 500 reads per gene*	7

DatasetCorrel	Pearson correlation coefficient to passed data average	-	< 0.5	8

To understand why some datasets have low correlation to the bulk of “pass” datasets, we undertook an unsupervised clustering analysis of correlation in 5808 *S. cerevisiae* datasets. While most Spearman correlation coefficients were > 0.7, there is a small fraction < 0.5 (Figure 4A). Most datasets (5571) were classified into two large clusters (Figure 4B; blue and light blue). The remaining 236 datasets belonged to several smaller clusters. These smaller clusters mostly contained datasets derived from non-standard RNA-seq library construction protocols such as 3’ end RNA sequencing (ERP004367, SRP048715, SRP048715, SRP021938), Ribo-Seq (SRP075766, SRP082147) and RNA-IP-Seq (SRP032276). One of the smaller clusters contained datasets of cells undergoing sporulation and meiosis (eg: SRP092588,SRP061166, SRP032309). From this analysis, we can conclude that highly correlated datasets are standard RNA-seq/mRNA-seq and datasets with low correlation are mostly due to the use of non-standard library construction protocols, but also some datasets derived from less characterised biological states (eg: meiosis/sporulation in the case of *S. cerevisiae*).

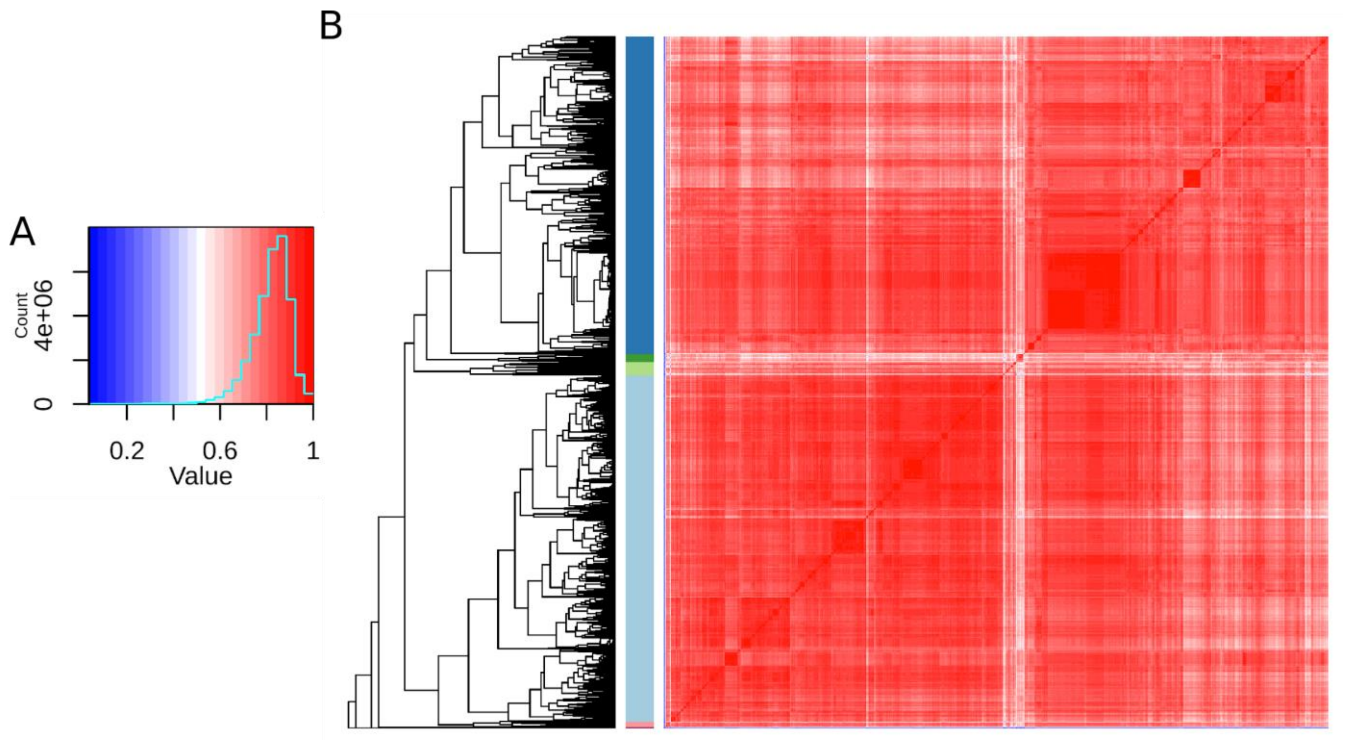

Figure 4. Unsupervised clustering analysis of the correlation of 5807 *S. cerevisiae* datasets.

(A) Colour key and histogram of Spearman correlation coefficients. (B) Heatmap of pairwise correlation values with datasets clustered by similarity. Red indicates high correlation and blue indicates low correlation.

PIPELINE VALIDATION

To demonstrate the accuracy of the pipeline, we performed a simulation study. Synthetic Illumina HiSeq RNA-seq data were generated from Ensembl transcripts and processed with the pipeline (see Methods). The reads per million (RPM) values were compared between the ground truth and DEE2-processed data, and Spearman correlation coefficients (ρ) were calculated (Figure 5; Supplementary Table 2). We observed that analyses of simpler organisms were, in general, more accurate than for more complex transcriptomes of human and mouse. Overall there was only a small improvement in accuracy in paired end over single end reads. Transcript quantification results from Kallisto were less accurate than gene level quantification with STAR. On the other hand, Kallisto transcript counts collapsed into their parent gene were substantially more accurate than STAR gene counts (Figure 5; Supplementary Table 2), consistent with previous a previous report [22].

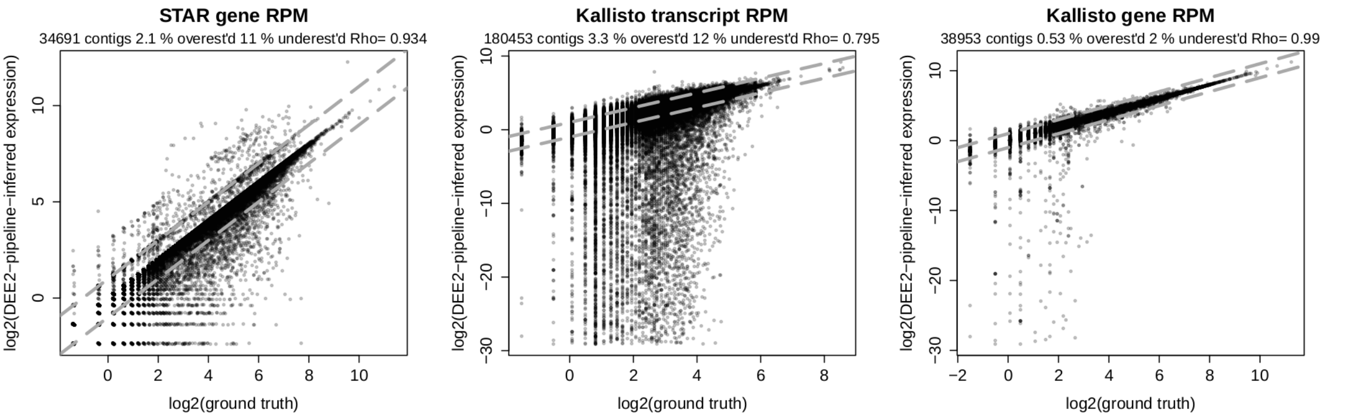

Figure 5. Comparison of ground truth and DEE2 pipeline inferred expression profiles. Human single end 100 bp RNA-seq reads simulated with ART [23] underwent mapping with the DEE2 pipeline, generating gene level and transcript level expression counts. Inferred expression count values were normalized for library size and plotted against the corresponding ground truth values. Dashed lines show the 2 and -2 fold expression differences. STAR gene counts were generated with the "--quantMode GeneCounts" feature. Kallisto estimated counts were used to quantify transcripts. Kallisto gene counts were calculated by aggregating (sum) estimated transcript counts to their parent gene.

In a separate validation exercise, we compared author supplied expression count data present in GEO with corresponding DEE2-STAR counts, and quantified the similarity at the level of individual runs as well as across contrasts (see Methods for details). At the level of individual runs, there was a tight correlation between DEE2-derived and author supplied RPM values, with Spearman coefficients in the range of 0.95-0.99 (Figure 6A). After differential expression (DE) analysis with edgeR [24], genes were ranked by significance. Author derived DE results were then compared to DEE2 derived DE results, enabling us to generate a single Spearman correlation coefficient for each contrast. Using this approach, the correlation in differential expression results between DEE2-STAR and author supplied counts ranged between 0.55 and 0.95 with a median of 0.81 (Figure 6B). Differential expression correlation was higher in comparisons with more replicates (ρ=0.757, n=9, p=0.018). Both exercises support the validity of DEE2 data.

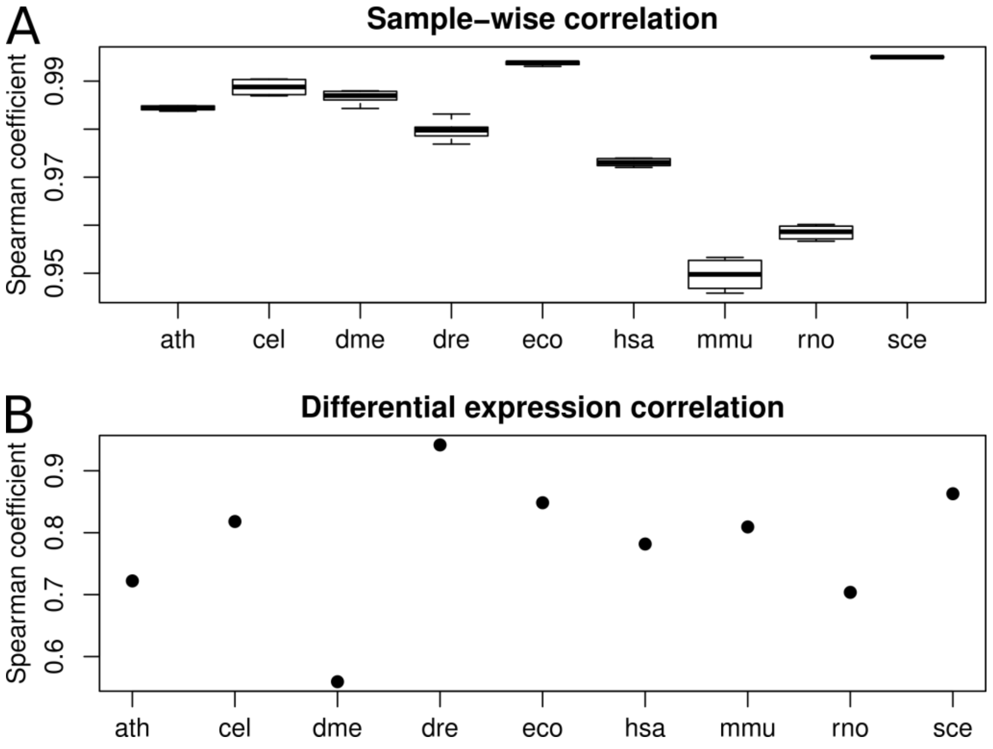

Figure 6. Comparison of DEE2 data with author uploaded gene-level count data. (A) Sample level RPM correlation between author provided and DEE2 processed expression profiles. (B) Correlation of differential expression results. Ath; *A. thaliana*, cel; *C. elegans*, dme; *D. melanogaster*, dre; *D. rerio*, eco; *E. ecoli*, hsa; *H. sapiens*, mmu; *M. musculus*, rno; *R. norvegicus*, sce; *S. cerevisiae*.

A BRIEF META-ANALYSIS OF YEAST GENE EXPRESSION

To demonstrate the utility of DEE2 data we undertook an exploratory analysis of gene expression in *S. cerevisiae*. We correlated the expression of all genes in 5808 datasets in DEE2 and performed unsupervised hierarchical clustering. This resulted 7126 genes being classified into ten clusters (Figure 7A). The largest cluster consisted of 3634 genes (light blue), and the remaining clusters contained between 634 and 175 genes. Gene ontology analysis was performed to detect over-represented biological pathways in each cluster (Figure 7B). Interestingly, each cluster was involved in different biochemical specialisations. For example, the dark green cluster was overrepresented in genes involved in translation, while the nearest neighbour, light purple, was overrepresented in amino acid metabolism. Similarly the light orange cluster was enriched for genes involved in mitochondrial function and the nearest neighbour, pink, was involved in ATP metabolism. These findings illustrate one way in which DEE2 facilitates metaanalysis of gene expression.

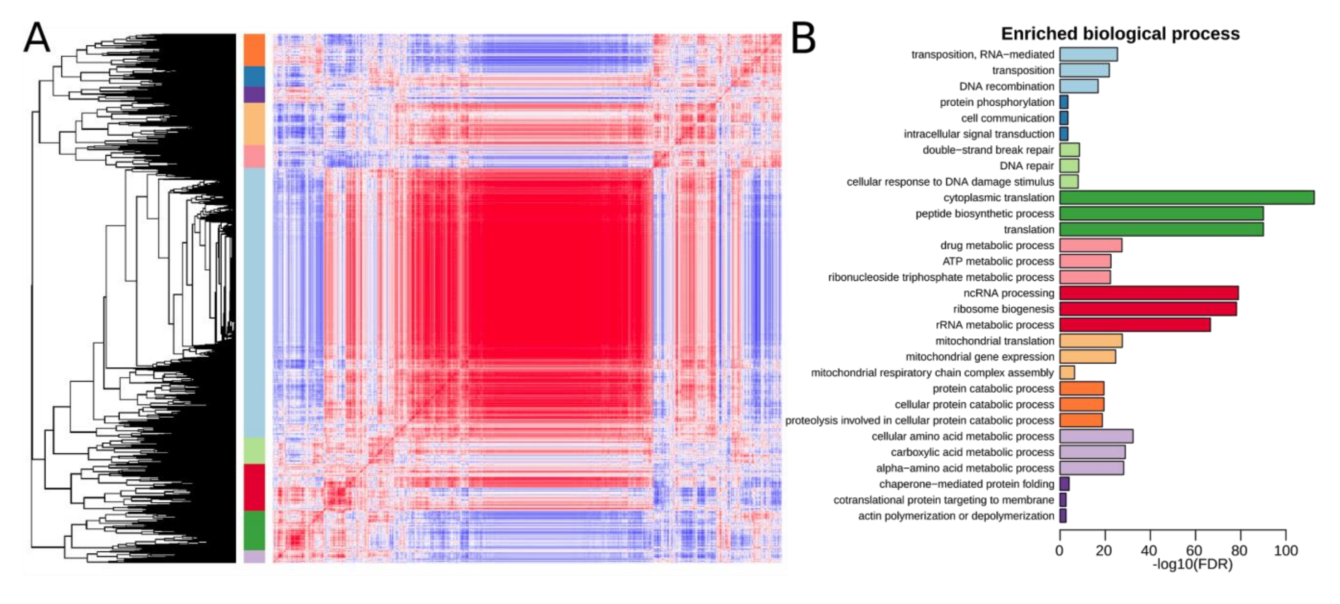

Figure 7. Unsupervised hierarchical clustering of gene expression in *S. cerevisiae*. (A) Clustering and correlation heatmap. Red indicates high correlation and blue indicates low correlation. (B) Biological process gene ontology enrichments of each cluster. Only the top 3 biological processes are shown (as ranked by significance).

RE-USE POTENTIAL

The financial cost of generating these raw datasets is substantial. A rough estimate of the cost to generate raw data included in DEE2 is ~$162 million USD[b]. In contrast, the estimated cost to process these datasets on Amazon EC2 infrastructure is estimated at just $97,000 but could be reduced to about ~$24,000 using off-peak resources[c]. Therefore data aggregation efforts like DEE2 can, with a modest budget, add substantial value to these existing data by enabling straight-forward re-use. Another benefit of aggregation is that genome annotations are updated over time as compared to author-submitted data that remain static.

To enhance the re-use potential, we have designed a simple and easy to use website to access the data. Users select one of the nine species featured and provide either keywords or accession numbers to identify datasets of interest (Figure 8A). The web interface provides datasets in batches of up to 500 runs. When 501 to 5000 matches are obtained, users can download the corresponding metadata and are given options to access expression data (see below). The search results page contains corresponding SRA accession numbers, experiment title and keyword context if a keyword was used (Figure 8B). The results page provides links to QC information so that users can be assured of dataset quality. If ≤500 matches are found, the QC information can be seen simply by hovering the mouse over the QC summary field (Figure 8B). Users then tick the box of every dataset they would like to download and by hitting the “Get Counts” button, the datasets are downloaded. The searching and retrieval steps for the example depicted in Figure 8 took 13 seconds. Figure 8C demonstrates how data is delivered to end users; as a zip archive containing tab-separated expression count, contig information, metadata and quality control information. The webserver is limited to fetching 500 datasets at a time. To enable easy access to large datasets we provide zip “bundles” for each project with ≥200 runs

that have been fully processed by DEE2; there are 425 such bundles as of 11th January 2019 (http://dee2.io/bundles).

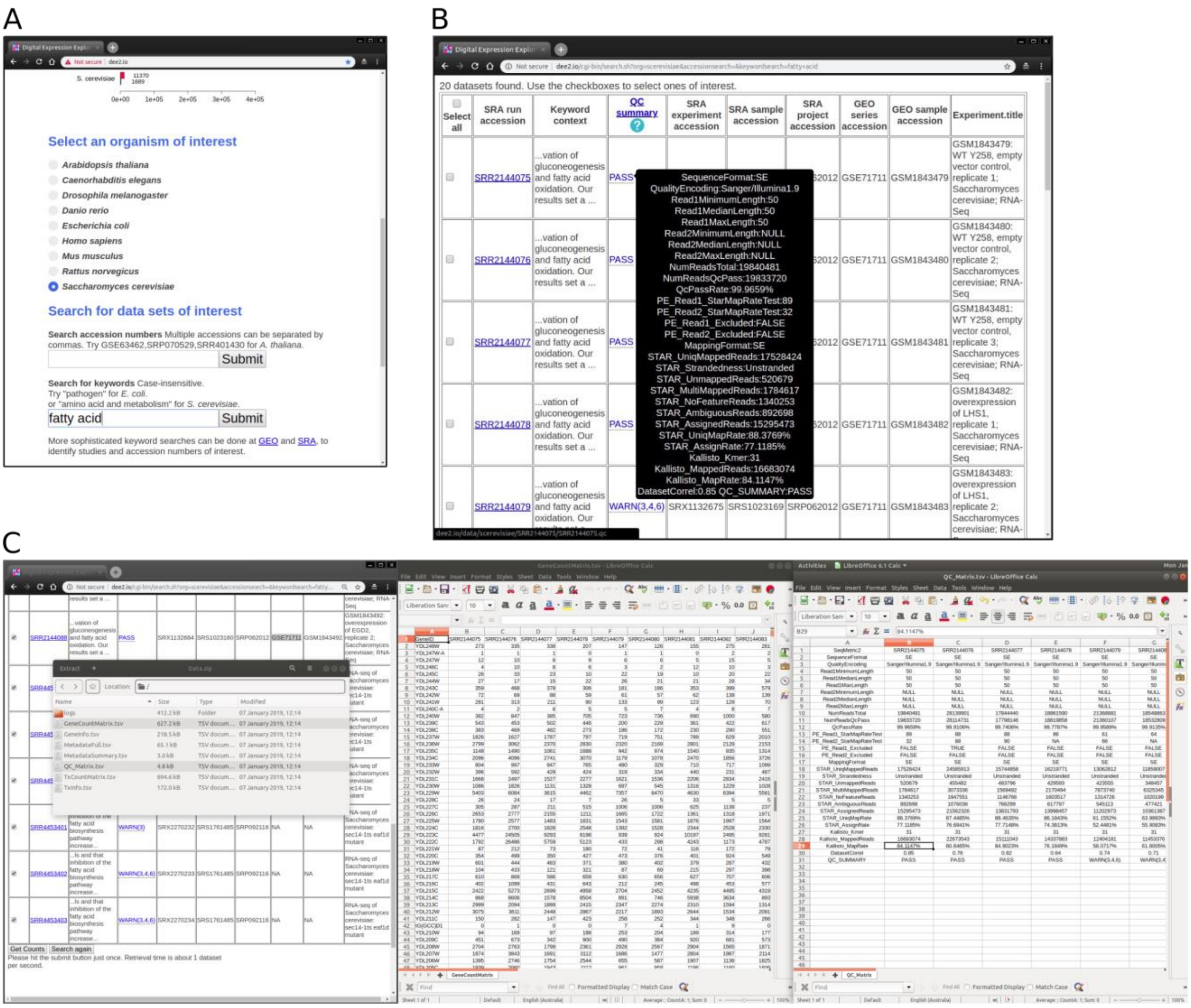

Figure 8. Example of the DEE2 web interface. (A) Users select the species of interest and supply either accession numbers or keywords to search. (B) In this case, a search for “fatty acid” for *S. cerevisiae* yields 20 hits. Mouse pointer hovering over the QC summary field shows the key quality criteria of the processed datasets. (C) Users can select the datasets to download by ticking the boxes and hitting the “Get counts” button at the bottom of the page. The downloaded zip file (C left side) contains detailed processing logs of each run, along with a matrix of gene-wise counts, transcript-wise counts, metadata information, QC information and information on transcripts and genes. The gene-wise count matrix and QC information are shown in C, middle and right side respectively.

As R is the main language for downstream statistical analysis of RNA-seq data, we also provide an R package “getDEE2” to obtain DEE2 data. In the example shown in Box 1, datasets belonging to an experiment with GEO series GSE33569 are obtained. Transcript wise counts can be aggregated to gene-level counts with a single command (Tx2Gene).

```
#obtain the source code
> library("devtools")
> devtools::install_github("markziemann/dee2/getDEE2")
> library("getDEE2")
#obtain DEE2 metadata
> mdat<-getDee2Metadata("celegans")
trying URL 'http://dee2.io/metadata/celegans_metadata.tsv.cut'
Content type 'text/tab-separated-values' length 1015195 bytes (991 KB)
==================================================
downloaded 991 KB

#Browse metadata for GEO series GSE33569
> mdat[which(mdat$GSE_accession %in% "GSE33569"),]
     SRR_accession      QC_summary SRX_accession SRS_accession SRP_accession
2444     SRR363796 FAIL(2,3,4,6,7)     SRX105188     SRS270025     SRP009256
3650     SRR363798 FAIL(2,3,4,6,7)     SRX105190     SRS270027     SRP009256
5775     SRR363797   FAIL(3,4,6,7)     SRX105189     SRS270026     SRP009256
6797     SRR363799   FAIL(3,4,6,7)     SRX105191     SRS270028     SRP009256
     GSE_accession GSM_accession              experiment_title
2444      GSE33569     GSM829554 GSM829554: 4SU_GLD1_PARCLIP_1
3650      GSE33569     GSM829556 GSM829556: 4SU_GLD1_PARCLIP_3
5775      GSE33569     GSM829555 GSM829555: 4SU_GLD1_PARCLIP_2
6797      GSE33569     GSM829557 GSM829557: 6SG_GLD1_PARCLIP_1

> mdat1<-mdat[which(mdat$GSE_accession %in% "GSE33569"),]
> SRRlist<-as.vector(mdat1$SRR_accession)
> SRRlist
[1] "SRR363796" "SRR363797" "SRR363798" "SRR363799"

> x<-getDEE2("celegans",SRRlist)
trying URL 'http://dee2.io/cgi-
bin/request.sh?org=celegans&x=SRR363796&x=SRR363798&x=SRR363797&x=SRR363799'
downloaded 1.2 MB

For more information about DEE2 QC metrics, visit
https://github.com/markziemann/dee2/blob/master/qc/qc_metrics.md

> names(x)
[1] "GeneCounts"      "TxCounts"        "GeneInfo"        "TxInfo"
[5] "QcMx"            "MetadataSummary" "MetadataFull"    "absent"
> head(x$GeneCounts)
               SRR363796 SRR363797 SRR363798 SRR363799
WBGene00197333         0         0         0         0
WBGene00198386         0         0         0         0
WBGene00015153         4        16         6         4
WBGene00002061        44       100       217        77
> head(x$TxCounts)
           SRR363796 SRR363797 SRR363798 SRR363799
Y110A7A.10        11        23        48        45
F27C8.1            0         0         0         0
F07C3.7            0         2         0        21
F52H2.2a           0         0         7         0
> head(x$QcMx)
                           SRR363796           SRR363798           SRR363797
SequenceFormat                    SE                  SE                  SE
QualityEncoding    Sanger/Illumina1.9 Sanger/Illumina1.9 Sanger/Illumina1.9
Read1MinimumLength                36                  36                  36
Read1MedianLength                 36                  36                  36
                            SRR363799
SequenceFormat                    SE
QualityEncoding    Sanger/Illumina1.9
Read1MinimumLength                36
Read1MedianLength                 36

> head(x$GeneInfo)
               GeneSymbol mean median longest_isoform merged
```

```
WBGene00197333   cTel3X.2  150    150             150    150
WBGene00198386   cTel3X.3  150    150             150    150
WBGene00015153    B0348.5 1051   1178            1178   1178
WBGene00002061      ife-3 1015    949            1107   1107
> head(x$TxInfo)
                  GeneID GeneSymbol TxLength
Y110A7A.10 WBGene00000001      aap-1     1787
F27C8.1    WBGene00000002      aat-1     1940
F07C3.7    WBGene00000003      aat-2     1728
F52H2.2a   WBGene00000004      aat-3     1739
> x<-Tx2Gene(x)
> names(x)
[1] "Tx2Gene"         "GeneCounts"      "TxCounts"        "GeneInfo"
[5] "TxInfo"          "QcMx"            "MetadataSummary" "MetadataFull"
[9] "absent"
> head(x$Tx2Gene)
               SRR363796 SRR363797 SRR363798 SRR363799
WBGene00000001        11        23        48        45
WBGene00000002         0         0         0         0
WBGene00000003         0         2         0        21
WBGene00000004         0         4         7         5
```

Box 1. An example of obtaining gene and transcript expression datasets using the R functions (GEO series: GSE33569). The Tx2Gene function is used to aggregate (sum) transcript counts to gene-level counts.

For power users, bulk data dumps are available and will be of use to researchers wishing to do wholesale meta-analyses, as three studies already have [25-27]. Irrespective of the method of acquisition, DEE2 data are compatible with many different downstream applications including R/Bioconductor [28,29], Degust [30] and Galaxy [31].

CONCLUSION

DEE2 provides a unique framework and user-friendly resource of processed RNA-seq data that alleviates many of the bottlenecks researchers currently face with analysis of public RNA-seq data. Our testing shows DEE2 and Degust enable analysis of public RNA-seq data on mobile devices such as smartphones. Bulk data provided by DEE2 is a useful starting-point for researchers performing meta-analyses of RNA-seq data.

METHODS

Analysis of correlation

Correlation of a dataset to the bulk of other high quality datasets could be a useful metric to filter by when performing meta-analyses. To make this a tractable calculation, first an mean

gene expression profile is generated using STAR gene expression data from up to 10,000 randomly selected datasets that pass all other QC checks. Next, each dataset is compared to the average pass profile and a Pearson correlation coefficient is calculated. As new datasets will be added to DEE2 regularly, the average pass profile will vary slightly over time and as such, the correlation coefficient is calculated to two significant figures only.

In the analysis of dataset correlation (Figure 4), the Spearman correlation of all 5808 *S. cerevisiae* datasets classified as “pass” and “warn” was calculated in a pairwise fashion (data as at 3rd Jan 2019). Clustering was performed using the “hclust” function in R with the complete linkage method, followed by tree cutting at 0.375 of the maximum branch length. The heatmap.2 package was used to generate the heatmap. In the analysis of gene expression correlation (Figure 7A), the *S. cerevisiae* data were transposed prior to correlation analysis, then tree cutting was performed at 0.17 of the maximum branch length. The clusters obtained were subjected to gene ontology analysis at the level of biological pathways using the “enrichGO” tool in the clusterProfiler package version 3.8.1 (clusterProfiler, RRID:SCR_016884)[32]. Only the top three pathways for each cluster were plotted.

Pipeline validation using simulated data

To validate the accuracy of the DEE2 pipeline, we generated Illumina HiSeq2500-like sequence reads from Ensembl cDNA sequences using ART (v2016-06-05)[23] with a defined seed (1540165885) and a uniform fold coverage of 2. Read lengths were 50 and 100 bp in single and paired end format. The read sets were processed with the DEE2 pipeline and the observed expression data were compared to the ground truth, using Spearman correlation of library size normalised profiles in reads per million (RPM) as an indicator of accuracy. For Kallisto based analysis, estimated transcript counts “est_counts” were used. Transcript estimated counts were totalled for each parent gene to to generate gene-wise expression counts. These analyses were performed for all nine organisms currently included in DEE2.

Pipeline validation using public data

Another way to validate the accuracy of DEE2 data is to compare it to author submitted results available on GEO. We searched for studies that reported expression data as raw counts with official gene names or Ensembl accession numbers, two or more replicates and acceptable read depth and genome mapping rate. Author supplied counts were obtained from GEO for the datasets listed in Table 3 [33-41]. Spearman correlation of RPM values was used to quantify the similarity of DEE2 and author-supplied data at the level of individual runs. To determine the similarity in differential expression results, the same edgeR v3.22.3 [24] analysis was performed in parallel on author supplied counts and DEE2 counts. The runs defined as control and case for each experiment are listed in Table 3. To rank genes by significance in differential expression, the sign of the fold change was multiplied by the negative log2 p value. Spearman correlation analysis was used to quantify the similarity in differential expression results using these two data sources.

Table 3. Details of author-supplied processed data used to compare to DEE2 gene expression counts.

Species and GEO series	Contrast (control / case)	Spots	Author pipeline
A. thaliana GSE53078 [33]	GSM1281703<br>GSM1281704	15,143,653<br>12,498,123	Genome: TAIR10<br>Annotation version: Unknown<br>Mapper: TopHat<br>Counter: HTSeq
	GSM1281705<br>GSM1281706	22,721,359<br>17,255,612	
C. elegans GSE46344 [34]	GSM1128862<br>GSM1128863<br>GSM1128864	30,650,959<br>47,245,721<br>54,573,311	Genome: WS220/ce10<br>Annotation: Ensembl v66<br>Mapper: TopHat<br>Counter: HTSeq
	GSM1128868<br>GSM1128869<br>GSM1128870	49,315,179<br>56,295,663<br>68,641,842	
D. melanogaster GSE43180 [35]	GSM1057982<br>GSM1057983	24,902,977<br>36,434,276	Genome: dm3<br>Annotation: Ensembl v64<br>Mapper: Tophat<br>Counter: HTSeq
	GSM1057984<br>GSM1057985	32,591,508<br>35,375,654	
D. rerio GSE80768 [36]	GSM2136810<br>GSM2136811<br>GSM2136812<br>GSM2136813<br>GSM2136814<br>GSM2136815<br>GSM2136816<br>GSM2136817	19,404,674<br>22,820,115<br>25,181,184<br>21,487,514<br>24,831,643<br>22,664,352<br>22,629,782<br>21,842,104	Genome:Zv10<br>Annotation: Ensembl (version unknown)<br>Mapper: USeq and Novoalign<br>Counter: USeq
	GSM2136818<br>GSM2136819<br>GSM2136820<br>GSM2136821<br>GSM2136822<br>GSM2136823<br>GSM2136824	20,601,291<br>18,183,746<br>21,007,467<br>20,992,396<br>24,708,106<br>21,105,462<br>28,069,482	
E. coli GSE80251 [37]	GSM2122743<br>GSM2122744<br>GSM2122745	5,221,858<br>6,503,454<br>6,209,263	Genome: E. coli K12 MG1655<br>Annotation: GenBank NC_000913.3<br>Mapper: TMAP (map4)<br>Counter: Bedtools
	GSM2122746<br>GSM2122747<br>GSM2122748	6,391,549<br>6,197,872<br>5,090,669	
H. sapiens GSE63776 [38]	GSM1556982<br>GSM1556983<br>GSM1556984	30,007,994<br>27,252,897<br>42,212,497	Genome: hg19<br>Annotation: UCSC (version unknown)<br>Mapper: Bowtie2 (after adapter clipping)<br>Counter: HTSeq
	GSM1556985<br>GSM1556986<br>GSM1556987	31,456,271<br>31,569,339<br>37,477,777	
M. musculus GSE59970 [39]	GSM1462883<br>GSM1462884<br>GSM1462885	32,015,112<br>30,997,187<br>32,612,584	Genome: GRCm38.70/mm10<br>Annotation: Ensembl v70<br>Mapper: Olego<br>Counter: BedTools
	GSM1462886<br>GSM1462887<br>GSM1462888	31,485,760<br>30,207,461<br>31,028,501	
R. norvegicus GSE65715 [40]	GSM1604049<br>GSM1604050<br>GSM1604051	42,296,446<br>34,887,323<br>42,725,865	Genome: rn4<br>Annotation:Ensembl (version unknown)<br>Mapper: Tophat2<br>Counter: HTSeq
	GSM1604052<br>GSM1604053<br>GSM1604054	28,210,194<br>30,748,641<br>28,450,626	
S. cerevisiae GSE76444 [41]	GSM2809655<br>GSM2809656<br>GSM2809657	35,869,614<br>37,425,737<br>39,227,797	Genome: EF 4<br>Annotation: Ensembl v72<br>Mapper: Bowtie

	GSM2809658<br>GSM2809659<br>GSM2809660	33,974,055<br>33,339,067<br>37,546,069	Counter: HTSeq

AVAILABILITY OF SOURCE CODE AND REQUIREMENTS

- *Project name: Digital Expression Explorer 2*
- *Project home page: http://dee2.io*
- *Operating systems (dataset): Platform independent*
- *Operating systems (pipeline): Unix and MacOS*
- *License: GNU GPL v3*
- *Any restrictions to use by non-academics: none*

AVAILABILITY OF SUPPORTING DATA

- *Dataset access: http://dee2.io (RRID:SCR_016929)*
- *Bulk data access: https://datbase.org/dee2/bulk*
- *Source code: https://github.com/markziemann/dee2 (RRID:SCR_016930)*
- *Pipeline Docker image: https://hub.docker.com/r/mziemann/tallyup/ (RRID:SCR_016931)*

A snapshot of the latest update of the bulk data presented in this manuscript is available in the *GigaScience* GigaDB repository[42].

DECLARATIONS

Abbreviations

DEE2; Digital Expression Explorer 2, GEO; Gene Expression Omnibus, SRA; Sequence Read Archive, SRAdbV2; An R Package to Query the Sequence Read Archive, QC; quality control, SE; single end, PE; paired end.

Competing interests

The authors declare that they have no competing interests.

Funding

AE-O is a Senior Research Fellow supported by NHMRC. AE-O receives funding from the National Health and Medical Research Council – Natural Science Foundation of China (NHMRC-NSFC International Joint Call APP1113188).

Authors' contributions

MZ and AE-O conceived and designed the study. MZ and AK wrote the computer code. MZ coordinated data processing and drafted the manuscript. All authors read, revised and approved the final manuscript.

Acknowledgments

This research was made possible by use of the Multi-modal Australian ScienceS Imaging and Visualisation Environment (MASSIVE) and Nectar Research Cloud, both supported by the Australian National Collaborative Research Infrastructure Strategy (NCRIS). This work was supported by Deakin eResearch and Monash eResearch Centres. We thank Dr Ross Lazarus and Dr Haloom Rafehi for bioinformatics expertise, advice and helpful discussions. We thank Julian Vreugdenburg for technical support. We thank the many users that have provided feedback on earlier versions of DEE2.

ENDNOTES

[a] These 9 species represent 822,819 of the 1,119,784 RNA experiments present in SRA as at 29th Oct 2018.

[b] Estimated cost (in USD) of generating 5.3 trillion 100 bp SE reads from 399,377 experiments. Cornell University Institute of Biotechnology advertises HiSeq2500 at $18,407 for 8 lanes 100bp

SE [43]. Illumina HiSeq2500 v4 spec sheet estimates 1.5 billion reads per 8 lane flow cell [44]. That gives $12.27 per million reads. The cost of sequencing is $65.0 million. Library prep costs approximately $200 per sample which is in the range advertised at Cornell multiplied by 482,879 experiments equates to $96.6 million for library construction. Grand total of $162 million.

[c] Estimated cost of Amazon EC2 pricing is based on the mean elapsed time for processing a dataset being 894 s on a 16 thread Intel Xeon E3-12xx v2 (Ivy Bridge, IBRS) 2.6 GHz, 64 GB RAM with ~400 MB/s download speed from NCBI. The equivalent Amazon instance is r5d.2xlarge and would require a 500 GB volume. The elapsed time is based on observations from a set of 705 human datasets. The breakdown for the total on demand cost is $83,697 for compute, $131 for data transfer and $12,750 for persistent data volume. The off-peak cost breakdown is $11,544 for compute, $131 for data transfer and $12,750 for persistent data volume. The persistent data volume cost is valid if using 17 instances continuously for a full year. Compute price is based on US East location (Ohio) and is more expensive at other locations.

REFERENCES

1. Nagalakshmi U, Wang Z, Waern K, Shou C, Raha D, Gerstein M, Snyder M. The transcriptional landscape of the yeast genome defined by RNA sequencing. Science. 2008;320:1344-9.

2. Barrett T, Wilhite SE, Ledoux P, Evangelista C, Kim IF, Tomashevsky M, Marshall KA, Phillippy KH, Sherman PM, Holko M, Yefanov A, Lee H, Zhang N, Robertson CL, Serova N, Davis S, Soboleva A. NCBI GEO: archive for functional genomics data sets--update. Nucleic Acids Res. 2013;41:D991-5.

3. Kodama Y, Shumway M, Leinonen R; International Nucleotide Sequence Database Collaboration. The Sequence Read Archive: explosive growth of sequencing data. Nucleic Acids Res. 2012;40:D54-6.

4. Collado-Torres L, Nellore A, Kammers K, Ellis SE, Taub MA, Hansen KD, Jaffe AE, Langmead B, Leek JT. Reproducible RNA-seq analysis using recount2. Nat Biotechnol. 2017;35:319-21.

5. Lachmann A, Torre D, Keenan AB, Jagodnik KM, Lee HJ, Wang L, Silverstein MC, Ma'ayan A. Massive mining of publicly available RNA-seq data from human and mouse. Nat Commun. 2018;9:1366.

6. Hruz T, Laule O, Szabo G, Wessendorp F, Bleuler S, Oertle L, Widmayer P, Gruissem W, Zimmermann P. Genevestigator v3: a reference expression database for the meta-analysis of transcriptomes. Adv Bioinformatics. 2008;2008:420747.

7. Bastian F., Parmentier G., Roux J., Moretti S., Laudet V., Robinson-Rechavi M. Bgee: Integrating and Comparing Heterogeneous Transcriptome Data Among Species. In: Bairoch A., Cohen-Boulakia S., Froidevaux C. (eds) Data Integration in the Life Sciences. DILS 2008. Lecture Notes in Computer Science, vol 5109. Springer, Berlin, Heidelberg. DOI https://doi.org/10.1007/978-3-540-69828-9_12

8. Papatheodorou I, Fonseca NA, Keays M, Tang YA, Barrera E, Bazant W, Burke M, Füllgrabe A, Fuentes AM, George N, Huerta L, Koskinen S, Mohammed S, Geniza M, Preece J, Jaiswal P, Jarnuczak AF, Huber W, Stegle O, Vizcaino JA, Brazma A, Petryszak R. Expression Atlas: gene and protein expression across multiple studies and organisms. Nucleic Acids Res. 2018;46:D246-D251.

9. Davis S. The SRAdbV2 Package. 2018. https://github.com/seandavi/SRAdbV2. Accessed 16 Oct 2018

10. Ziemann M. Tally-up: Bulk reprocessing of RNA-seq data. 2018. https://hub.docker.com/r/mziemann/tallyup. Accessed 16 Oct 2018.

11. Kurtzer GM, Sochat V, Bauer MW. Singularity: Scientific containers for mobility of compute. PLoS One. 2017;12:e0177459.

12. Gomes J, Bagnaschi E, Campos I, David M, Alves L, Martins J, Pina J, López-García A, Orviz P. Enabling rootless Linux Containers in multi-user environments: The udocker tool. Comput Phys Commun. 2018;232:84-97.

13. Zerbino DR, Achuthan P, Akanni W, Amode MR, Barrell D, Bhai J, Billis K, Cummins C, Gall A, Girón CG, Gil L, Gordon L, Haggerty L, Haskell E, Hourlier T, Izuogu OG, Janacek SH, Juettemann T, To JK, Laird MR, Lavidas I, Liu Z, Loveland JE, Maurel T,

McLaren W, Moore B, Mudge J, Murphy DN, Newman V, Nuhn M, Ogeh D, Ong CK, Parker A, Patricio M, Riat HS, Schuilenburg H, Sheppard D, Sparrow H, Taylor K, Thormann A, Vullo A, Walts B, Zadissa A, Frankish A, Hunt SE, Kostadima M, Langridge N, Martin FJ, Muffato M, Perry E, Ruffier M, Staines DM, Trevanion SJ, Aken BL, Cunningham F, Yates A, Flicek P. Ensembl 2018. Nucleic Acids Res. 2018;46:D754-D761.

14. Andrews, S. FastQC: a quality control tool for high throughput sequence data. 2010. http://www.bioinformatics.babraham.ac.uk/projects/fastqc. Accessed 5th March 2018.

15. Valeris, R. Parallel-fastq-dump. 2016. https://github.com/rvalieris/parallel-fastq-dump. Accessed 5th March 2018.

16. Jiang H, Lei R, Ding SW, Zhu S. Skewer: a fast and accurate adapter trimmer for next-generation sequencing paired-end reads. BMC Bioinformatics. 2014;15:182.

17. Davis MP, van Dongen S, Abreu-Goodger C, Bartonicek N, Enright AJ. Kraken: a set of tools for quality control and analysis of high-throughput sequence data. Methods. 2013;63:41-9.

18. Dobin A, Davis CA, Schlesinger F, Drenkow J, Zaleski C, Jha S, Batut P, Chaisson M, Gingeras TR. STAR: ultrafast universal RNA-seq aligner. Bioinformatics. 2013;29:15-21.

19. Bray NL, Pimentel H, Melsted P, Pachter L. Near-optimal probabilistic RNA-seq quantification. Nat Biotechnol. 2016;34:525-7.

20. Ziemann, M. Digital Expression Explorer 2 (DEE2): a repository of uniformly processed RNA-seq data. 2018. https://github.com/markziemann/dee2. Accessed 11 Jan 2019.

21. Li HD, GTFtools: a Python package for analyzing various modes of gene models. bioRxiv. 2018;263517.

22. Soneson C, Love MI, Robinson MD. Differential analyses for RNA-seq: transcript-level estimates improve gene-level inferences. Version 2. F1000Res. 2015;4:1521.

23. Huang W, Li L, Myers JR, Marth GT. ART: a next-generation sequencing read simulator. Bioinformatics. 2012;28:593-4.

24. Robinson MD, McCarthy DJ, Smyth GK. edgeR: a Bioconductor package for differential expression analysis of digital gene expression data. Bioinformatics. 2010;26:139-40.

25. Rau A, Maugis-Rabusseau C. Transformation and model choice for RNA-seq co-expression analysis. Brief Bioinform. 2017;pii:bbw128.

26. Espinar L, Schikora Tamarit MÀ, Domingo J, Carey LB. Promoter architecture determines cotranslational regulation of mRNA. Genome Res. 2018;28:509-18.

27. Godichon-Baggioni A, Maugis-Rabusseau C, Rau A. Clustering transformed compositional data using K-means, with applications in gene expression and bicycle sharing system data. J Appl Stat. 2018;3:1-9.

28. Ihaka R, Gentleman R. R: A Language for Data Analysis and Graphics. J Comput Graph Stat. 1996;3:299-314.

29. Huber W, Carey VJ, Gentleman R, Anders S, Carlson M, Carvalho BS, Bravo HC, Davis S, Gatto L, Girke T, Gottardo R, Hahne F, Hansen KD, Irizarry RA, Lawrence M, Love MI, MacDonald J, Obenchain V, Oleś AK, Pagès H, Reyes A, Shannon P, Smyth GK, Tenenbaum D, Waldron L, Morgan M. Orchestrating high-throughput genomic analysis with Bioconductor. Nat Methods. 2015;12:115-21.

30. Powell D. Degust: RNA-seq exploration, analysis and visualisation. 2013. http://degust.erc.monash.edu. Accessed 6 Apr 2018.

31. Afgan E, Baker D, van den Beek M, Blankenberg D, Bouvier D, Čech M, Chilton J, Clements D, Coraor N, Eberhard C, Grüning B, Guerler A, Hillman-Jackson J, Von Kuster G, Rasche E, Soranzo N, Turaga N, Taylor J, Nekrutenko A, Goecks J. The Galaxy platform for accessible, reproducible and collaborative biomedical analyses: 2016 update. Nucleic Acids Res. 2016;44:W3-W10.

32. Yu G, Wang LG, Han Y, He QY. clusterProfiler: an R package for comparing biological themes among gene clusters. OMICS. 2012;16:284-7.

33. Fan M, Bai MY, Kim JG, Wang T, Oh E, Chen L, Park CH, Son SH, Kim SK, Mudgett MB, Wang ZY. The bHLH transcription factor HBI1 mediates the trade-off between growth and pathogen-associated molecular pattern-triggered immunity in Arabidopsis. Plant Cell. 2014;26:828-41.

34. Priebe S, Menzel U, Zarse K, Groth M, Platzer M, Ristow M, Guthke R. Extension of life span by impaired glucose metabolism in Caenorhabditis elegans is accompanied by structural rearrangements of the transcriptomic network. PLoS One. 2013;8:e77776.

35. Kockmann T, Gerstung M, Schlumpf T, Xhinzhou Z, Hess D, Beerenwinkel N, Beisel C, Paro R. The BET protein FSH functionally interacts with ASH1 to orchestrate global gene activity in Drosophila. Genome Biol. 2013;14:R18.

36. Modzelewska K, Boer EF, Mosbruger TL, Picard D, Anderson D, Miles RR, Kroll M, Oslund W, Pysher TJ, Schiffman JD, Jensen R, Jette CA, Huang A, Stewart RA. MEK Inhibitors Reverse Growth of Embryonal Brain Tumors Derived from Oligoneural Precursor Cells. Cell Rep. 2016;17:1255-1264.

37. Dzyubak E, Yap MN. The Expression of Antibiotic Resistance Methyltransferase Correlates with mRNA Stability Independently of Ribosome Stalling. Antimicrob Agents Chemother. 2016;60:7178-7188.

38. Gaddis M, Gerrard D, Frietze S, Farnham PJ. Altering cancer transcriptomes using epigenomic inhibitors. Epigenetics Chromatin. 2015;8:9.

39. Sim CB, Ziemann M, Kaspi A, Harikrishnan KN, Ooi J, Khurana I, Chang L, Hudson JE, El-Osta A, Porrello ER. Dynamic changes in the cardiac methylome during postnatal development. FASEB J. 2015;29:1329-43.

40. Behmoaras J, Diaz AG, Venda L, Ko JH, Srivastava P, Montoya A, Faull P, Webster Z, Moyon B, Pusey CD, Abraham DJ, Petretto E, Cook TH, Aitman TJ. Macrophage epoxygenase determines a profibrotic transcriptome signature. J Immunol. 2015 May 15;194(10):4705-4716.

41. Wang C, Schmich F, Srivatsa S, Weidner J, Beerenwinkel N, Spang A. Context-dependent deposition and regulation of mRNAs in P-bodies. Elife. 2018 Jan 3;7. pii: e29815.

42. Ziemann M; Kaspi A; El-Osta A (2019): Supporting data for "Digital Expression Explorer 2: a repository of uniformly processed RNA sequencing data" GigaScience Database. http://dx.doi.org/10.5524/100569

43. Cornell University Institute of Biotechnology Illumina Sequencing Price List. http://www.biotech.cornell.edu/brc/genomics/services/price-list Accessed 24th Oct 2018.

44. Illumina Inc. System Specification Sheet for the HiSeq 2500 System. 2015. https://www.illumina.com/documents/products/datasheets/datasheet_hiseq2500.pdf Accessed 24th Oct 2018.

Supplementary Table 1. Software versions and parameters used in the pipeline.

Software, version	Purpose	Parameter: Single end	Parameter: Paired end
Aspera client, v3.5.4	Rapid download of sequence data	ascp -l 500m -O 33001 -T -i $ID $URL .	
SRA toolkit, v2.8.2	Validate downloaded SRA files	vdb-validate $SRA	
	diagnose single or paired end	fastq-dump -X 4000 --split-files $SRA	
	dump fastq	(see parallel-fastq-dump below)	
FastQC, v0.11.5	Diagnose basespace / colorspace, quality encoding, read length from 4000 reads	fastqc $FQ1	fastqc $FQ2
parallel-fastq-dump, 0.6.3	Rapid decompression of sequence data from .sra files	parallel-fastq-dump --threads $THREADS --outdir . --split-files --defline-qual + -s ${SRR}.sra	
Skewer, v0.2.2	3' quality trimming	skewer -l 18 -q 10 -k inf -t $THREADS -o $SRR $FQ1	skewer -l 18 -q 10 -k inf -t $THREADS -o $SRR $FQ1 $FQ2
	Adapter clipping	skewer -l 18 -t $THREADS -x $ADAPTER -o $SRR $FQ1	skewer -l 18 -t $THREADS -x $ADAPTER1 -y $ADAPTER2 -o $SRR $FQ1 $FQ2
	5' trimming	skewer -m ap --cut $CLIP_NUM,$CLIP_NUM -l 18 -k inf -t $THREADS $FQ1	skewer -m ap --cut $R1_CLIP_NUM,$R2_CLIP_NUM -l 18 -k inf -t $THREADS $FQ1 $FQ2
Minion, v13-100	3' adapter detection	minion search-adapter -i $FQ1	minion search-adapter -i $FQ2
Bowtie2, v2.3.2	Adapter contamination detection	bowtie2 -f -x $BT2_REF -S /dev/stdout $ADAPTER	
FASTX-Toolkit, v0.0.14	Progressive 5' trimming	fastx_trimmer -f {5,9,13,21} -m 18 -Q 33 -i $FQ1	fastx_trimmer -f {5,9,13,21} -m 18 -Q 33 -i $FQ2
STAR v020201	Gene-level mapping, Diagnose strandedness	STAR --runThreadN $THREADS --quantMode GeneCounts --genomeLoad LoadAndKeep \ --outSAMtype None --genomeDir $STAR_DIR --readFilesIn=$FQ1	STAR --runThreadN $THREADS --quantMode GeneCounts --genomeLoad LoadAndKeep \ --outSAMtype None --genomeDir $STAR_DIR --readFilesIn=$FQ1 $FQ2
Kallisto, v0.43.1	Transcript-level mapping	kallisto quant $KALLISTO_STRAND_PARAMETER \ --single -l 100 -s 20 -t $THREADS -o . \ -i $KAL_REF $FQ1	kallisto quant $KALLISTO_STRAND_PARAMETER -t $THREADS -o . -i $KAL_REF $FQ1 $FQ2

Supplementary Table 2. Spearman correlation coefficients (ρ) between ground truth and DEE2 processed expression profiles (RPM) from simulated data.

Species	Seq format	STAR (gene)	Kallisto (transcript)	Kallisto (gene)
A. thaliana	50 bp SE	0.958	0.788	0.998
	100 bp SE	0.957	0.744	0.998
	50 bp PE	0.950	0.722	0.999
	100 bp PE	0.948	0.697	0.997
C. elegans	50 bp SE	0.939	0.805	0.988
	100 bp SE	0.946	0.769	0.984
	50 bp PE	0.939	0.755	0.985
	100 bp PE	0.940	0.699	0.981
D. melanogaster	50 bp SE	0.913	0.836	0.997
	100 bp SE	0.912	0.795	0.997
	50 bp PE	0.905	0.757	0.998
	100 bp PE	0.905	0.731	0.997
D. rerio	50 bp SE	0.924	0.926	0.997
	100 bp SE	0.947	0.907	0.997
	50 bp PE	0.939	0.911	0.998
	100 bp PE	0.953	0.887	0.997
E. coli	50 bp SE	0.980	0.996	0.996
	100 bp SE	0.981	0.999	0.999
	50 bp PE	0.980	0.999	0.999
	100 bp PE	0.982	0.999	0.999
H. sapiens	50 bp SE	0.926	0.822	0.988
	100 bp SE	0.934	0.795	0.990
	50 bp PE	0.922	0.782	0.991
	100 bp PE	0.926	0.767	0.989
M. musculus	50 bp SE	0.924	0.897	0.991
	100 bp SE	0.937	0.868	0.991
	50 bp PE	0.928	0.867	0.993
	100 bp PE	0.936	0.839	0.992
R. norvegicus	50 bp SE	0.882	0.957	0.992
	100 bp SE	0.895	0.946	0.992
	50 bp PE	0.890	0.944	0.993
	100 bp PE	0.897	0.925	0.990
S. cerevisiae	50 bp SE	0.992	0.980	0.980
	100 bp SE	0.929	0.981	0.981
	50 bp PE	0.923	0.986	0.986
	100 bp PE	0.927	0.980	0.980

Figure 1

Click here to access/download;Figure;Figure1.pdf

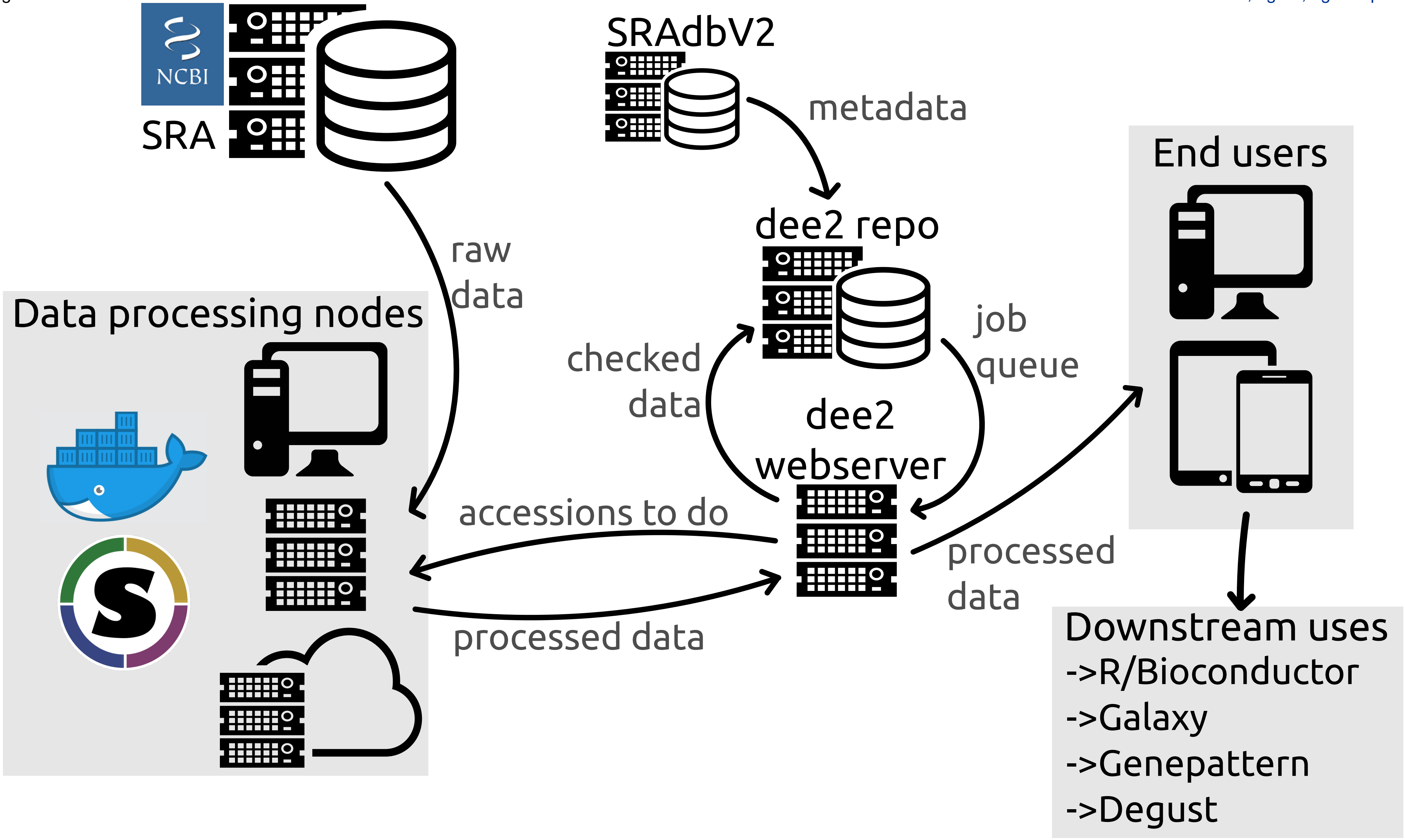

Figure 2

Click here to access/download;Figure;Figure2.pdf

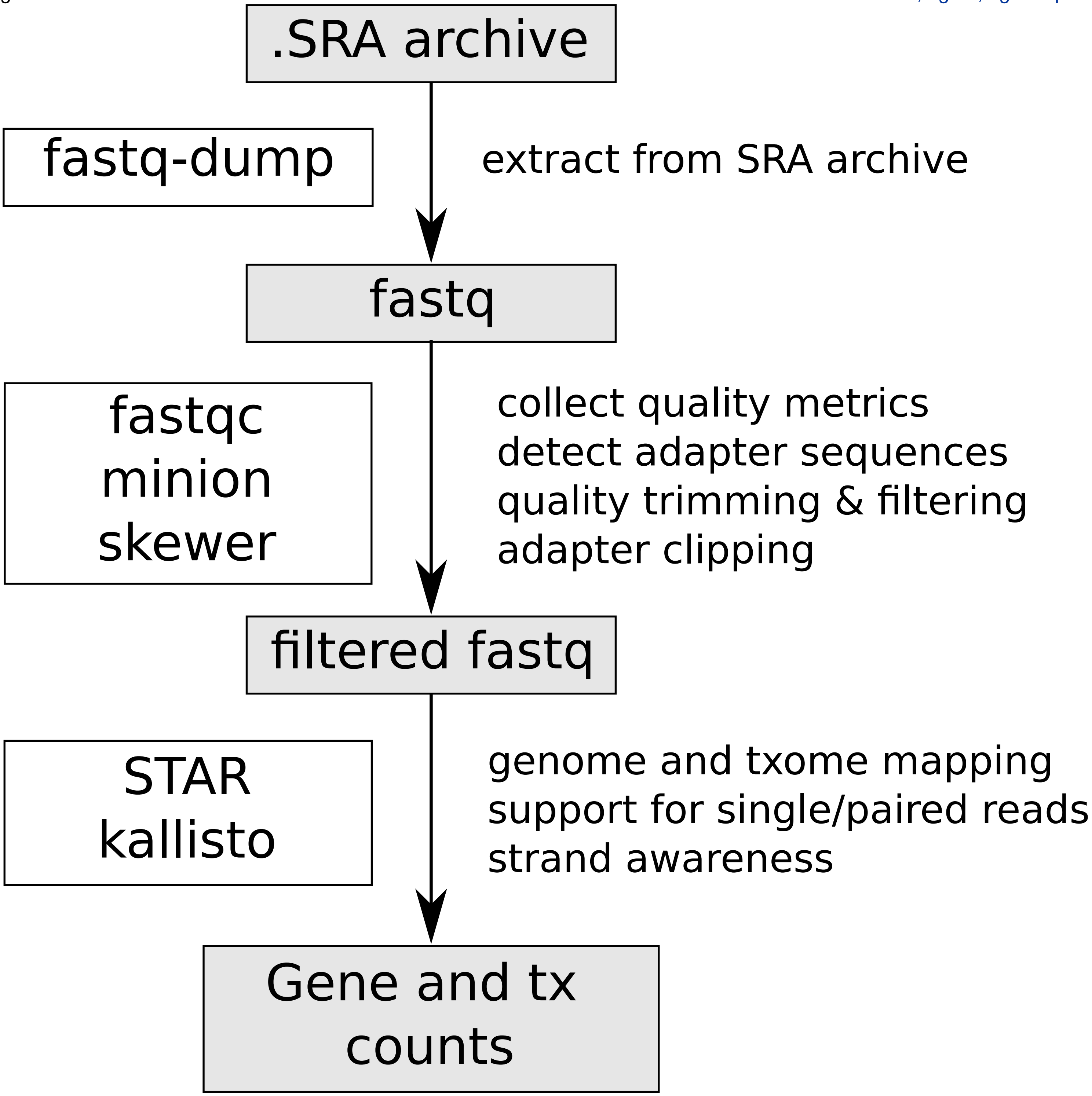

Figure 3

Click here to access/download;Figure;Figure3.pdf

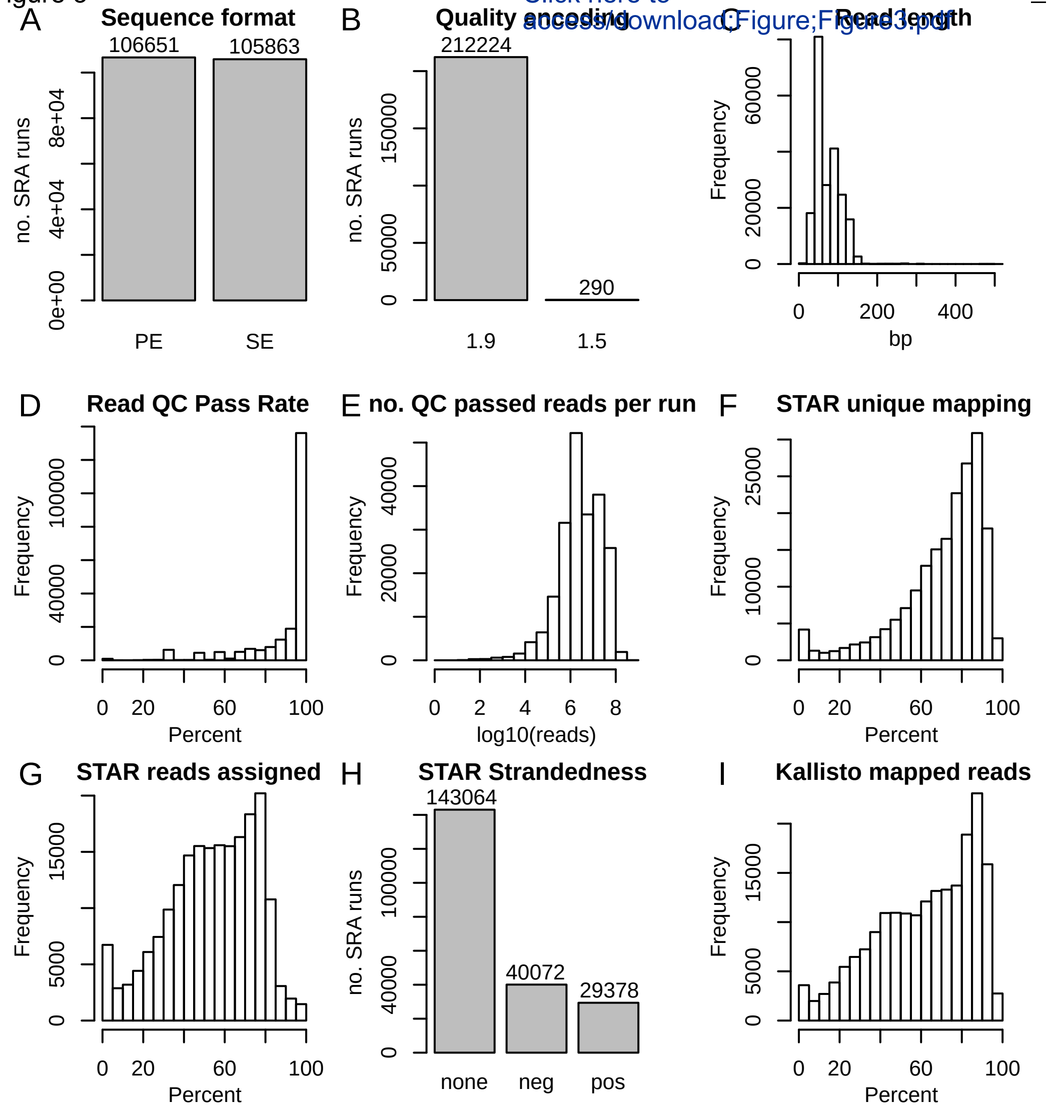

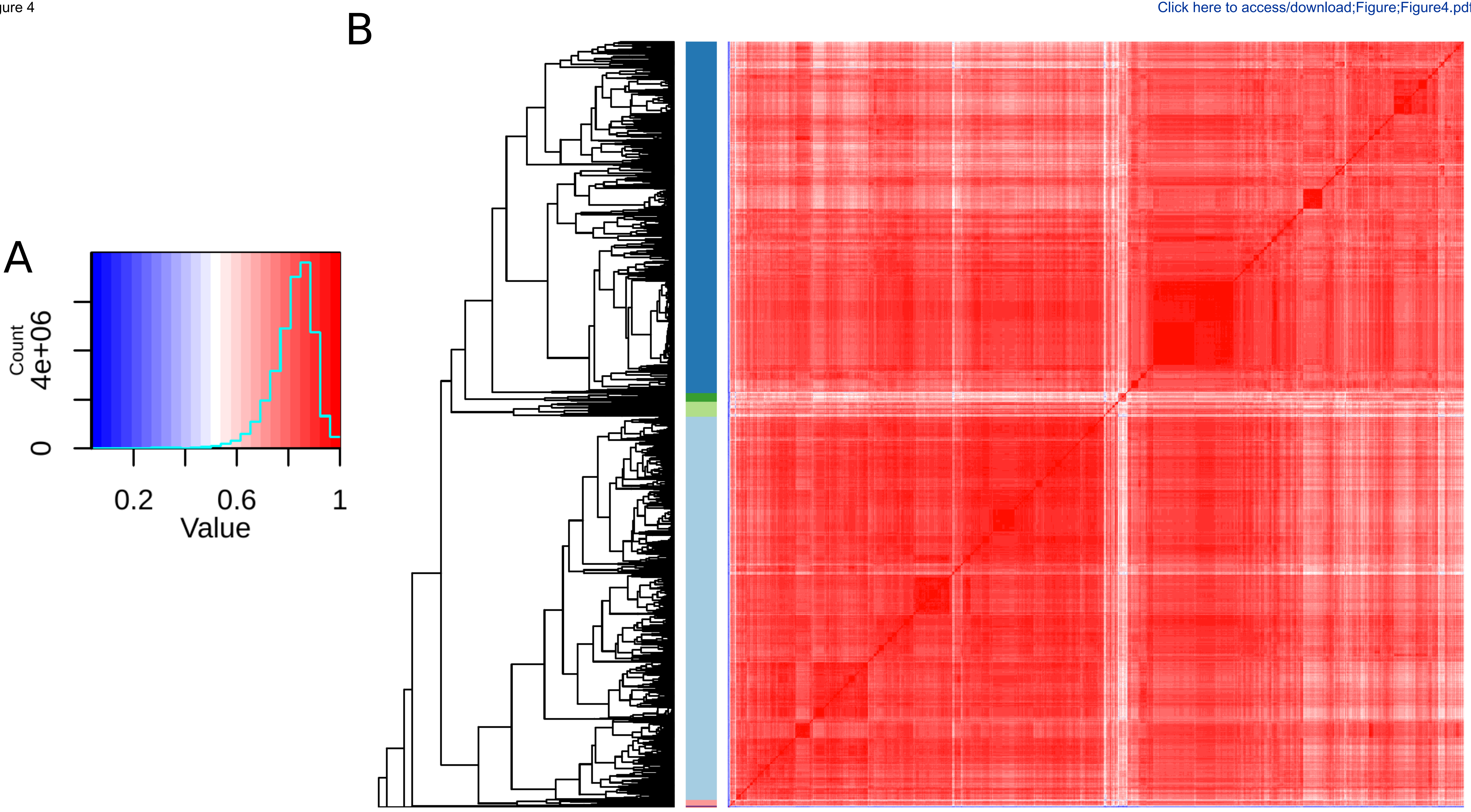
A
B
Count
4e+06
0
0.2
0.6
1
Value

Figure 5

Click here to access/download;Figure;Figure5.pdf

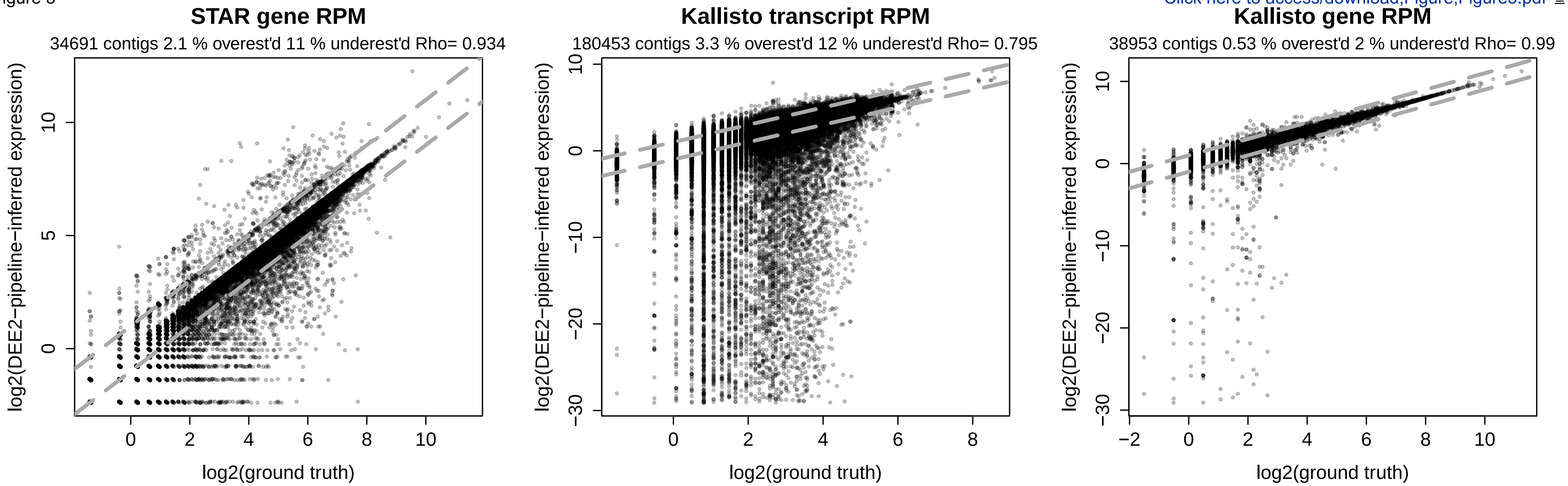

Figure 6

Click here to access/download;Figure;Figure6.pdf

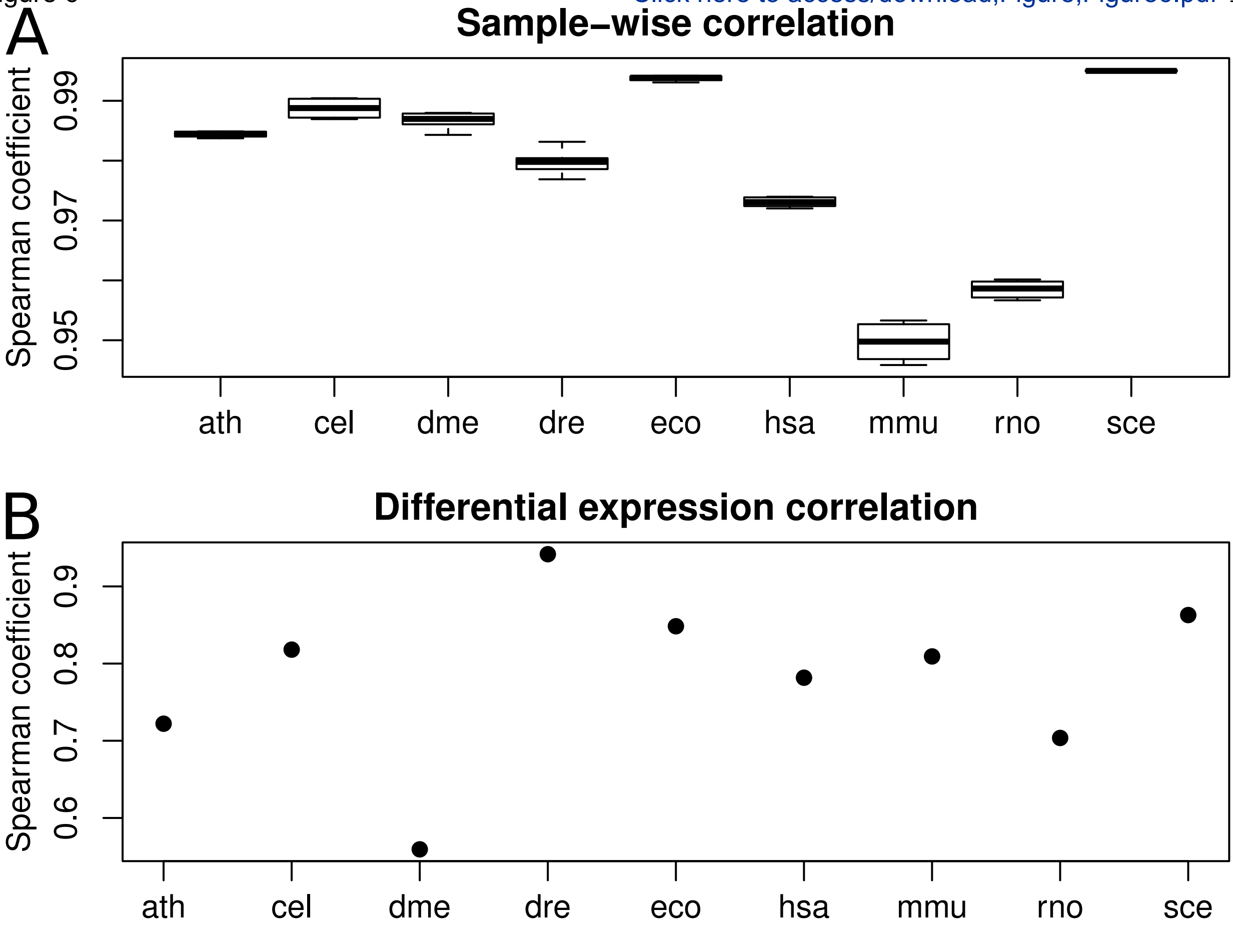

Figure 7

Click here to access/download;Figure;Figure7.pdf

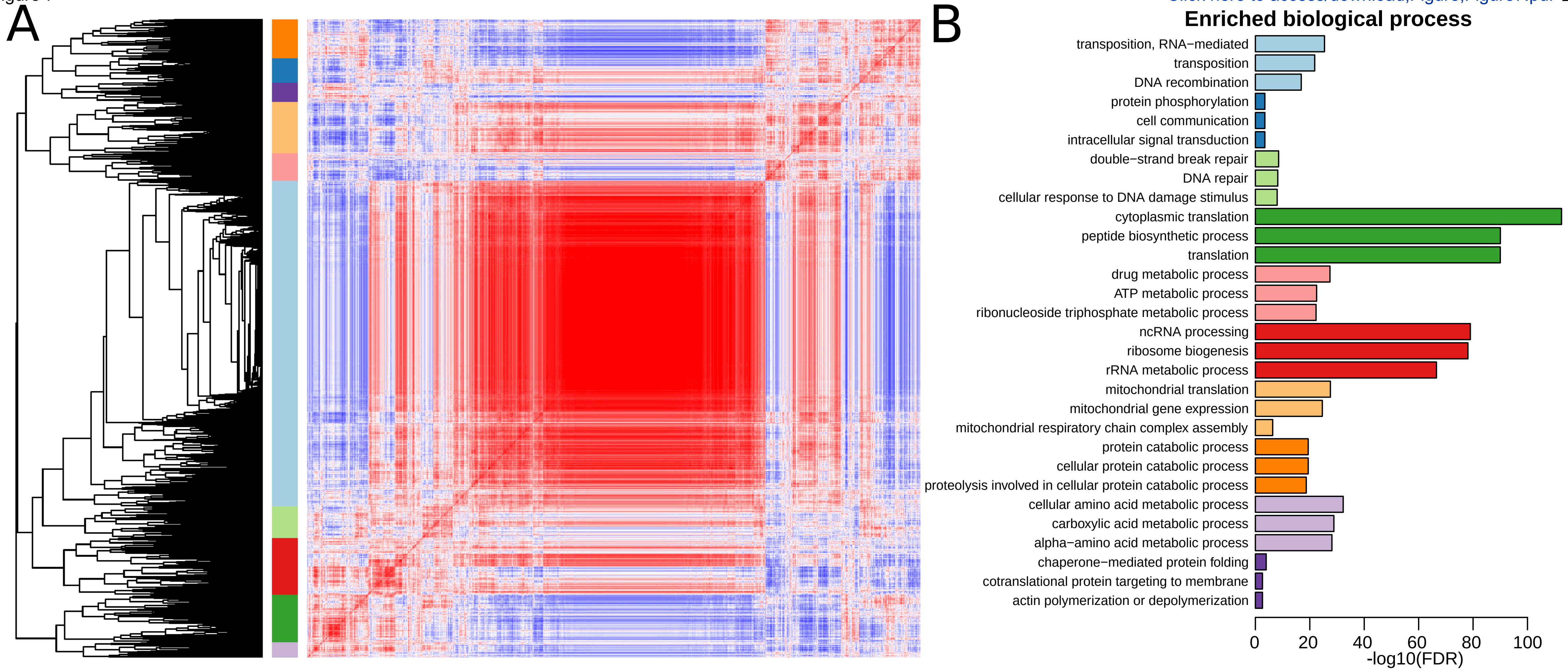

Figure 8

Click here to access/download;Figure;figure8.pdf

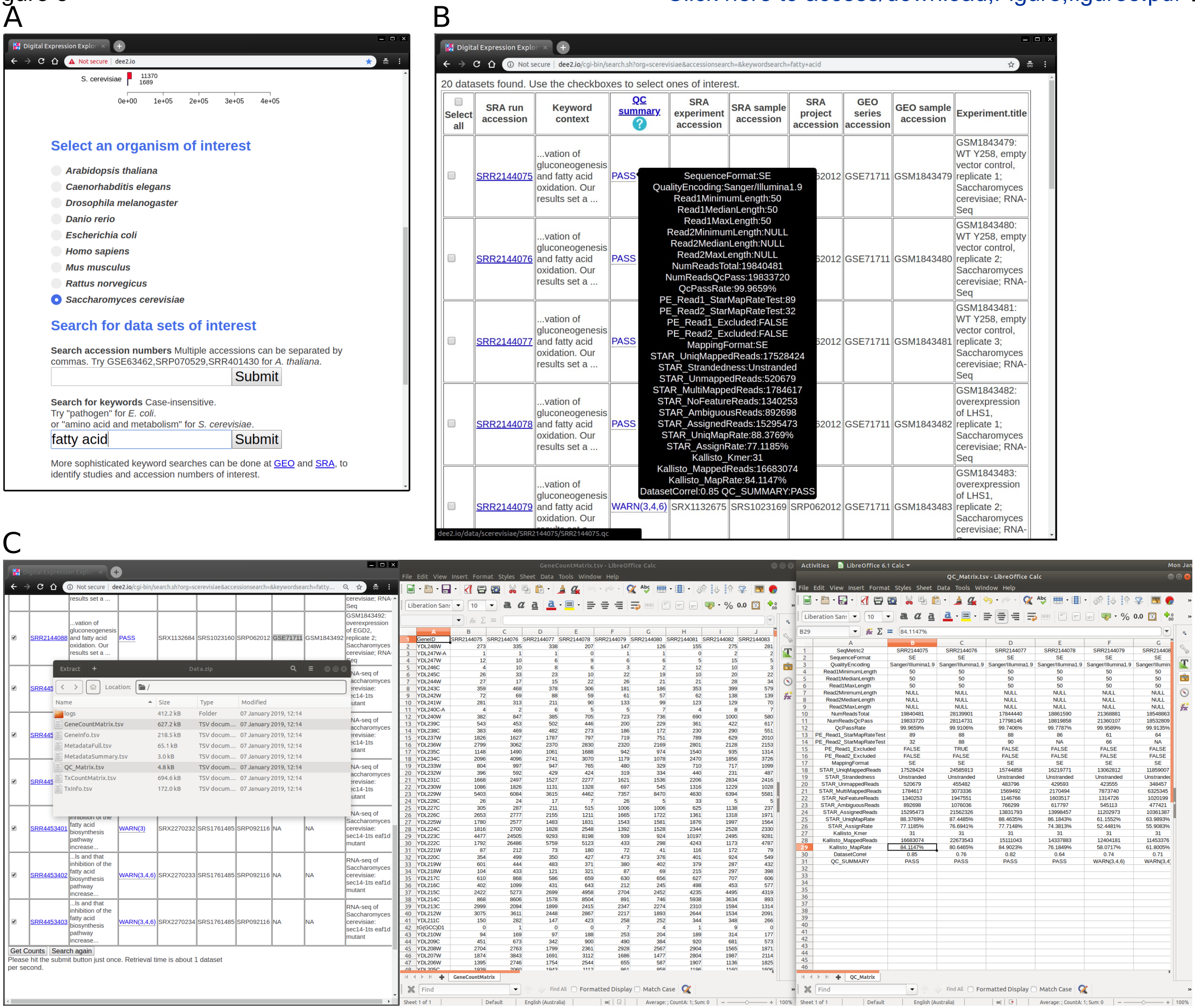

Personal Cover
Click here to access/download;Personal Cover;Response to Reviewer Comments.docx

Response to reviewer comments

We thank both reviewers for their thorough critique and highly constructive suggestions which have improved this body of work markedly. Below we have addressed each of the reviewers' points (#1 to #37). To make it clear what changes have been made to the manuscript we have provided a copy of the manuscript with changes annotated with numbered comments (#1 to #37) in addition to a "clean" version.
We have added SciCrunch RRIDs for the DEE2 resources: SCR_016931 Docker Image; SCR_016930 Source Code and SCR_016929 Project Homepage in accordance with the Editor's request.

Reviewer 1 Major points

1. The right side of Fig3C is cut off.

We have rectified this figure. All figures are now provided as separate pdf files in high resolution.

2. Page 8 : "in contrasts that had greater sequencing depth and more replicates. " in experiments ?

We have modified the passage for clarity:

"Differential expression correlation was higher in **comparisons** with more replicates (ρ=0.757, n=9, p=0.018). " Page 12.

3. I don't see the metadata ("Keyword context") in the downloaded tab files. This information is essential for processing, filtering and interpreting the downloaded data. Please include it. You may consider replacing the "Keyword context" field by "Accession description", which would make the selection of runs of interest easier. Also include this information in both the output of "Search accession numbers" in the webpage and the getDee2Metadata() function of the R API.

We have overhauled how DEE2 provides metadata and made the following modifications. Firstly the "Experiment.title" field from SRAdbV2 is now included in the search results. This gives the end user some idea about the samples without having to perform exhaustive searches of SRA/GEO. Secondly, the search results table is now downloaded as part of the results. In the zip package, it is called "MetadataSummary.tsv". In addition, all available information in SRAdbV2 for each selected run is provided in the file "MetadataFull.tsv". This feature is described on page 7 of the manuscript.

The R package now retrieves MetadataSummary data using the getDee2Metadata() function and the main function getDEE2() now returns both the metadata summary and the full metadata. This is demonstrated in the code example in Box 1 on pages 16-17.

4. Include a table with transcript and gene lengths so that FPKM type numbers can be calculated. and/or a the file or a link to download the gene annotation files that you use for each species.

This is a good suggestion. We have added direct links on the webpage (http://dee2.io/about.html) to the reference files that are used by DEE including the genome sequence, cDNA sequences and annotation sets (GTF).

We have calculated transcript length directly with a shell script and gene lengths were calculated using GTFtools (code included in the GitHub repo, with file name curate_gene_tx_info.sh).

Transcript lengths and gene lengths are now provided with each DEE2 download. This is mentioned in the manuscript text on pages 6-7.

The R script has been updated to make the use of this information, with the length of genes and transcripts found in the objects "GeneInfo" and "TxInfo" respectively. This should make FPKM calculations possible with just a few lines of code. This is illustrated on Box 1 on pages 16-17.

5. Searching with multiple keywords doesn't work. This has to be implemented. At least AND. This is a problem because the user can't download results if there are >500 matches. For example, if I search for the gene CLN3 in S cerevisiae I get 550 matches, and I can neither download all the data nor limit my search with AND, OR, or NOT keywords.

We have made several improvements to DEE2 searching to accommodate the cases where too many results are found. The keyword search is no longer limited to single words. Now phrases such as "amino acid deprivation" work as expected. "AND" is now supported, meaning searches such as "amino acid and fermentation" will work as expected. Users can chain together many keywords/phrases with ANDs to filter datasets as desired.

We have noticed a recent increase in the submission of SRA projects that contain many individual sequencing runs (hundreds to thousands). Case in point for the CLN3 search described by the reviewer which only brings up results for project SRP151525. In the manuscript we describe a feature where large datasets such as this are provided as "bundles" on page 14. We note that SRP151525 is indeed available as a bundle (http://dee2.io/bundles/scerevisiae/SRP151525_NA.zip)

6. Figure 5, and to a lesser extent, Fig 3, are very low resolution. Especially Figure 5.

We have rectified this figure (now Figure 8) using high resolution screen capture. Moreover, all figures are now provided as separate pdf files in high resolution.

7. The criteria used for quality control pass/fail are not clearly stated. For example, how was the "optimal number of bases to clip from the 5' end" calculated? Which read summarization method was used (union, intersection of all, the intersection of all non-empty)?

We have added greater detail to the "Pipeline features" section of the manuscript and outlined these below.

- Read level quality control is detailed on page 5 of the manuscript and is based upon (i) a phred threshold of 10, (ii) a minimum length of 18 nt, (iii) maximum allowable adapter frequency of 2.5%.
- "progressive clipping of 5' ends (4, 8, 12, 20 nt) followed by genomic mapping with STAR to determine the optimal number of bases to clip from the 5' end, as determined by the proportion of uniquely mapped reads."
- "STAR [18] is then used to map all reads that pass quality control (QC) to the genome and generate gene-wise expression counts with the "--quantMode GeneCounts" (no alignment files are generated)." Counts generated this way are identical to "union" (default) mode of htseq-count according to the author. (Reference: https://groups.google.com/d/msg/rna-star/gZRJx3ElRNo/Pq6bWLaJkpoJ)
- In a related point to this, summary QC metrics for human are now presented in Figure 3 (Page 8).

- Using these QC metrics, we have classified the datasets as “pass”, “warn” and “fail” according to some simple rules as summarised in the table below. Each rule has a numeric code. This is now discussed in the manuscript on page 8 and 9.

Metric	Meaning	Fail threshold	Warn threshold	Code
NumReadsQcPass	No. reads passed QC filtering	< 50 reads per gene	<500 reads per gene	1
QcPassRate	Proportion of reads passed QC filtering	< 60%	< 80%	2
STAR_UniqMapRate	Proportion of reads mapped uniquely to the reference genome using STAR	<50%	<70%	3
STAR_AssignRate	Proportion of reads assigned to genes with STAR	<40%	<60%	4
STAR_AssignedReads	No. reads assigned to genes with STAR	< 50 reads per gene	<500 reads per gene	5
Kallisto_MapRate	Proportion of reads assigned to transcripts with Kallisto	<40%	<60%	6
Kallisto_MappedReads	No. reads assigned to transcripts with Kallisto	< 50 reads per gene	<500 reads per gene	7
DatasetCorrel	Pearson correlation coefficient to passed data average	-	< 0.5	8

8. Figure 4: The legend is not clear enough. Why does 4A have error-bars and 4B does not? Is this because 4A includes all experiments (conditions and replicates), while 4B is the single differential expression value? I think 4A is a boxplot of correlation across all samples. But B shows a single value, even for GEOs that have multiple conditions, and therefore multiple differential expression values. Expand the text to make how these values were calculated more clear.

The reviewer has correctly concluded that panel A includes multiple runs and panel B looks at the correlation of a single differential expression contrast (it is now Figure 6). We agree that this section deserves more detail. We have added clarifying text to the “PIPELINE VALIDATION” section in page 11 and 12, as well as outlining precisely the methods used to perform this analysis in the methods section on page 19. Within the methods section, we provide Table 3, that gives additional details on the GEO series used in this analysis (page 20)

9. In Table S1, S. cerevisiae 50 bp PE has a much worse correlation (r = 0.46) than any other sample or species. What is the reason for this? This looks like a bug.

We thoroughly checked the code and identified two issues. Firstly there was a bug that meant that the wrong ground truth was being used for PE50 for all species (it was using SE50). After fixing this, results were improved for PE50 runs for all species.

Secondly, when not specifying a particular sequencing instrument, ART selects one based on the read length. The 50 bp sequences (SE and PE) were generated with a GAIIx error profile that was different to the 100 bp sequences which were according to HiSeq2000. To make the results more comparable at 50 and 100 bp, we have specified that error profile should match HiSeq2500 for all read sets. This resulted in improved results for SE50 and PE50 runs for all species.

The table (Supplementary Table 2 on page 29) has been updated in line with these changes.

10. The definition of each of the contents of the “QC summary” field in the web search results table should be provided somewhere. Similar definitions should be included in the R API.

We have defined each of the QC metrics in a markdown document on the GitHub page (https://github.com/markziemann/dee2/blob/master/qc/qc_metrics.md). This information is linked from the project webpage http://dee2.io/help.html as well as in the heading of the search results as shown below. In the R API, once a dataset is successfully loaded, a message is printed to the screen to help users find information on the QC metrics:

For more information about DEE2 QC metrics, visit
https://github.com/markziemann/dee2/blob/master/qc/qc_metrics.md

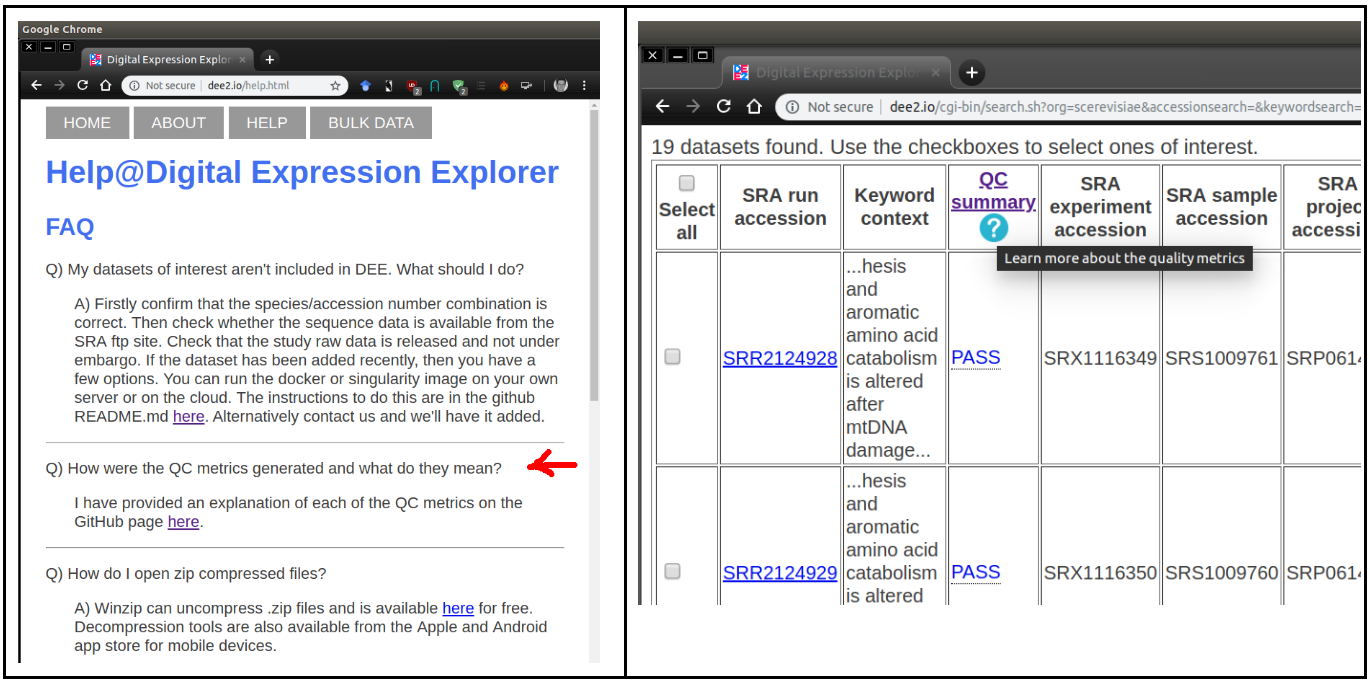

11. In the “Quality control and data validation” section, the authors describe the obtention of gene counts based on “Kallisto transcript counts collapsed into their parent gene”, which turn to be more accurate than STAR counts. However, these counts can’t be obtained through the web or the R API. The reason for this should be clarified, and the method to reproduce this result available. The user should be able to download the gene-transcript table. Was the calculation simply the sum of counts from all transcripts that ‘belong’ to a given gene? Or something more complicated?

This is a very good suggestion. As noted above, DEE2 now provides TxInfo.tsv file in each download that contains the relationships between transcript and parent genes.

The getDEE R API script contains a new function called “Tx2Gene” which performs aggregation (sum) of transcript counts to gene level counts with a single command.

> x<-Tx2Gene(x)

Here is a demonstration of downloading counts and aggregating.

```
x<-getDEE2("celegans",c("SRR363796","SRR363797","SRR363798","SRR363799" ))
trying URL 'http://dee2.io/metadata/celegans_metadata.tsv.cut'
Content type 'text/tab-separated-values' length 481328 bytes (470 KB)
==================================================
downloaded 470 KB

trying URL 'http://dee2.io/cgi-
bin/request.sh?org=celegans&x=SRR363796&x=SRR363797&x=SRR363798&x=SRR363799'
downloaded 1.2 MB

> names(x)
[1] "GeneCounts"      "TxCounts"        "GeneInfo"        "TxInfo"
[5] "QcMx"            "MetadataSummary" "MetadataFull"    "absent"
> head(x$TxInfo)
                   GeneID GeneSymbol TxLength
Y110A7A.10 WBGene00000001      aap-1     1787
F27C8.1    WBGene00000002      aat-1     1940
F07C3.7    WBGene00000003      aat-2     1728
F52H2.2a   WBGene00000004      aat-3     1739
F52H2.2b   WBGene00000004      aat-3     1840
T13A10.10a WBGene00000005      aat-4     1734
> x<-Tx2Gene(x)
> names(x)
[1] "Tx2Gene"         "GeneCounts"      "TxCounts"        "GeneInfo"
[5] "TxInfo"          "QcMx"            "MetadataSummary" "MetadataFull"
[9] "absent"
> head(x$Tx2Gene)
               SRR363796 SRR363797 SRR363798 SRR363799
WBGene00000001        11        23        48        45
WBGene00000002         0         0         0         0
WBGene00000003         0         2         0        21
WBGene00000004         0         4         7         5
WBGene00000005         0         0         0         0
WBGene00000006         2         1         0         2
```

The manuscript has been amended to make note of these features on page 15-17. This has been added to the documentation on GitHub (AccessDEEfromR.md) and the project website (http://dee2.io/help.html)

Moreover, additional detail on how the simulation study was performed are provided in the methods on page 18.

12. The accession SRA355722 does not give any results for *A. thaliana* in the web server even though it is provided as an example on the website. The authors should make sure that all the website examples work.

The example on the homepage has been amended from SRA355722 to SRP070529.

13. In the figure 3 legend, it is unclear what ART means. Adding a citation will make this clear.

A citation has been added to the legend (now Figure 5). Furthermore to address some of the uncertainty around this, we have provided a methods section "Pipeline validation using simulated data" on Page 18.

14. The acronyms UMI and QC should be defined when they're used the first time.

We have included the full terms at the first instance (last paragraph of page 5).

Reviewer 1 Minor and optional points:

15. "the datasets will be available publicly. In this way, power users obtain benefit by using an established analysis pipeline and simultaneously contribute..." I think this is VERY clever, and could be highlighted more. Other projects could learn from this.

Thank you. We think the current emphasis is appropriate.

16. Mouse-over on QC doesn't work if you have >500 matches. This is confusing and should be fixed or just remove the mention of mouse-over in the text.

The mouse-over QC info is slow load when there are >500 hits and therefore it is limited. This has been clarified in the manuscript on page 14.

17. When a search returns >500 matches, an option to download the first 500 would be nice.

To address this, we have made some changes to the search functionality in the web browser. When between 501 and 5000 matches are obtained, the user now has an option to download the metadata as a TSV file. Users can then run smaller queries, for example, using SRA project accession numbers. This feature is mentioned in the manuscript on page 14.

18. With regards to quality control: It would be very useful to be able to filter by minimum correlation with the bulk of the experiments for a given species. In our hands, with DEEv1, most S cerevisiae experiments had an inter-experiment correlation > 0.5; these appeared to be "normal" mRNA-seq experiments according to the metadata. A subset of experiments (~10%) have very low correlation with any other experiment, and are probably garbage. This might be a nice quality control metric to include in the search.

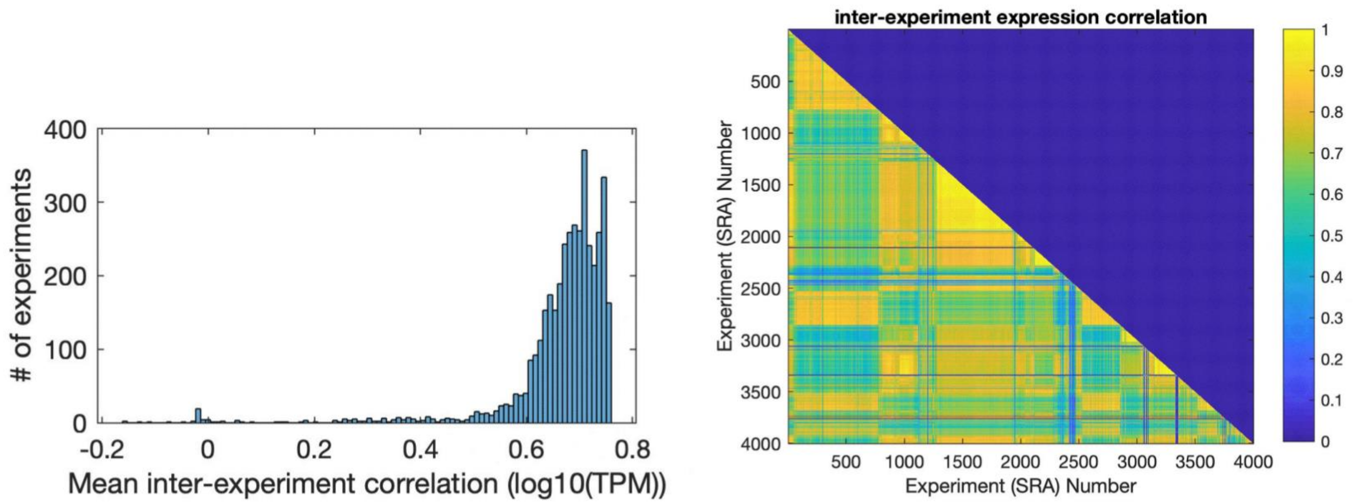

In line with this suggestion we have made major changes to the curation scripts which I will outline.

The type of all-to-all correlation analysis shown by the reviewer may take only a few minutes with *S. cerevisiae* data but would be very computationally intensive to be regularly recalculated for human or mouse data. To make this problem tractable, we randomly selected up to 10,000 datasets that passed all other QC criteria and generated an "average" of these datasets. Then, all datasets were compared to this "average", generating Pearson correlation coefficients. The histogram of correlation coefficients was nearly identical to the reviewers own (left panel above).

The “average” profile will be updated periodically and so the correlation coefficients may vary slightly over time. This is why they are reported to two significant figures only.

These correlation coefficients are now part of the QC information that are visible in DEE2 search results as well as part of the QC table downloaded by the R API (screen grab and R output shown below). If a dataset has a correlation coefficient <0.5, then it is flagged as a warning.

These changes will inform end users of the correlation to the bulk of datasets and allow them to perform filtering. In the manuscript, this feature is described on page 9.

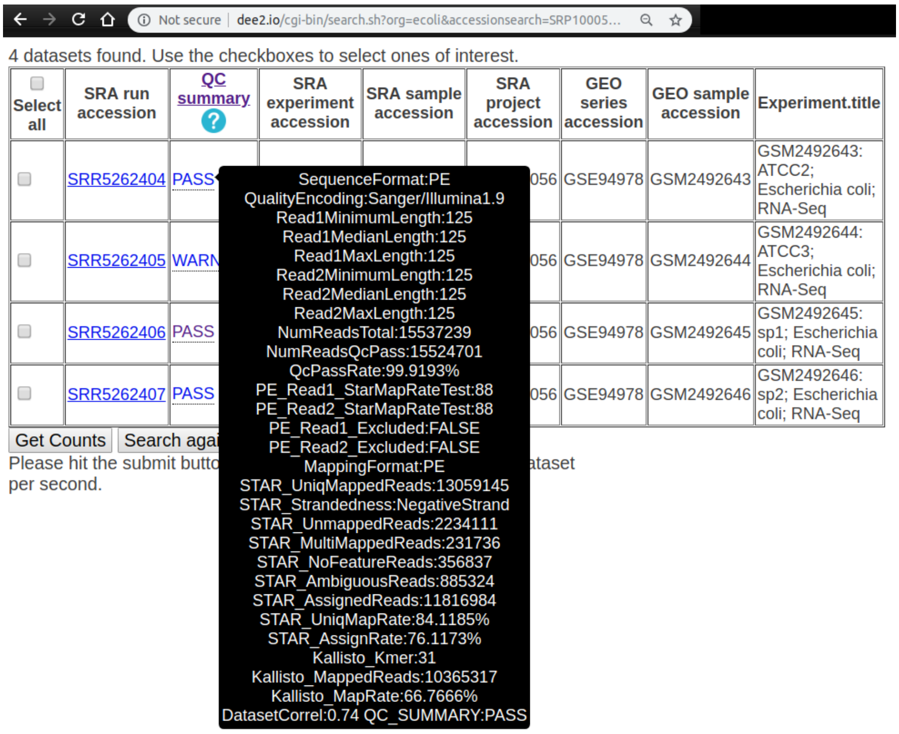


```
> x<-getDEE2("ecoli",c("SRR5262404","SRR5262405","SRR5262406","SRR5262407"))
trying URL 'http://dee2.io/metadata/ecoli_metadata.tsv.cut'
Content type 'text/tab-separated-values' length 199956 bytes (195 KB)
==================================================
downloaded 195 KB

trying URL 'http://dee2.io/cgi-
bin/request.sh?org=ecoli&x=SRR5262404&x=SRR5262405&x=SRR5262406&x=SRR5262407'
downloaded 221 KB

For more information about DEE2 QC metrics, visit
https://github.com/markziemann/dee2/blob/master/qc/qc_metrics.md
> tail(x$QcMx)
                     SRR5262404 SRR5262405 SRR5262406 SRR5262407
STAR_AssignRate        76.1173%   56.1098%   81.6333%   81.0047%
Kallisto_Kmer                31         31         31         31
Kallisto_MappedReads   10365317   15276751    9620960   11035083
Kallisto_MapRate       66.7666%   71.7846%    61.871%   64.3676%
DatasetCorrel              0.74       0.75       0.59       0.62
QC_SUMMARY                 PASS   WARN(3,4)      PASS       PASS
```

19. It would be very interesting, and therefore possibly increase usage, to use the DEE to make one or two points. For example, why do some experiments have such low correlation with the bulk of the data (see above)? Are they not really mRNA-seq? Different method? Not from this species?

We performed a correlation analysis of DEE2-STAR data to understand the source of variability in datasets as well as genes. In *S. cerevisiae* we observed that there a small fraction of datasets with poor correlation to the “average”, consistent with the reviewer’s observation. An unsupervised

clustering analysis of the 5807 dataset correlation showed 5571 datasets belonged to two large and highly correlated clusters (blue and light blue in the heatmap below). The remaining 236 datasets belonged to several smaller clusters.

Cluster 3 (yellow, n=111) contains datasets mostly derived from non-standard RNA-seq such as 3' end RNA sequencing (ERP004367, SRP048715, SRP048715, SRP021938) and Ribo-Seq (SRP075766, SRP082147). Cluster 4 (light green, n=65) also contains datasets mostly from non-standard RNA-seq approaches including RNA-IP-Seq (SRP032276), Ribo-Zero (SRP057417), or from samples undergoing sporulation and meiosis (SRP092588,SRP061166, SRP032309). Cluster 5 (dark green=45) contains datasets derived from spike-in experiments (SRP052299, SRP069877, SRP142613). Cluster 6 (n=6) contains 6 datasets from project (SRP061165) which uses a targeted sequencing approach to look only at a small number of genes. Clusters 7 to 9 each contain one dataset. Cluster 10 (pink) contains 6 datasets from SRP131449, a directed evolution study.

From this analysis, we can conclude that the highly correlated datasets are standard RNA-seq/mRNA-seq and the datasets with low correlation to the bulk are mostly due to the use of non-standard library construction protocols, but also due to the presence of different biological states (sporulation/meiosis as opposed to growth phase).

Explanation to this effect has been added to the manuscript on page 9 and 10, and the clustered heatmap is incorporated as Figure 4. Methods describing this analysis have been added to page 16 and 17.

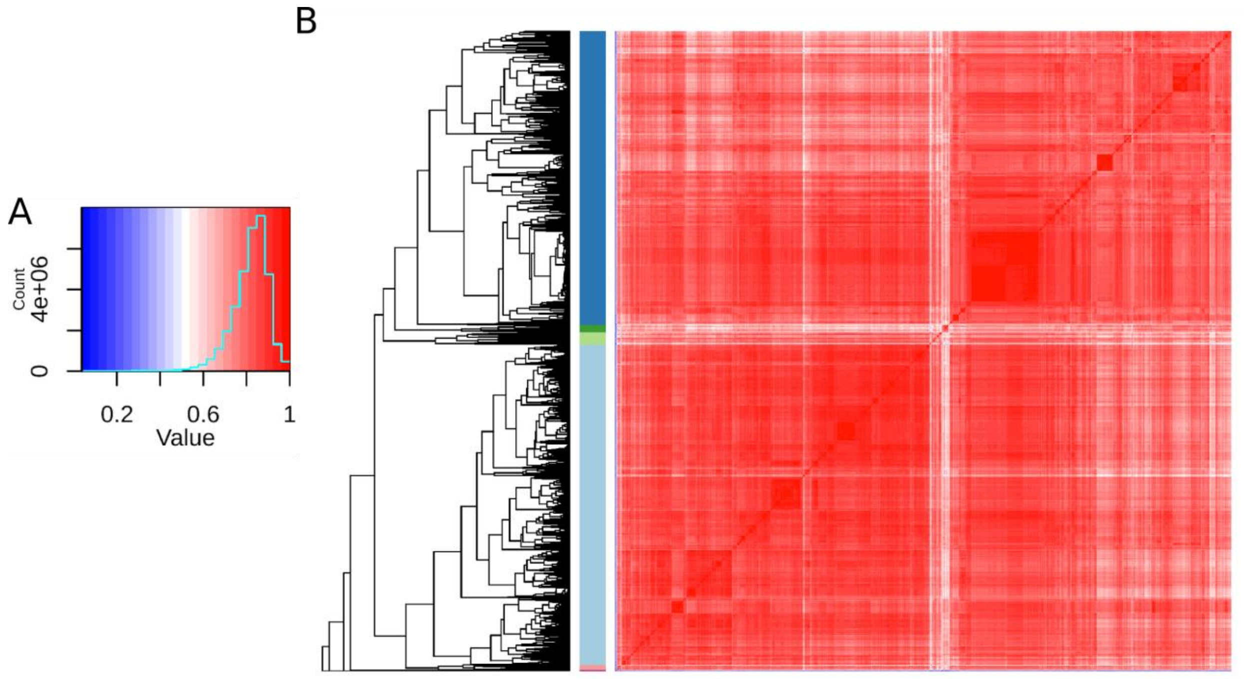

Figure 4. Unsupervised clustering analysis of the correlation of 5807 S. cerevisiae datasets. (A) Colour key and histogram of Spearman correlation coefficients. (B) Heatmap of pairwise correlation values with datasets clustered by similarity.

With regards to the question whether the datasets are really the organism of interest, we have implemented strict control of this in the pipeline as we are aware that SRAdb/SRAdbV2 contains a small number of errors. Therefore before downloading and processing any dataset, the pipeline confirms the species name at SRA (https://www.ncbi.nlm.nih.gov/sra) matches what is stated in

SRAdbV2. We confirm that this is working, as for example, SRP032309 contains data from normal *S. cerevisiae* and *S. paradoxus*, an interspecies hybrid, but only the *S. cerevisiae* data is present in DEE2.

20. Page 3, line 1: In the sentence "RNA-seq has become as a powerful method in transcriptomics", "as" should be removed.

This typographical errors has been corrected. (First line of Background section, page 3).

21. The "Figure 1" text in the lower left corner of Figure 1 is redundant and may be removed.

Figure 1 and 2 have had the lower left label removed.

22. Figure 3 would benefit from a larger font size in the axis labels. In addition, figure 3 plots would be easier to interpret with a density scatterplot, which would allow to see where the majority of the genes/transcripts are. There's no need to separate the >2 fold and <2 fold regions with colors, as dashed lines could serve the same purpose. Would also be nice to see the % of genes/transcripts in each category.

Axis labels have been enlarged (now Figure 5, page 11). In addition, we generated kernel density plots (below left), with an example below. However we believe that the scatterplots better show the relatively small number of contigs that differ from the expected line. The problem of having many points hidden in the denser areas is overcome by reducing the opacity of each point, as demonstrated in the modified scatterplot style shown below. Dashed lines showing the >2 fold and <2 fold are helpful to the reader.

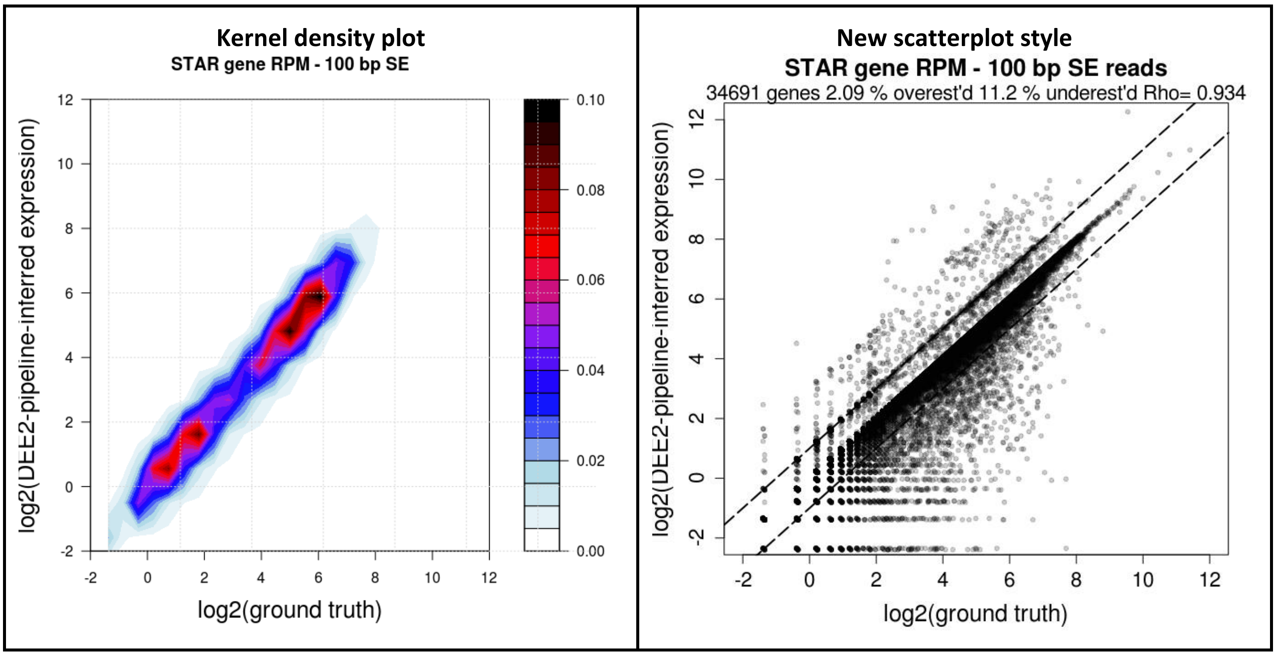

23. In figure 3, log 2 (simulated) values (x axis) are the expected expression values. All data are simulated. The figures would be more clear having the "log 2 (expected expression)" as X axis and "log 2 (DEE2-pipeline-inferred expression)" as Y axis.

In line with the reviewer's comment, we have amended the axes labels (now Figure 5), as shown in our response to point #22 above.

24. The sentence "Correlation was higher in contrasts that had greater sequencing depth and more replicates" should be supported with data.

We correlated the DE Spearman rho with the number of replicates in each contrast and found a positive correlation (rho=0.757) for the 9 data points. This indicates that the two quantities are correlated (p=0.018). This information has been added to the text on page 12.

25. It is unclear how were the Figure 4 datasets chosen. How do we know that they did not just picked the best experiments of each species? I encourage the authors to use multiple experiments per species, randomly selected from datasets with pre-calculated expression values. This will eventually make the argument that "Both validation exercises demonstrate the accuracy of DEE2 data".

The project datasets described in the figure (now Figure 6) were selected manually by searching through GEO for datasets that satisfy a number of criteria:

- Provide raw gene expression counts for each run. Many other datasets only provided transcript-wise data or TPM, FPKM or only post-DE data (logFC+FDR).
- Provide either Ensembl gene names or official gene symbols. Entrez IDs and other gene identifiers are more difficult to work with.
- Have 2 or more replicates per group (more is always better).
- Acceptable quality metrics including good number of reads passing QC for the complexity of the transcriptome and genomic mapping rate.

These criteria have been added to the manuscript on page 19. Given the above restrictions, only a very small fraction of author supplied datasets in GEO are compatible. The result of this exercise shows that DEE2 provided counts are highly similar to those published in well respected journals, despite differences in analytical pipeline and reference genome information (Table 3 captures this information). We also show that the results of differential expression are highly similar, even when the number of replicates varies between two and eight. Given the manual and time-consuming nature of finding GEO series that satisfy the above criteria, we believe the addition of multiple GEO series for each species is not justified.

Reviewer 2 Major points

26. I was not able to find any information about the specific software versions or parameters that were used in data re-processing, but these represent critical pieces of information with respect to reproducibility. The DEE2 webpage provides only the reference genome sequence and annotation versions (http://dee2.io/about.html), and links to the GitHub repo (https://github.com/markziemann/dee2) for details on the processing pipeline; from what I can tell, this repo contains the source scripts for the pipeline (from which one can thus find the parameters used for each method), but versions are not included here. From this, I was able to figure out that, for example, STAR was run with --quantMode GeneCounts to obtain gene-level counts; however, this information should be much more obvious and ideally should be included on the website or in the output files themselves. As a related note, perhaps DEE2 should itself also be versioned for reference so that results can easily be referenced if annotation/software versions are updated.

We agree with the reviewer that software version numbers are important to the reproducibility of the work. We now provide software versions, purpose and parameters in Supplementary Table 1 and this information is also provided on the project webpage (http://dee2.io/about.html).

We also agree that versioning is critically important. This is DEE v2.0. Since data processing has been begun the processing method has remained consistent, but we have added new features to enhance the UI. When we eventually change the data processing method, we will be permanently archiving previous versions for bulk download only.

27. Some relevant details are occasionally missing from the paper. For instance, are the kallisto transcript values the TPM values (i.e. normalized by genomic feature effective lengths?) or the est_counts values? This detail was unclear to me. Similarly, on page 7 line 1, the authors discuss generating "synthetic Illumina HiSeq RNA-seq data", but don't mention the tool they use for this purpose (ART) except in the caption for Figure 3, leading to some confusion for the reader. Some minimal details about their simulation (number of genes, read length, coverage etc.) should be included, at least in Supplementary Materials.

We have now provided greater detail with regard to the methods used. On page 6 we explicitly describe that DEE2 provides estimated counts (est_counts). With regard to the validation of the pipeline, we provide a methods section on page 18 that describes the parameters that were used to generate simulated RNA-seq reads. Quoted below:

"Pipeline validation using simulated data. To validate the accuracy of the DEE2 pipeline, we generated Illumina HiSeq2500-like sequence reads from Ensembl cDNA sequences using ART (v2016-06-05)[23] with a defined seed (1540165885) and a uniform fold coverage of 2. Read lengths were 50 and 100 bp in single and paired end format. The read sets were processed with the DEE2 pipeline and the observed expression data were compared to the ground truth. These analyses were performed for all nine organisms currently included in the DEE2 dataset."

28. Unless I've misunderstood, there would be no way to read in the kallisto transcript estimates using sleuth or tximport (which are both state-of-the-art tools for importing and summarizing transcript estimates to gene-level), as the original format of these files was not retained (i.e., they are now wide matrices containing only estimated transcript counts, rather than long matrices including length, eff_length, est_counts, tpm). All of the documentation appears to focus on the use of the gene counts; do the authors have another intended downstream use for the kallisto transcript estimates?

In response to this question and #11 above, we have incorporated a new function in the R package to aggregate transcript counts to gene level by summing counts from transcript isoforms to the parent gene. This is now documented in the manuscript on page 15-17, in the documentation (https://github.com/markziemann/dee2/blob/master/AccessDEEfromR.md) and project webpage (http://dee2.io/help.html).

If users are after other information from Kallisto processing such as TPM values, these are available for download (eg: http://dee2.io/data/dmelanogaster/SRR1991949/SRR1991949.ke.tsv.gz), and is now documented on the projects help page (http://dee2.io/help.html). These files are compatible with tximport. Moreover, as outlined at point #4 above, we now supply transcript lengths so that the end-user can more easily perform FPKM calculations.

29. In the previous iteration of this tool, there was a SraRunTable text file with sample-meta data that was also downloaded with count matrices. This information is obviously critical in order to account for batch effects and appropriately model the experimental design in downstream analyses. I greatly appreciate the easy integration of DEE2 with the SRAdbV2 interface for NCBI SRA metadata, and this will be useful for re-analysis of data from within R. Is there any similarly easy way for a non-R user to

obtain these tables (downloaded data from the website only include gene counts, transcript counts, QC, and related logs)?

As requested by the reviewer, now the metadata are provided alongside the expression tables in the zip download. MetadataFull.tsv contains all available corresponding dataset information from SRAdbV2 and MetadataSummary.tsv contains only the corresponding SRA accession numbers and "Experiment.title" which should provide enough information for the end-user to perform downstream analyses. This feature is described on page 7. The bulk data page also now contains summary metadata (celegans_accessions.tsv.bz2).

30. Is there any intention to add other species? I particularly have in mind agriculturally relevant species such as chicken, pigs, and cattle, as I think such a resource could be very valuable for researchers in the animal genetics community. Also, given the large availability of human/mouse reprocessed data in other large-scale data compendia, is there any reason that so many DEE2 resources have been dedicated to re-analyzing these species (5787+5737 = 78% of the DEE2 datasets) rather than focusing resources on other species?

This is something we are considering doing in future but not in the scope of the present study. Upon publication we will be in a stronger position to request funding to expand the number of species and added features.

On the point of overemphasis of human and mouse datasets, we feel strongly that DEE2 provides some strengths that other resources lack. For example, features such as smart 3' adapter clipping and use of both STAR and Kallisto mean that derivative RNA-seq method datasets such as small-RNA-seq are well represented. A keyword search for "microRNA" in *R. norvegicus* shows the top two hits SRR094777 and SRR087427 are small RNA datasets and the most abundant species are microRNAs.

GeneID	SRR087427	SRR094777
ENSRNOG00000035603	899828	632367
ENSRNOG00000035451	530775	37305
ENSRNOG00000035636	333974	750377
ENSRNOG00000035611	161777	263067
ENSRNOG00000035535	155140	78372
ENSRNOG00000035554	142199	132994
ENSRNOG00000057048	137508	438974
ENSRNOG00000035646	119285	291381
ENSRNOG00000035480	89223	85504

Reviewer 2 Minor points

31. I think it could be useful to cite BgeeDB (https://bgee.org; https://bioconductor.org/packages/release/bioc/html/BgeeDB.html) and briefly situate that work with respect to the current work. Like the current work, Bgee includes a variety of species other than human and mouse; however, unlike the current work, it instead focuses on normalized data arising from a variety of platforms in multiple tissues/life stages of curated normal samples to facilitate cross-species and cross-tissue analyses.

In the background section on page 3 of the manuscript, we have described and cited BgeeDB as a curator of high quality baseline expression data.

32. Does Supplementary Table 1 represent the median Spearman correlation coefficients across simulated genes? And are these correlations (as well as those in Figure 4A) based on counts, or log-counts? In Figure 4B, what is plotted? Median (across all genes) Spearman correlation between estimated log-fold changes for DEE2-STAR and author-supplied counts for a single chosen dataset? Why not use boxplots for these as well?

The supplementary table shows Spearman correlation coefficients (ρ) between ground truth and DEE2 processed expression profiles (RPM) from simulated data. The title of the table has been updated for clarity. These correlation coefficients are based on RPM values, although being a non-parametric correlation analysis, these coefficient values would remain the same if we chose to use raw counts, RPM, log-counts or log-RPM values, unlike Pearson correlation analysis.

Figure 6 (formerly Figure 4) is not simulated data, rather public RNA-seq data. Figure 6A shows the correlation of author submitted data with DEE2 processed data at the level of individual runs. If there are 6 samples in the GEO series (Like GSE46344 for *C. elegans*) then the box plot in Figure 6A summarises 6 correlations. Figure 6B shows the correlation of gene ranking after differential analysis (all genes). Extra details have been added to the "PIPELINE VALIDATION" section to make this explicit (page 11 and 12). Furthermore, additional details on the analytical methods datasets used are included in the "Pipeline validation using public data" subsection of the methods section on page 19. The data in Figure 6B are shown as individual points as two differential analyses can be compared using Spearman correlation (with the ranking dictated by the sign of the fold change and p-value). The steps undertaken to perform these analysis are now precisely described in the methods section on page 19. Table 3 also includes further information as to the origin of the public datasets used in this analysis (page 20).

33. Are there datasets for which the QC summary is WARNING or FAIL instead of PASS (and if so, do you have general statistics on the number of passing/failing datasets)? After a quick browse through some of the species, they all appeared to be flagged as PASS. Does an experiment get flagged as FAIL if a single QC metric fails (or in other words, what is the threshold for flagging an experiment as failing QC)? In any case, I like that all of the statistics are available to the user, allowing each person to decide whether to include a dataset or not.

Due to a lack of definitive thresholds for pass/fail classification in the literature, we deferred making judgements on what constitutes pass or fail. Based on our prior experience with RNA-seq data and analysis of DEE2 QC metrics (eg: Figure 3), we formulated heuristics to classify datasets as "PASS", "WARN" and "FAIL" (Table 2, reproduced at point #7 above). All datasets are still provided on the webserver as they may still be useful despite being flagged as potentially poor quality. This classification procedure is outlined on page 8 and 9. The number of pass/warn/fail datasets is now given in Table 1.

Reviewer 2 Optional points

34. One minor point that I hesitate to mention (given the large amount of work the authors have already put in), but that could significantly strengthen the paper: a small example to illustrate a meta-analysis using processed DEE2 data would be wonderful to illustrate the ease and benefit of the DEE2 compendium.

As part of addressing #19 above, we performed a correlation analysis of all datasets in yeast. This type of approach could also be used to identify clusters of co-regulated genes just by transposing

the matrix prior to correlation analysis. After performing this analysis, a heatmap was generated, showing a large block of correlated genes and several smaller clusters, each with their unique profiles. GO analysis of these clusters revealed that they appear to have distinct biochemical specialisations. The relatedness of these clusters is interesting in that protein translation (dark green) is correlated with amino acid metabolism (light purple), while the mitochondrial cluster (light orange) was correlated with ATP metabolism (pink). These findings provide but a taste of the type of meta-analyses that are possible using DEE2 data (page 12-13 of the manuscript).

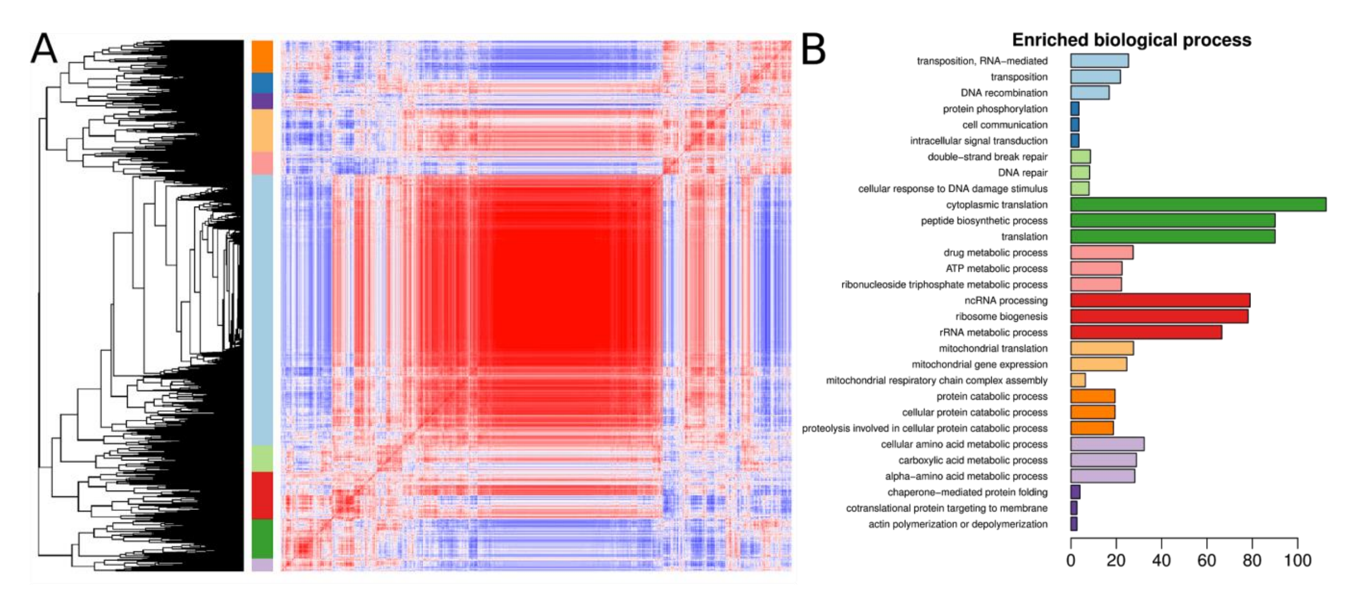

Figure 7. Unsupervised hierarchical clustering of gene expression in *S. cerevisiae*. (A) Clustering and correlation heatmap. (B) Biological process gene ontology enrichments of each cluster.

35. Sourcing an R script such as getDEE2.R from the web is a fairly insecure practice; see https://www.jumpingrivers.com/blog/security-r-hacking-bioconductor for a blog discussing this. After having used a source command for package installation for many years, Bioconductor has actually recently switched to the new BiocManager package (https://cran.r-project.org/web/packages/BiocManager/vignettes/BiocManager.html) as a safer way to install and manage Bioconductor packages. I wonder whether it would be worth bundling your R access scripts into a minimal package on GitHub that could then in turn be installed using install_github() from devtools?

Thank you for informing us of the possible danger of sourcing code over the web. We have added some documentation to the R script to create a package that can be installed via github. This is mentioned in the abstract on page 2 and on page 15. The package can be installed using R devtools.

```
library("devtools")

devtools::install_github("markziemann/dee2/getDEE2")

library("getDEE2")
```

36. There are some small inconsistencies in the explanation page at https://github.com/markziemann/dee2/blob/master/AccessDEEfromR.md. For example, the metadata are loaded into an object called 'mdat', and the subsequence line of code looks at the head

of 'metadata'. This could be rectified if the document was instead written as a reproducible Rmd document.

Thank you for highlighting the error. We have corrected it.

37. The authors discuss the financial cost of regenerating these experimental data in the "Re-use potential" section. It would also be interesting, if possible, to break down what the total computational cost (in terms of $$ or time) of creating the DEE2 compendium is/was. This could help provide an additional selling point for the great value of making available these re-processed data, thus allowing researchers to avoid unnecessarily re-doing these same steps themselves.

To address this point, We recorded the progress of two 16 thread servers processing human datasets from the queue. The specs of these Nectar servers are as follows: Intel Xeon E3-12xx v2 (Ivy Bridge, IBRS) 2.6 GHz, 64 GB RAM, ~400 MB/s download speed from NCBI. For the runs that finished successfully, the results for the two servers were similar, with a median elapsed time of 833 s (n=379) and 961 s (n=369) per SRA run. The results of the 16 thread servers are plotted below. On a 32 thread server the mean was 656 s (n=500).

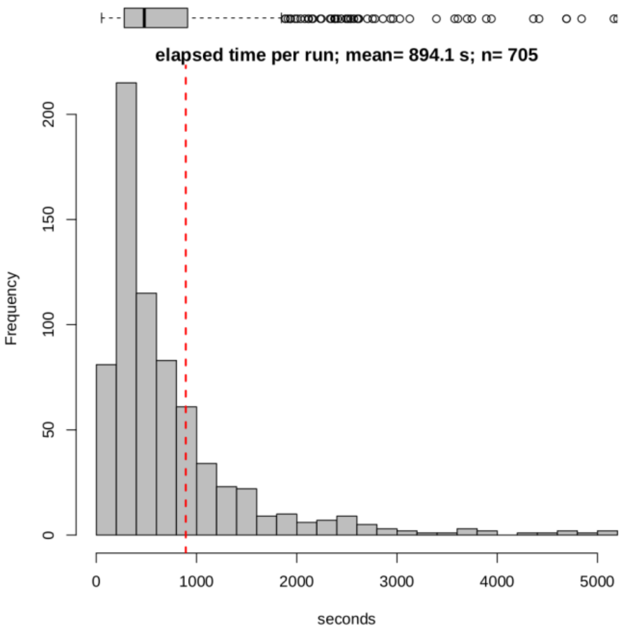

Assuming that this small subset is representative of the 581094 runs, it would take ~16.5 years to regenerate DEE2 on a single 16 thread server.

Using equivalent Amazon cloud resources and assuming the performance is equivalent, it would cost approximately USD $97,000 to complete this analysis in a year with on-demand servers or USD $24,000 using the cheaper spot price which is limited to off peak times, availability and maximum instance time limits.

Information to this effect has been added on page 13 of the manuscript.

"... the estimated cost to process these datasets on Amazon EC2 infrastructure is estimated at just $97,000 but could be reduced to about ~$24,000 using off-peak resources[c]."
