# Supplementary material for: Digital expression explorer 2: a repository of uniformly processed RNA sequencing data
Source: Gigascience. 2019 Apr 3;8(4):giz022. doi: 10.1093/gigascience/giz022 (PMC6446219; doi:10.1093/gigascience/giz022)
Supplement: Supplement_Tables.pdf [file giz022_supplement_tables.pdf]

Supplementary Table 1. Software versions and parameters used in the pipeline.

Software, version	Purpose	Parameter	
		Single end	Paired end
Aspera client, v3.5.4	Rapid download of sequence data	ascp -l 500m -O 33001 -T -i $ID $URL .	
SRA toolkit, v2.8.2	Validate downloaded SRA files	vdb-validate $SRA	
	diagnose single or paired end	fastq-dump -X 4000 --split-files $SRA	
	dump fastq	(see parallel-fastq-dump below)	
FastQC, v0.11.5	Diagnose basespace / colorspace, quality encoding, read length from 4000 reads	fastqc $FQ1	fastqc $FQ2
parallel-fastq-dump, 0.6.3	Rapid decompression of sequence data from .sra files	parallel-fastq-dump --threads $THREADS --outdir . --split-files --defline-qual + -s ${SRR}.sra	
Skewer, v0.2.2	3' quality trimming	skewer -l 18 -q 10 -k inf -t $THREADS -o $SRR $FQ1	skewer -l 18 -q 10 -k inf -t $THREADS -o $SRR $FQ1 $FQ2
	Adapter clipping	skewer -l 18 -t $THREADS -x $ADAPTER -o $SRR $FQ1	skewer -l 18 -t $THREADS -x $ADAPTER1 -y $ADAPTER2 -o $SRR $FQ1 $FQ2
	5' trimming	skewer -m ap --cut $CLIP_NUM,$CLIP_NUM -l 18 -k inf -t $THREADS $FQ1	skewer -m ap --cut $R1_CLIP_NUM,$R2_CLIP_NUM -l 18 -k inf -t $THREADS $FQ1 $FQ2
Minion, v13-100	3' adapter detection	minion search-adapter -i $FQ1	minion search-adapter -i $FQ2
Bowtie2, v2.3.2	Adapter contamination detection	bowtie2 -f -x $BT2_REF -S /dev/stdout $ADAPTER	
FASTX-Toolkit, v0.0.14	Progressive 5' trimming	fastx_trimmer -f {5,9,13,21} -m 18 -Q 33 -i $FQ1	fastx_trimmer -f {5,9,13,21} -m 18 -Q 33 -i $FQ2
STAR v020201	Gene-level mapping, Diagnose strandedness	STAR --runThreadN $THREADS --quantMode GeneCounts --genomeLoad LoadAndKeep \ --outSAMtype None --genomeDir $STAR_DIR --readFilesIn=$FQ1	STAR --runThreadN $THREADS --quantMode GeneCounts --genomeLoad LoadAndKeep \ --outSAMtype None --genomeDir $STAR_DIR --readFilesIn=$FQ1 $FQ2
Kallisto, v0.43.1	Transcript-level mapping	kallisto quant $KALLISTO_STRAND_PARAMETER \ --single -l 100 -s 20 -t $THREADS -o . \ -i $KAL_REF $FQ1	kallisto quant $KALLISTO_STRAND_PARAMETER -t $THREADS -o . -i $KAL_REF $FQ1 $FQ2

Supplementary Table 2. Spearman correlation coefficients (ρ) between ground truth and DEE2 processed expression profiles (RPM) from simulated data.

Species	Seq format	STAR (gene)	Kallisto (transcript)	Kallisto (gene)
A. thaliana	50 bp SE	0.958	0.788	0.998
	100 bp SE	0.957	0.744	0.998
	50 bp PE	0.950	0.722	0.999
	100 bp PE	0.948	0.697	0.997
C. elegans	50 bp SE	0.939	0.805	0.988
	100 bp SE	0.946	0.769	0.984
	50 bp PE	0.939	0.755	0.985
	100 bp PE	0.940	0.699	0.981
D. melanogaster	50 bp SE	0.913	0.836	0.997
	100 bp SE	0.912	0.795	0.997
	50 bp PE	0.905	0.757	0.998
	100 bp PE	0.905	0.731	0.997
D. rerio	50 bp SE	0.924	0.926	0.997
	100 bp SE	0.947	0.907	0.997
	50 bp PE	0.939	0.911	0.998
	100 bp PE	0.953	0.887	0.997
E. coli	50 bp SE	0.980	0.996	0.996
	100 bp SE	0.981	0.999	0.999
	50 bp PE	0.980	0.999	0.999
	100 bp PE	0.982	0.999	0.999
H. sapiens	50 bp SE	0.926	0.822	0.988
	100 bp SE	0.934	0.795	0.990
	50 bp PE	0.922	0.782	0.991
	100 bp PE	0.926	0.767	0.989
M. musculus	50 bp SE	0.924	0.897	0.991
	100 bp SE	0.937	0.868	0.991
	50 bp PE	0.928	0.867	0.993
	100 bp PE	0.936	0.839	0.992
R. norvegicus	50 bp SE	0.882	0.957	0.992
	100 bp SE	0.895	0.946	0.992
	50 bp PE	0.890	0.944	0.993
	100 bp PE	0.897	0.925	0.990
S. cerevisiae	50 bp SE	0.992	0.980	0.980
	100 bp SE	0.929	0.981	0.981
	50 bp PE	0.923	0.986	0.986
	100 bp PE	0.927	0.980	0.980
