# Supplementary material for: Digital expression explorer 2: a repository of uniformly processed RNA sequencing data
Source: Gigascience. 2019 Apr 3;8(4):giz022. doi: 10.1093/gigascience/giz022 (PMC6446219; doi:10.1093/gigascience/giz022)
Supplement: Response-to-Reviewer-Comments_Original-Submission.pdf [file giz022_response-to-reviewer-comments_original-submission.pdf]

Author's Response To Reviewer Comments

Close

Dear Dr Edmunds,

We thank both reviewers for their thorough review and highly constructive suggestions which have improved this body of work markedly.

In the Personal Cover docx document we uploaded, we have addressed each of the reviewers' points (#1 to #37). To make it clear what changes have been made to the manuscript we have provided a copy of the manuscript with changes annotated with numbered comments (#1 to #37) in addition to a "clean" version.

(the response to reviewers' comments document is 15 pages and contains several tables and graphs, so we uploaded it as a separate docx document)

Also in accordance with editorial guidelines, we have added SciCrunch RRIDs for essential DEE2 resources:
1) SCR_016931 Docker Image
2) SCR_016930 Source Code
3) SCR_016929 Project Homepage

We certainly hope that the revised manuscript is suitable for publication in GigaScience.

Sincerely,

Mark Ziemann, PhD
Deakin University

Close
