# Supplementary material for: Digital expression explorer 2: a repository of uniformly processed RNA sequencing data
Source: Gigascience. 2019 Apr 3;8(4):giz022. doi: 10.1093/gigascience/giz022 (PMC6446219; doi:10.1093/gigascience/giz022)
Supplement: Reviewer-1-Report-Original-Submission -- Lucas Carey — 12/4/2018 Reviewed [file giz022_reviewer-1-report-original-submission.pdf]

Reviewer Report

Title: Digital Expression Explorer 2: a repository of uniformly processed RNA sequencing data

Version: Original Submission **Date:** 12/4/2018

Reviewer name: Lucas Carey

Reviewer Comments to Author:

Basically every lab in the world is doing RNA-seq, and they are all depositing their data on NCBI SRA. The result is over a million RNA-seq experiments that can be analyzed by anyone. However, this treasure-trove is impossible to mine unless you can use a high performance compute cluster and do some somewhat complex scripting. Far more biologists can open a data matrix in excel, R, python or matlab. Therefore the large-scale systematic conversion of raw RNA-seq reads into data-matricies enables work that would otherwise be impossible, or not worth the effort, for the individual (computational) biologist. Indeed, we used DEE to identify the molecular mechanism (gene) driving an expression phenotype of interest. We could not have done this without the DEE.
Our hope is actually that DEE v3 become unnecessary, as NCBI should incorporate this feature into SRA. Until then, we hope that more biologists will use DEEv2, which has the potential to be valuable resource for a broad community.
We have a few major points that need to be solved prior to publication. And a few suggestions / ideas that are not needed for publication.
Lucas Carey Miquel-Angel Schikora
Major points:
The right side of Fig3C is cut off.
Page 8 : "in contrasts that had greater sequencing depth and more replicates. " in experiments ?
I don't see the metadata ("Keyword context") in the downloaded tab files. This information is essential for processing, filtering and interpreting the downloaded data. Please include it. You may consider replacing the "Keyword context" field by "Accession description", which would make the selection of runs of interest easier. Also include this information in both the output of "Search accession numbers" in the webpage and the getDee2Metadata() function of the R API.
Include a table with transcript and gene lengths so that FPKM type numbers can be calculated. and/or a the file or a link to download the gene annotation files that you use for each species.
Searching with multiple keywords doesn't work. This has to be implemented. At least AND. This is a problem because the user can't download results if there are >500 matches. For example, if I search for the gene CLN3 in S cerevisiae I get 550 matches, and I can neither download all the data nor limit my search with AND, OR, or NOT keywords.
Figure 5, and to a lesser extent, Fig 3, are very low resolution. Especially Figure 5.
The criteria used for quality control pass/fail are not clearly stated. For example, how was the "optimal number of bases to clip from the 5' end" calculated? Which read summarization method was used (union, intersection of all, the intersection of all non-empty)?
Figure 4: The legend is not clear enough. Why does 4A have error-bars and 4B does not? Is this because

4A includes all experiments (conditions and replicates), while 4B is the single differential expression value? I think 4A is a boxplot of correlation across all samples. But B shows a single value, even for GEOs that have multiple conditions, and therefore multiple differential expression values. Expand the text to make how these values were calculated more clear.
In Table S1, S. cerevisiae 50 bp PE has a much worse correlation (r = 0.46) than any other sample or species. What is the reason for this? This looks like a bug.
The definition of each of the contents of the "QC summary" field in the web search results table should be provided somewhere. Similar definitions should be included in the R API.
In the "Quality control and data validation" section, the authors describe the obtention of gene counts based on "Kallisto transcript counts collapsed into their parent gene", which turn to be more accurate than STAR counts. However, these counts can't be obtained through the web or the R API. The reason for this should be clarified, and the method to reproduce this result available. The user should be able to download the gene-transcript table. Was the calculation simply the sum of counts from all transcripts that 'belong' to a given gene? Or something more complicated?
The accession SRA355722 does not give any results for A. thaliana in the web server even though it is provided as an example on the website. The authors should make sure that all the website examples work.
In the figure 3 legend, it is unclear what ART means. Adding a citation will make this clear.
The acronyms UMI and QC should be defined when they're used the first time.
Minor and optional points:
"the datasets will be available publicly. In this way, power users obtain benefit by using an established analysis pipeline and simultaneously contribute..."
I think this is VERY clever, and could be highlighted more. Other projects could learn from this.
Mouse-over on QC doesn't work if you have >500 matches. This is confusing and should be fixed or just remove the mention of mouse-over in the text.
When a search returns >500 matches, an option to download the first 500 would be nice.
With regards to quality control: It would be very useful to be able to filter by minimum correlation with the bulk of the experiments for a given species. In our hands, with DEEv1, most S cerevisiae experiments had an inter-experiment correlation > 0.5; these appeared to be "normal" mRNA-seq experiments according to the metadata. A subset of experiments (~10%) have very low correlation with any other experiment, and are probably garbage. This might be a nice quality control metric to include in the search.
It would be very interesting, and therefore possibly increase usage, to use the DEE to make one or two points. For example, why do some experiments have such low correlation with the bulk of the data (see above)? Are they not really mRNA-seq? Different method? Not from this species?
Page 3, line 1: In the sentence "RNA-seq has become as a powerful method in transcriptomics", "as" should be removed.
The "Figure 1" text in the lower left corner of Figure 1 is redundant and may be removed.
Figure 3 would benefit from a larger font size in the axis labels. In addition, figure 3 plots would be easier to interpret with a density scatterplot, which would allow to see where the majority of the genes/transcripts are. There's no need to separate the >2 fold and <2 fold regions with colors, as dashed lines could serve the same purpose. Would also be nice to see the % of genes/transcripts in each

category.
In figure 3, log2(simulated) values (x axis) are the expected expression values. All data are simulated. The figures would be more clear having the "log2(expected expression)" as X axis and "log2(DEE2-pipeline-inferred expression)" as Y axis.
The sentence "Correlation was higher in contrasts that had greater sequencing depth and more replicates" should be supported with data.
It is unclear how were the Figure 4 datasets chosen. How do we know that they did not just picked the best experiments of each species? I encourage the authors to use multiple experiments per species, randomly selected from datasets with pre-calculated expression values. This will eventually make the argument that "Both validation exercises demonstrate the accuracy of DEE2 data".

Level of Interest

Please indicate how interesting you found the manuscript: Choose an item.

Quality of Written English

Please indicate the quality of language in the manuscript: Choose an item.

Declaration of Competing Interests

Please complete a declaration of competing interests, considering the following questions:

- Have you in the past five years received reimbursements, fees, funding, or salary from an organisation that may in any way gain or lose financially from the publication of this manuscript, either now or in the future?
- Do you hold any stocks or shares in an organisation that may in any way gain or lose financially from the publication of this manuscript, either now or in the future?
- Do you hold or are you currently applying for any patents relating to the content of the manuscript?
- Have you received reimbursements, fees, funding, or salary from an organization that holds or has applied for patents relating to the content of the manuscript?
- Do you have any other financial competing interests?
- Do you have any non-financial competing interests in relation to this paper?

If you can answer no to all of the above, write 'I declare that I have no competing interests' below. If your reply is yes to any, please give details below.

'I declare that I have no competing interests'

I agree to the open peer review policy of the journal. I understand that my name will be included on my report to the authors and, if the manuscript is accepted for publication, my named report including any

attachments I upload will be posted on the website along with the authors' responses. I agree for my report to be made available under an Open Access Creative Commons CC-BY license (http://creativecommons.org/licenses/by/4.0/). I understand that any comments which I do not wish to be included in my named report can be included as confidential comments to the editors, which will not be published.

I agree to the open peer review policy of the journal

To further support our reviewers, we have joined with Publons, where you can gain additional credit to further highlight your hard work (see: https://publons.com/journal/530/gigascience). On publication of this paper, your review will be automatically added to Publons, you can then choose whether or not to claim your Publons credit. I understand this statement.

Yes Choose an item.
