# Supplementary material for: Digital expression explorer 2: a repository of uniformly processed RNA sequencing data
Source: Gigascience. 2019 Apr 3;8(4):giz022. doi: 10.1093/gigascience/giz022 (PMC6446219; doi:10.1093/gigascience/giz022)
Supplement: Reviewer-2-Report-Original-Submission -- Andrea Rau — 12/10/2018 Reviewed [file giz022_reviewer-2-report-original-submission.pdf]

Reviewer Report

Title: Digital Expression Explorer 2: a repository of uniformly processed RNA sequencing data

Version: Original Submission **Date:** 12/10/2018

Reviewer name: Andrea Rau

Reviewer Comments to Author:

Ziemann et al. describe the Digital Expression Explorer 2 (DEE2), a data compendium containing publically available RNA-seq data arising from 9 different species (corresponding to over 470,000+ different samples) that have been uniformly re-processed to facilitate re-analysis. DEE2 is a multi-species resource that represents a useful contribution to other existing massive compendia of re-processed RNA-seq data that have primarily focused on human and mouse experiments to date (e.g., recount2, Expression Atlas, ARCHS4, Toil, RNAseqDB, etc.).

In my own work (for methodological development in gene co-expression analyses), I used the previous iteration of the Digital Expression Explorer prior to its new upgrades; a variety of novel and useful elements have since been added to DEE2. Real advantages in the new version include the availability of a summary of quality statistics as well as both gene-level counts and transcript-level quantifications, a new Docker image allowing end users to process data using the DEE2 pipeline (and a mechanism to include them in the publically available DEE2 repository server), smart adaptor clipping, strandedness, and paired-end analysis. Users can search the repository according to keywords or SRA IDs, and data are then made available directly through the web browser, command line, or bulk dumps. The DEE 2 website itself is very accessible and user-friendly, and contains a wealth of information (YouTube video, tutorials, FAQ page, links to relevant blog posts by the authors) that would be helpful for new users. In addition, the structure of the website accommodates both bulk and single study downloads. The processed DEE2 files can be easily read into R and fed into established differential analysis pipelines (DESeq2, edgeR, limma-voom, Degust).

All in all, I think DEE2 is a useful resource, and the scale of work needed to create this data compendium is notable. I have a few suggestions below to improve clarity and detail about the pipeline, simulations, and data output, as well as some minor comments and a few discretionary comments/suggestions for the authors' consideration.

** Major comments **

-- I was not able to find any information about the specific software versions or parameters that were used in data re-processing, but these represent critical pieces of information with respect to reproducibility. The DEE2 webpage provides only the reference genome sequence and annotation versions (http://dee2.io/about.html), and links to the GitHub repo (https://github.com/markziemann/dee2) for details on the processing pipeline; from what I can tell, this repo contains the source scripts for the pipeline (from which one can thus find the parameters used for each method), but versions are not included here. From this, I was able to figure out that, for example, STAR was run with --quantMode GeneCounts to obtain gene-level counts; however, this information should be much more obvious and ideally should be included on the website or in the output files

themselves. As a related note, perhaps DEE2 should itself also be versioned for reference so that results can easily be referenced if annotation/software versions are updated.
-- Some relevant details are occasionally missing from the paper. For instance, are the kallisto transcript values the TPM values (i.e. normalized by genomic feature effective lengths?) or the est_counts values? This detail was unclear to me. Similarly, on page 7 line 1, the authors discuss generating "synthetic Illumina HiSeq RNA-seq data", but don't mention the tool they use for this purpose (ART) except in the caption for Figure 3, leading to some confusion for the reader. Some minimal details about their simulation (number of genes, read length, coverage etc.) should be included, at least in Supplementary Materials.
-- Unless I've misunderstood, there would be no way to read in the kallisto transcript estimates using sleuth or tximport (which are both state-of-the-art tools for importing and summarizing transcript estimates to gene-level), as the original format of these files was not retained (i.e., they are now wide matrices containing only estimated transcript counts, rather than long matrices including length, eff_length, est_counts, tpm). All of the documentation appears to focus on the use of the gene counts; do the authors have another intended downstream use for the kallisto transcript estimates?
-- In the previous iteration of this tool, there was a SraRunTable text file with sample-meta data that was also downloaded with count matrices. This information is obviously critical in order to account for batch effects and appropriately model the experimental design in downstream analyses. I greatly appreciate the easy integration of DEE2 with the SRAdbV2 interface for NCBI SRA metadata, and this will be useful for re-analysis of data from within R. Is there any similarly easy way for a non-R user to obtain these tables (downloaded data from the website only include gene counts, transcript counts, QC, and related logs)?
-- Is there any intention to add other species? I particularly have in mind agriculturally relevant species such as chicken, pigs, and cattle, as I think such a resource could be very valuable for researchers in the animal genetics community. Also, given the large availability of human/mouse reprocessed data in other large-scale data compendia, is there any reason that so many DEE2 resources have been dedicated to re-analyzing these species (5787+5737 = 78% of the DEE2 datasets) rather than focusing resources on other species?
** Minor comments **
-- I think it could be useful to cite BgeeDB (https://bgee.org; https://bioconductor.org/packages/release/bioc/html/BgeeDB.html) and briefly situate that work with respect to the current work. Like the current work, Bgee includes a variety of species other than human and mouse; however, unlike the current work, it instead focuses on normalized data arising from a variety of platforms in multiple tissues/life stages of curated normal samples to facilitate cross-species and cross-tissue analyses.
-- Does Supplementary Table 1 represent the median Spearman correlation coefficients across simulated genes? And are these correlations (as well as those in Figure 4A) based on counts, or log-counts? In Figure 4B, what is plotted? Median (across all genes) Spearman correlation between estimated log-fold changes for DEE2-STAR and author-suppled counts for a single chosen dataset? Why not use boxplots for these as well?
-- Are there datasets for which the QC summary is WARNING or FAIL instead of PASS (and if so, do you have general statistics on the number of passing/failing datasets)? After a quick browse through some of

the species, they all appeared to be flagged as PASS. Does an experiment get flagged as FAIL if a single QC metric fails (or in other words, what is the threshold for flagging an experiment as failing QC)? In any case, I like that all of the statistics are available to the user, allowing each person to decide whether to include a dataset or not.
** Discretionary comments **
-- One minor point that I hesitate to mention (given the large amount of work the authors have already put in), but that could significantly strengthen the paper: a small example to illustrate a meta-analysis using processed DEE2 data would be wonderful to illustrate the ease and benefit of the DEE2 compendium.
-- Sourcing an R script such as getDEE2.R from the web is a fairly insecure practice; see https://www.jumpingrivers.com/blog/security-r-hacking-bioconductor for a blog discussing this. After having used a source command for package installation for many years, Bioconductor has actually recently switched to the new BiocManager package (https://cran.r-project.org/web/packages/BiocManager/vignettes/BiocManager.html) as a safer way to install and manage Bioconductor packages. I wonder whether it would be worth bundling your R access scripts into a minimal package on GitHub that could then in turn be installed using install_github() from devtools?
-- There are some small inconsistencies in the explanation page at https://github.com/markziemann/dee2/blob/master/AccessDEEfromR.md. For example, the metadata are loaded into an object called 'mdat', and the subsequence line of code looks at the head of 'metadata'. This could be rectified if the document was instead written as a reproducible Rmd document.
-- The authors discus the financial cost of regenerating these experimental data in the "Re-use potential" section. It would also be interesting, if possible, to break down what the total computational cost (in terms of $$ or time) of creating the DEE2 compendium is/was. This could help provide an additional selling point for the great value of making available these re-processed data, thus allowing researchers to avoid unnecessarily re-doing these same steps themselves.

Level of Interest

Please indicate how interesting you found the manuscript: Choose an item.

Quality of Written English

Please indicate the quality of language in the manuscript: Choose an item.

Declaration of Competing Interests

Please complete a declaration of competing interests, considering the following questions:

- Have you in the past five years received reimbursements, fees, funding, or salary from an organisation that may in any way gain or lose financially from the publication of this manuscript, either now or in the future?

- Do you hold any stocks or shares in an organisation that may in any way gain or lose financially from the publication of this manuscript, either now or in the future?
- Do you hold or are you currently applying for any patents relating to the content of the manuscript?
- Have you received reimbursements, fees, funding, or salary from an organization that holds or has applied for patents relating to the content of the manuscript?
- Do you have any other financial competing interests?
- Do you have any non-financial competing interests in relation to this paper?

If you can answer no to all of the above, write 'I declare that I have no competing interests' below. If your reply is yes to any, please give details below.

I declare that I have no competing interests.

I agree to the open peer review policy of the journal. I understand that my name will be included on my report to the authors and, if the manuscript is accepted for publication, my named report including any attachments I upload will be posted on the website along with the authors' responses. I agree for my report to be made available under an Open Access Creative Commons CC-BY license (http://creativecommons.org/licenses/by/4.0/). I understand that any comments which I do not wish to be included in my named report can be included as confidential comments to the editors, which will not be published.

I agree to the open peer review policy of the journal

To further support our reviewers, we have joined with Publons, where you can gain additional credit to further highlight your hard work (see: https://publons.com/journal/530/gigascience). On publication of this paper, your review will be automatically added to Publons, you can then choose whether or not to claim your Publons credit. I understand this statement.

Yes Choose an item.
